# A switch-on luminescent europium(iii) probe for selective and time-resolved detection of adenosine diphosphate (ADP)†

**DOI:** 10.1039/d4sc07188c

**Published:** 2025-02-19

**Authors:** Samantha E. Bodman, Patrycja Stachelek, Umatur Rehman, Felix Plasser, Robert Pal, Stephen J. Butler

**Affiliations:** a Department of Chemistry, Loughborough University Epinal Way Loughborough LE113TU UK s.j.butler@lboro.ac.uk; b Department of Chemistry, Durham University Durham DH1 3LE UK

## Abstract

Adenosine diphosphate (ADP) is a key product of two essential classes of biological reactions, catalysed by ATPases and kinases. This makes ADP a highly appealing target for supramolecular detection. However, doing so selectively is exceedingly difficult due to ADP's lower overall charge and similar structure to ATP and the need for compatibility with biological media. Overcoming this challenge, here we present a water-soluble, ADP-selective, luminescent europium(iii) probe suitable for use *in vitro* and in cellular microscopy. This negatively charged Eu(iii) complex binds ADP reversibly and responds by switching on its luminescence, whilst showing minimal interference from ATP, pyrophosphate and a wide range of biological anions. The probe is equipped with two π-conjugated quinolyl-phenoxyacetate antennae, facilitating excitation at 355 nm in fluorescence microscopy. The ancillary carboxylate groups ensure high water solubility and suppress non-specific binding to albumin protein. Our novel probe demonstrates a level of sensing selectivity for ADP that is unrivaled, producing a linear emission response across the physiologically relevant concentration range (10–400 μM), even in the presence of excess millimolar ATP. We demonstrate that this amphiphilic Eu(iii) probe permeates mammalian cells and localises within the mitochondria and lysosomes. The low background emission of the probe combined with its excellent ADP selectivity and long-lived luminescence makes it a promising tool for visualising ADP levels in living cells.

## Abstract 2

We present a novel luminescent europium(iii) probe for highly selective ADP detection with minimal ATP interference. It enables unprecedented ADP sensing in physiologically relevant ranges, facilitating *in vitro* and cellular imaging applications.

## Graphical abstract

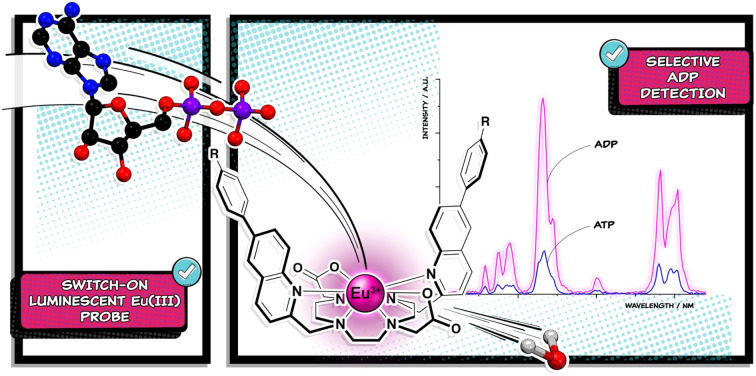

## Introduction

Adenosine diphosphate (ADP) is produced during two of the most fundamental classes of biological reactions, which are catalysed by ATPases and kinases. The hydrolysis of ATP to ADP by ATPases releases energy that is used for a wide variety of cellular processes, whereas kinases catalyse the transfer of the terminal phosphate of ATP onto a protein and small molecule substrate, and play major roles in cell differentiation, proliferation, apoptosis and signal transduction. Misregulation of kinase activity is a major cause of a range of diseases including cancer. Therefore, a probe capable of selectively detecting ADP would have broad utility as a tool to measure the activity of any ATPase or kinase.^1,2^ This is crucial to understanding biochemical mechanisms in cells that lead to the origin of disease and would enable better identification of targets for therapeutic intervention.^3^

Despite significant efforts to develop fluorescent tools for the detection of ADP, the number of ADP-selective probes remains very limited.^4–6^ Designing such probes is very challenging due to the abundance of structurally similar biological phosphate species, especially the more highly charged anions ATP and pyrophosphate (PPi). Indeed, the majority of fluorescent probes demonstrate a selectivity order of ATP ∼ PPi > ADP > AMP, as electrostatics predominantly govern the binding free energy in these receptor–anion complexes.^7–11^

Notably, Feng and co-workers reported an anthracene-based mononuclear zinc(ii) probe that showed unexpected selectivity for ADP, however the limited water solubility and modest (1.3-fold) fluorescence enhancement precluded its use in cellular imaging applications.^12^ The same authors developed a water-soluble anthracene bridged bis-zinc(ii)-DPA probe, which showed a much larger 133-fold fluorescence enhancement upon binding ADP. However, the probe showed interference from ATP, resulting in a 56-fold fluorescence enhancement upon ATP binding.^13^ Webb and coworkers developed an ADP-selective biosensor based on bacterial actin homologue (ParM) labelled with two tetramethylrhodamine groups, which undergoes a conformational change upon binding ADP inducing a 15-fold fluorescence enhancement.^14^ This enabled the kinase-catalysed production of ADP to be monitored *in vitro*, however its application in living cells has not been demonstrated. Yellen and coworkers developed a genetically encoded biosensor ‘PercevalHR’ that binds strongly to both ATP and ADP (*K*_d_ = 1–3 μM) and can monitor changes in the ATP/ADP ratio in living cells, producing a maximum 8-fold change in emission signal.^15^ However, as with most protein-derived sensors, PercevalHR is sensitive to changes in pH, requiring additional methods to correct for changes in biosensor fluorescence caused by pH.^15,16^

Luminescent lanthanide probes have emerged as promising tools for anion sensing and imaging, due to their unique photophysical properties,^17–21^ including long luminescence lifetimes (milliseconds) that permit use of time-resolved and time-gated techniques to completely remove short-lived autofluorescence,^22^ line-like emission spectra which enable ratiometric measurements of target anions, and a fast and sensitive luminescence response that provides high spatial and temporal resolution required for biological imaging.^23,24^

**Fig. 1:**
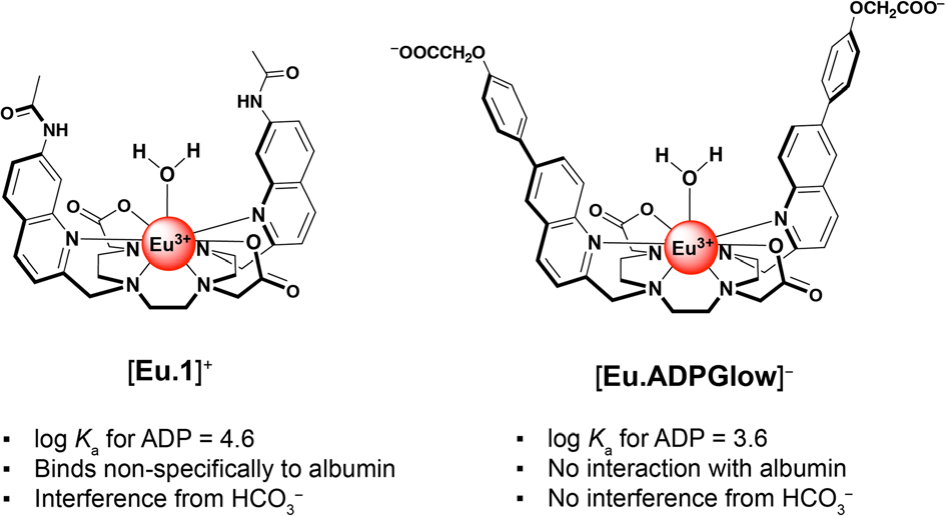
(left) Structure of previously synthesised probe [Eu.1]^+^ and (right) the novel probe [Eu.ADPGlow]^−^ presented in this work.

We previously developed a macrocyclic europium probe [Eu.1]^+^ (Fig. 1a) whose luminescence is enhanced 8-fold upon reversible binding of ADP, whereas ATP induces a smaller 2.5-fold emission increase and monophosphates (*e.g.* AMP, HPO_4_^2−^) cause negligible changes in luminescence.^25^

Key structural elements that ensure selectivity for ADP include two *trans*-related quinoline arms orientated from the same face of the *C*_2_-symmetric macrocyclic ligand, forming a sterically shielded binding site at the metal centre suitable for bidentate binding of the diphosphate component of ADP.^26,27^ Additionally, the peripheral quinoline amide groups can engage in hydrogen bonding with ADP, further stabilising the host–guest complex. [Eu.1]^+^ can accurately monitor kinase and ATPase catalysed reactions in real-time by monitoring the increase in ADP *in vitro*.^28^ However, this probe is not suitable for visualising ADP levels in living cells due to its propensity to bind bicarbonate^29^ (an abundant biological anion) and interact non-specifically with albumin protein, leading to high background emission. We aimed to resolve these issues of off-target interference while preserving the probe's ADP-selective binding capabilities.

Aside from demonstrating excellent selectivity, a cellular imaging probe for ADP should permeate living cells while exhibiting low toxicity and high photostability. Additionally, it should possess an excitation wavelength above 350 nm to enable use of standard microscope lasers. Due to the symmetry forbidden nature of f–f orbital transitions, direct excitation of the europium ion is not efficient or practical.^30^ Instead, the europium excited state is populated *via* a sensitisation mechanism, involving initial UV light absorption by the antenna followed by intramolecular energy transfer to the Eu(iii) ion.^31,32^ Changes in the structure of the antenna impact the excitation wavelength, extinction coefficient, sensitisation efficiency and overall brightness *B* (*B* = *ε*·*ϕ*_em_ where *ε* is the extinction coefficient and *ϕ*_em_ the quantum yield). In the last decade, highly emissive europium complexes have been devised for live-cell imaging, including the EuroTracker dyes based pyridyl-ethynyl-alkoxyphenyl charge-transfer antennae.^33,34^ Maury and co-workers harnessed the π-conjugated charge-transfer antennae to develop very bright lanthanide two-photon probes, wherein the excitation wavelength is doubled in microscopy experiments, reducing biological photodamage of cells.^24,35^

By combining the ligand design features of the ADP-selective probe [Eu.1]^+^ with the beneficial properties of π-conjugated charge-transfer antennae, we have developed a novel europium(iii) probe with unrivalled ADP sensing selectivity and with improved characteristics for cellular imaging. This new probe, [Eu.ADPGlow]^−^ (Fig. 1b), features two π-conjugated quinolyl-phenoxyacetate pendant arms extending from the macrocycle, creating a sterically shielded coordination site at the Eu(iii) centre. The probe includes two ancillary carboxylate groups to ensure high water solubility and suppress non-specific binding to albumin protein. The overall negative charge of the complex results in moderate ADP binding affinity (log *K*_a_ = 3.59 ± 0.01) and induces a pronounced luminescence ‘switch on’, with minimal interference from the more highly charged ATP and PPi. The probe's excellent selectivity towards ADP enables its detection across a wide concentration range (10–500 μM) even in the presence of high millimolar ATP concentrations. Preliminary cellular microscopy experiments demonstrate that the Eu(iii) probe can permeate mammalian cells and preferentially distributes within the mitochondria and lysosomes. Combined with its almost zero background emission in the absence of ADP, these features make this probe a promising tool for visualising dynamic changes in cellular ADP levels in living cells.

## Results and discussion

### Molecular design

The Eu(iii) probe [Eu.ADPGlow]^−^ (Fig. 1b) is based on a cyclen scaffold containing two *trans*-oriented π-conjugated quinoline antennae functionalised in the 6-position with water-solubilising phenoxyacetate groups. Intra-ligand charge transfer was anticipated from the terminal electron donating –OCH_2_COOH moieties to the electron withdrawing chelating quinoline groups, offering potentially high extinction coefficients and lower energy two-photon excitation.^36,37^ The cyclen macrocycle is functionalised with two *trans*-related acetate arms (DO2A scaffold) that coordinate the central Eu(iii) ion together with the quinoline groups, ensuring high thermodynamic stability. The Eu(iii) metal centre has a vacant coordination site that is occupied by a water molecule, which efficiently quenches the Eu(iii) excited state through energy transfer to O–H vibrations, resulting in non-radiative energy dissipation.^30,38^ It was envisaged that ADP would bind to the metal centre in its preferred bidentate mode,^26^ causing reversible de-coordination of one of the quinoline arms.

**Scheme 1:**
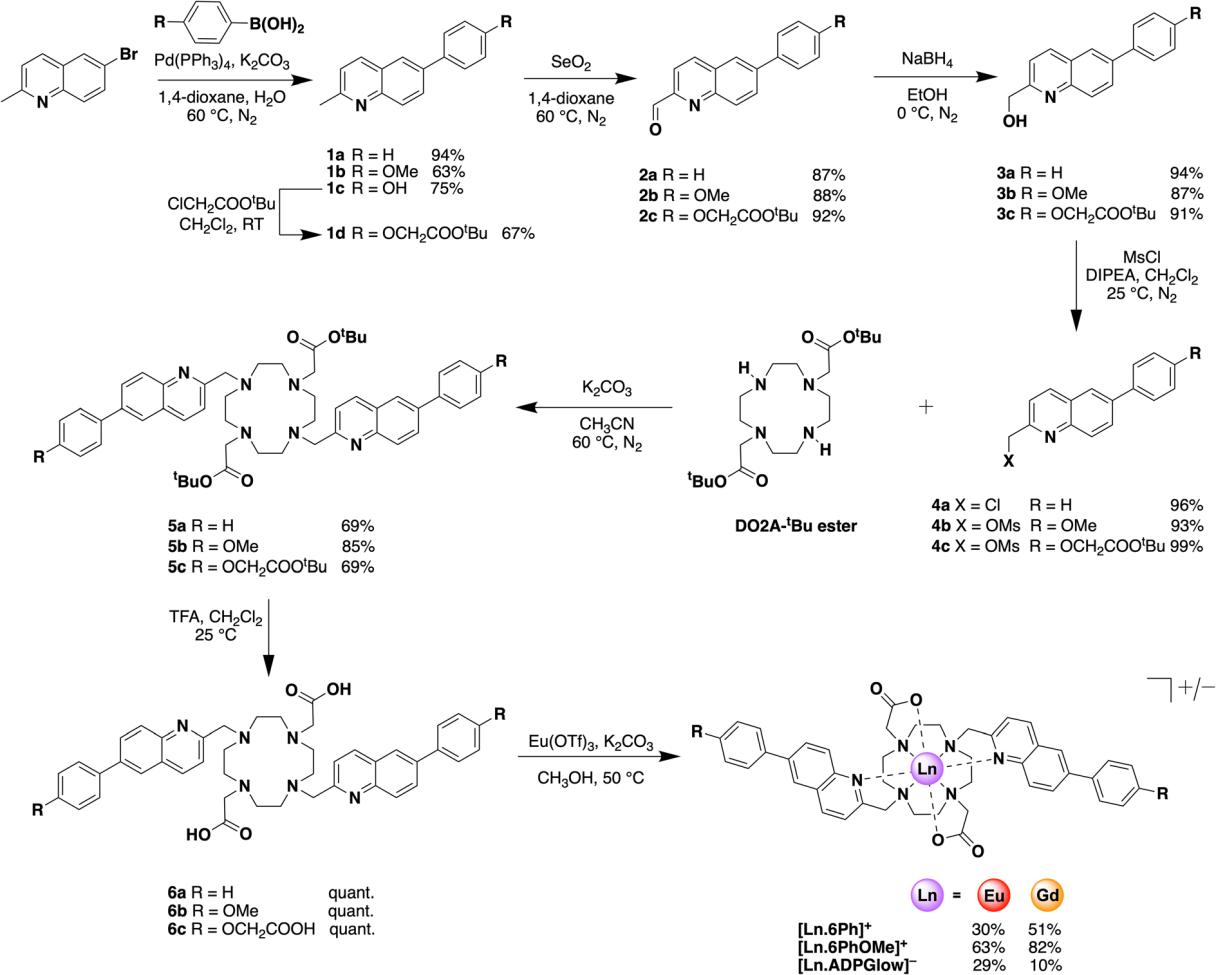
Synthesis of europium(iii) and gadolinium(iii) complexes.

We reasoned that by introducing peripheral carboxylate groups, and removing the hydrogen-bond (amide) donor groups present in our previously reported ADP probe (Fig. 1a),^28^ we would decrease affinity for the more negatively charged ATP and PPi, whilst suppressing hydrophobic interactions with human serum albumin.^39,40^ Two control complexes were synthesised, [Eu.6Ph]^+^ and [Eu.6PhOMe]^+^, containing 6-phenyl and 6-methoxyphenyl functionalised quinoline groups respectively (Scheme 1), to determine the impact of these ancillary substituents on the solubility, excitation wavelength, overall brightness and anion binding properties of the europium(iii) complexes.

### Synthesis

The synthesis of the macrocyclic ligands and their corresponding Ln(iii) complexes (Ln = Eu and Gd) was undertaken as shown in Scheme 1. Full details of the experimental procedures are provided in the ESI.† Briefly, the synthesis commenced with a Suzuki coupling of 6-bromoquinaldine with the corresponding boronic acid functionalised at the *para*-position with phenyl, *p*-methoxyphenyl or *p*-hydroxyphenyl substituents respectively, to give the π-conjugated quinoline compounds 1a–c in moderate to high yields. A subsequent *O*-alkylation of 4-(2-methyl-4-quinolinyl)-phenol 1c gave the *tert*-butyl protected acetate arm 1d. Next, oxidation using selenium dioxide resulted in formation of aldehydes 2a–c followed by reduction with sodium borohydride to give the primary alcohols 3a–c in excellent yields. Subsequent mesylation to give compounds 4a–c and *N*-alkylation with DO2A-*tert*-butyl ester resulted in formation of the protected macrocycles 5a–c. Finally, the *tert*-butyl esters were removed using trifluoroacetic acid followed by complexation with Ln(OTf)_3_ in methanol to afford the target Ln(iii) complexes. The organic soluble complexes [Ln.6Ph]^+^ and [Ln.6PhOMe]^+^ were purified by column chromatography, whereas the water soluble complexes [Ln.ADPGlow]^−^ were purified by reverse-phase HPLC. The analytical RP-HPLC trace of the purified product revealed a single peak (Fig. S1 and S2†). The ^1^H NMR spectra of [Eu.6Ph]^+^ and [Eu.6PhOMe]^+^ measured in CD_3_OD were very similar, each revealing two sets of proton resonances consistent with the presence of two diastereomers in solution (Fig. S3†). The ^1^H NMR spectrum of [Eu.ADPGlow]^−^ was more complicated, displaying four sets of signals with increased exchange broadening. This suggests multiple conformations in solution, possibly involving reversible intermolecular association of the peripheral carboxylate group with the Eu(iii) metal centre at the millimolar concentration used for NMR analysis.

### Photophysical analysis

**Fig. 2:**
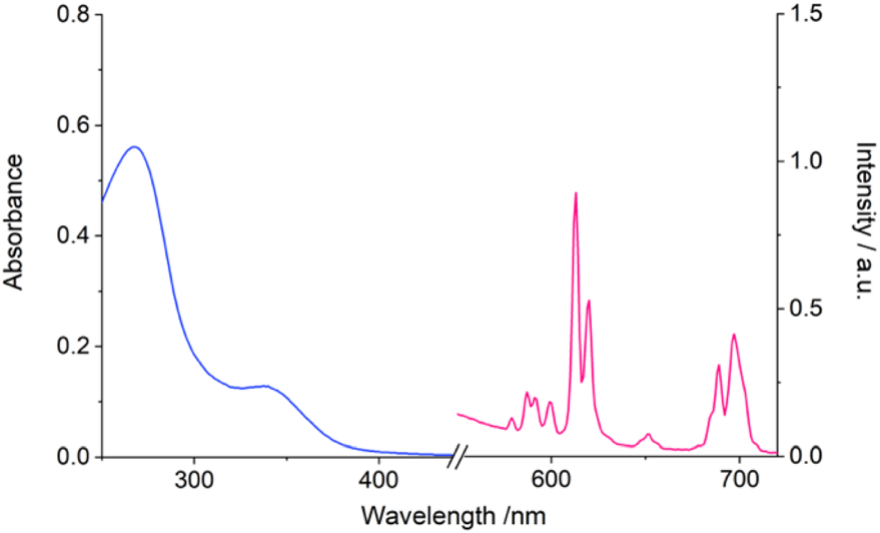
Absorption and emission spectra of [Eu.ADPGlow]^−^ (*λ*_ex_ 337 nm) measured in methanol.

Photophysical analysis of the water-soluble complex [Eu.ADPGlow]^−^, measured in 10 mM HEPES (pH 7.0, 295 K), is presented in Table 1. For direct comparison with complexes [Eu.6Ph]^+^ and [Eu.6PhOMe]^+^, which displayed limited water solubility, photophysical data were also recorded in methanol (see Table 1). The UV-vis absorption spectra of all three Eu(iii) complexes show two broad bands (Fig. 2 and S5†), with absorption maxima around 255 nm and 328 nm for [Eu.6Ph]^+^, and red shifted peaks around 275 nm and 340 nm for complexes [Eu.6PhOMe]^+^ and [Eu.ADPGlow]^−^, due to conjugation of the electron donating alkoxyphenyl groups with the quinoline acceptor groups. Notably, the lower energy absorption band of complex [Eu.ADPGlow]^−^ tails out to 380 nm in aqueous buffer (*ε* = 9200 M^−1^ cm^−1^), offering the best match for the optics of standard fluorescence microscopes used in live-cell imaging experiments, where the excitation wavelength is 355 or 405 nm.

**Table 1:** Photophysical data for Eu(iii) complexes measured in methanol to enable comparison. Additionally, [Eu.ADPGlow]^−^ was measured in 10 mM HEPES at pH 7.0 (top row)

Complex	Solvent	*λ*_max_/nm	*ε*/M^−1^ cm^−1^	*τ*_H_ a/ms	*τ*_D_ a/ms	*Φ*_em_ b/%
[Eu.ADPGlow]^−^	HEPES buffer	337	9200	0.021 ± 0.001	0.036 ± 0.001	0.3
[Eu.ADPGlow]^−^	MeOH	340	12 000	0.46 ± 0.01	0.75 ± 0.02	2.7
[Eu.6PhOMe]^+^	MeOH	340	8000	0.88 ± 0.09	1.16 ± 0.02	1.0
[Eu.6Ph]^+^	MeOH	328	9000	0.83 ± 0.04	1.30 ± 0.06	9.6

a *τ*_H_ refers to non-deuterated solvent, whereas *τ*_D_ refers to the deuterated solvent. Emission lifetime measurements were conducted in duplicate with errors determined through the averages ± standard deviation.

b Quantum yields were measured using quinine sulfate in 0.05 M H_2_SO_4_ as standard (*Φ*_em_ = 60%).^41^ Errors in quantum yields are ±15%.

Upon excitation of the π-conjugated quinoline antennae of [Eu.ADPGlow]^−^ at 340 nm the complex displays red europium emission in the range 550–720 nm (*Φ*_em_ = 2.7), well-separated from the absorption spectrum (Fig. 2). The emission features a dominant Δ*J* = 2 band characterised by two distinct peaks in the 605–630 nm range, which is much more intense than the purely magnetic dipole Δ*J* = 1 transition, observed between 585–605 nm (Fig. 2). The latter band consists of three distinct components. The emission intensity of [Eu.ADPGlow]^−^ was significantly weaker in 10 mM HEPES buffer (*Φ*_em_ = 0.3) than in methanol (Fig. S6†). As an anticipated luminescent probe for ADP, the high-water solubility and almost zero background emission signal of [Eu.ADPGlow]^−^ are significant advantages. The emission spectrum of the methoxy substituted complex [Eu.6PhOMe]^+^ was very similar in shape to that of [Eu.ADPGlow]^−^ in methanol (Fig. S6†), whereas [Eu.6Ph]^+^ differed showing a pronounced Δ*J* = 4 band (685–705 nm) of higher intensity than the Δ*J* = 2 band (605–630 nm). The unsubstituted complex [Eu.6Ph]^+^ was approximately 10 times more emissive than [Eu.6PhOMe]^+^. All three complexes showed some residual ligand fluorescence at shorter wavelength (Fig. S7†) which was most prominent for [Eu.6Ph]^+^ indicating different efficiencies of intramolecular energy transfer from the quinoline antennae to the europium(iii) centre for each complex. The emission lifetimes were similar for [Eu.6Ph]^+^ and [Eu.6PhOMe]^+^ in methanol, and approximately double that observed for [Eu.ADPGlow]^−^. In 10 mM HEPES buffer, the emission lifetime of [Eu.ADPGlow]^−^ was very short, at 0.021 ms (Table 1), such that estimation of the number of coordinated waters (*q* value) becomes difficult because the emission lifetime begins to overlap with the water exchange timescale.^42^ It is nonetheless significant that the emission lifetime was 1.7 times longer in deuterated HEPES buffer indicating that the complex is hydrated in the absence of anions, as expected.^26,39^

To better understand the intramolecular energy transfer in these conjugated Eu(iii) complexes, the triplet energy of the antennae was determined at 77 K using the analogous Gd(iii) complexes. Unlike Eu(iii), Gd(iii) has an excited state that is inaccessible to ligand triplet energy transfer, enabling direct measurement of the triplet excited state energy. Phosphorescence spectra of the gadolinium(iii) complexes were measured in EPA solvent, comprising ethanol/isopentane/diethyl ether in the ratio 2/5/5.^43^ The gadolinium(iii) complexes showed no emission at room temperature, but upon cooling to 77 K, all three complexes displayed phosphorescence with vibrational structure attributed to emission from the ligand-centred triplet excited state (Table S1†). No evidence of a broad, featureless internal charge transfer (ICT) state – characteristic of the bright Eu(iii) complex series bearing pyridyl-ethynyl-alkoxyphenyl antennae – was observed.^44–46^ From the position of the highest energy band in the spectrum, the triplet energy of [Gd.ADPGlow]^−^ in EPA (Fig. S8†) was determined to be 21 100 cm^−1^, which is appropriately positioned for sensitisation of the europium(iii) ^5^D_0_ and ^5^D_1_ accepting states (at 17 300 cm^−1^ and 19 000 cm^−1^, respectively). The triplet energy of the methoxy substituted complex [Gd.6PhOMe]^+^ was approximately 1500 cm^−1^ lower, at 19 600 cm^−1^, resulting in a poorer match with the acceptor states of the Eu(iii) ion.^47^ This is consistent with the lower quantum yield observed for this Eu(iii) complex (Table 1). For [Gd.6Ph]^+^, an intermediate triplet energy value of 20 100 cm^−1^ was determined.

### Stable emission of [Eu.ADPGlow]^−^ at physiological pH

The low background emission signal, high water solubility and longer excitation wavelength of complex [Eu.ADPGlow]^−^ are positive attributes when considering fluorescence imaging applications. An ADP-selective probe should also maintain stable luminescence at a pH around 7 to ensure accurate and reliable results under physiological conditions. Accordingly, the pH sensitivity of [Eu.ADPGlow]^−^ was evaluated by luminescence spectroscopy. We found that the emission intensity and spectral shape of the Eu(iii) complex remained essentially unchanged between pH 4 and 7.5 (Fig. S9†), indicating that the luminescence should not be affected by pH fluctuations within the range of normal lysosomal or cytosolic pH, estimated to be 4.4 and 7.3, respectively.^48,49^ As the pH is raised from 8 to 10 the emission intensity increases, indicating binding of hydroxide to the Eu(iii) metal centre. By fitting the change in emission intensity as a function of pH a p*K*_a_ value was estimated to be 8.58 ± 0.03. However, this increase in emission is not a concern, as pH values above 8 are not typically encountered in healthy cells. Complexes [Eu.6Ph]^+^ and [Eu.6PhOMe]^+^, which lack water-solubilising groups, were not considered further as potential probes due to their poor solubility in aqueous solution, leading to time-dependent decreases in emission over a 90 minutes incubation period (Fig. S10†).

### X-ray crystallography

**Fig. 3:**
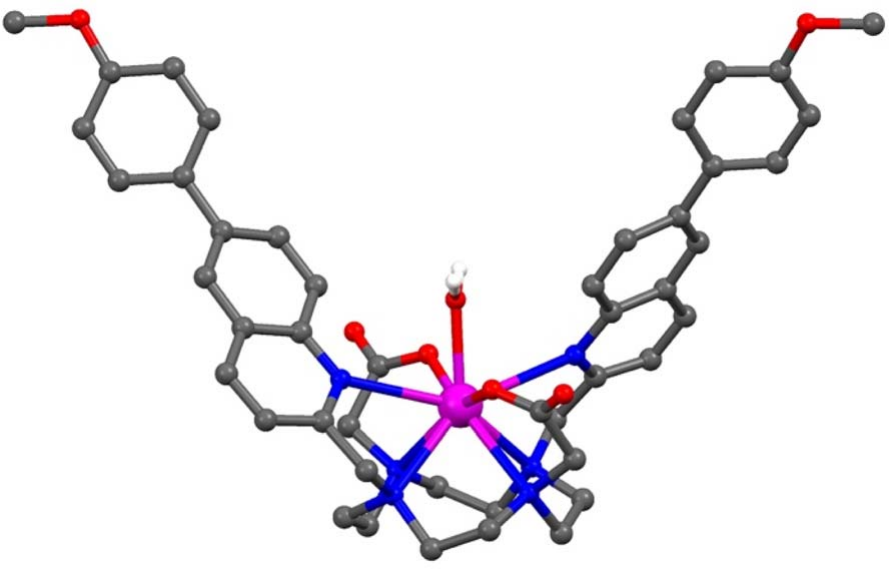
Single crystal X-ray structure of [Gd.6PhOMe]^+^ viewed perpendicular to the *a* axis, showing the ADP binding site in which a water molecule sits. Atom colours: Gd pink, C grey, N blue, O red, H white.

We investigated the structural characteristics of the complexes further using single crystal X-ray diffraction. Despite multiple attempts to grow single crystals of the water soluble complex [Eu.ADPGlow]^−^ and the corresponding ADP adduct, these could not be obtained. However, crystals of the structurally related Gd(iii) complex [Gd.6PhOMe]^+^ were obtained after slow evaporation from methanol and water. The Gd(iii) complex crystallised in the monoclinic space group *P*2_1_/*c*, with the asymmetric unit consisting of the complex, a non-coordinating triflate anion, and eleven water molecules, one of which is bound to the Gd(iii) ion. The ligand coordinates to the Gd(iii) as expected (Fig. 3), through the four nitrogen atoms of the cyclen ring (Gd–N 2.603(15)–2.699(15) Å) and the four pendant arms, including two acetate oxygen atoms (Gd–O 2.338(5) Å and 2.351(6) Å) and the two nitrogen atoms of the quinoline rings (Gd–N 2.853(6) Å and 2.889(6) Å). The conjugated quinoline arms are oriented on the same face of the macrocycle (which is disordered over two sites, with the largest occupancy being 70%) but in opposite directions, creating a central binding site in which a water molecule sits (Gd–O 2.369(6) Å). This coordination leads to a twisted square antiprismatic (TSAP) geometry of the major macrocyclic component, with a twisting angle of approximately 26° between the square defined by the cyclen ring nitrogen atoms and the square coordinating the oxygen atoms of the two carboxylate arms and the two coordinated nitrogen atoms of the quinoline rings (Fig. S11†). The methoxy-phenyl rings display twists co-planar to the quinoline rings (33.3(2)° and 33.6(2)°); with one ring involved in weak intermolecular π–π stacking interactions with the quinoline ring of a neighbouring complex (Fig. S12†).

### Anion binding studies at physiological pH

**Fig. 4:**
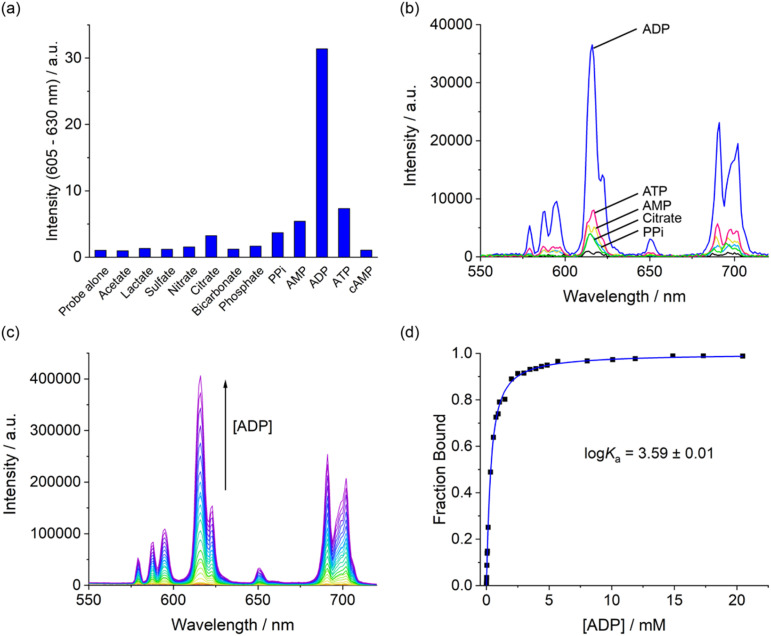
(a) Bar chart displaying emission enhancement of the Δ*J* = 2 (605–630 nm) band of [Eu.ADPGlow]^−^ with selected anions (1 mM each); (b) emission spectra of [Eu.ADPGlow]^−^ alone and in the presence of selected anions adenosine triphosphate (ATP), adenosine diphosphate (ADP), adenosine monophosphate (AMP), pyrophosphate (PPi), and citrate (1 mM each); (c) variation in emission spectra of [Eu.ADPGlow]^−^ upon incremental addition of ADP; (d) plot of fraction bound (determined from Δ*J* = 2/Δ*J* = 1 intensity ratio) *versus* ADP concentration, showing the fit to a 1 : 1 binding isotherm. Measured in 10 mM HEPES at pH 7.0 and 295 K, *λ*_ex_ = 337 nm.

The anion binding selectivity of complex [Eu.ADPGlow]^−^ was examined by measuring the emission spectra in the presence of different biologically relevant anions in 10 mM HEPES at pH 7.0. Addition of 1 mM ADP to [Eu.ADPGlow]^−^ caused a pronounced luminescence ‘switch on’, with a 33-fold emission enhancement (Fig. 4a). This is accompanied by changes in spectral shape (Fig. 4b and S13†), involving a large increase in the hypersensitive Δ*J* = 2 band centred around 615 nm indicating binding of ADP and displacement of the quenching water molecule. In contrast, very small (<2-fold) increases in emission were seen upon addition of ATP, PPi, AMP, and citrate, and virtually no response was detected for phosphate, bicarbonate, chloride, sulphate, lactate, acetate and nitrate. Given the scarcity of chemical or biological probes that recognise ADP over ATP (or PPi) in the literature, this result was unprecedented. The remarkable 33-fold enhancement observed for ADP with [Eu.ADPGlow]^−^, compared with the minimal 2-fold increase for ATP, demonstrates a level of sensing selectivity that, to the best of our knowledge, is unrivalled.^4,12,13,16^ Furthermore, the weak interaction of bicarbonate and phosphate is a particular advantage, as these abundant biological anions are well known to increase the emission of many reported lanthanide-based probes.^20,50^

The Eu(iii) probe demonstrates a notable ability to distinguish between nucleobases, with the purine diphosphates ADP and GDP showing significantly stronger (2–3 times larger) emission enhancements compared with the pyrimidines CDP and UDP (Fig. S14†). None of the nucleotide triphosphates (ATP, GTP, CTP, and UTP) produced more than an 8-fold enhancement, and negligible changes in emission were observed for all four nucleotide monophosphates—AMP, GMP, CMP, and UMP. The ability of [Eu.ADPGlow]^−^ to selectively recognise purine diphosphates suggests that secondary interactions within the host–guest structure are occurring, potentially involving π–π stacking between the purine base and one of the conjugated pendant arms. This is further supported by the minimal 4-fold increase in emission caused by pyrophosphate, which lacks a nucleobase (Fig. 4).

The ADP-induced emission enhancement of [Eu.ADPGlow]^−^ is accompanied by changes in fine structure of the Δ*J* = 1 band that, reveals information about the local symmetry around the europium ion (Fig. S15†).^51^ In this case ADP binding induces a change in crystal field around the Eu(iii) centre, involving a change in sign and magnitude of the parameter, *B*_0_^2^. This could be due to a change in conformation of the predominant species in solution, potentially from a twisted square antiprismatic geometry (TSAP) for the unbound complex to a square antiprismatic (SAP) structure when ADP is bound. However, unambiguous interpretation of these spectroscopic features remains challenging.^52,53^ Emission lifetimes recorded in the presence of ADP revealed that *q* = 0 (Table 2), consistent with displacement of a coordinated water molecule. In the presence of ATP and AMP, *q* values of 0.3 and 0.5 were found respectively, indicating partial hydration consistent with much smaller increases in emission intensity observed for these anions. In the presence of PPi and citrate, *q* = 1 indicating only weak interactions with these anions in water.

**Table 2:** Apparent binding constants for [Eu.ADPGlow]^−^ and selected anions and lifetime values for [Eu.ADPGlow]^−^ alone and in the presence of selected anions (1 mM) measured in 10 mM HEPES at pH 7.0^a^

Complex	Anion	log *K*_a_	*τ*_H_2_O_/ms	*τ*_D_2_O_/ms	*q*
[Eu.ADPGlow]^−^	None	—	0.021 ± 0.001	0.036 ± 0.001	—
	AMP	2.85 ± 0.05	0.33 ± 0.07	0.45 ± 0.15	0.6 ± 0.1
	ADP	3.59 ± 0.01	0.94 ± 0.04	1.35 ± 0.04	0.1 ± 0.0
	ATP	3.05 ± 0.03	0.64 ± 0.04	0.95 ± 0.02	0.3 ± 0.1
	PPi	2.91 ± 0.07	0.41 ± 0.01	0.69 ± 0.12	0.8 ± 0.4
	Citrate	2.55 ± 0.14	0.41 ± 0.02	0.70 ± 0.01	0.9 ± 0.2

a Experiments were completed in duplicate and error values determined through the averages ± standard deviation.

Apparent binding constants between [Eu.ADPGlow]^−^ and those anions that induced an observable emission change were determined by plotting the change in the intensity ratio of the Δ*J* = 2/Δ*J* = 1 emission bands (605–630/580–600 nm) as a function of guest concentration, followed by curve fitting based on a 1 : 1 binding model (Table 2 and Fig. S16–20†). The Eu(iii) complex showed the strongest binding to ADP (log *K*_a_ = 3.59 ± 0.01), approximately 5 times stronger than ATP and 7 times stronger than pyrophosphate. AMP and citrate exhibited weak binding, while phosphate induced only a 10% change in emission, even at high anion concentrations up to 20 mM (Fig. S21†), preventing reliable determination of a binding constant. Thus, the binding selectivity of [Eu.ADPGlow]^−^ follows the order ADP > ATP > PPi ∼ AMP, which is distinctly different to that observed for most nucleoside phosphate receptors^4,5,8^ where binding is driven predominantly by electrostatic interactions and thus favours ATP and PPi over ADP and AMP.

The affinity of [Eu.ADPGlow]^−^ for ADP was an order of magnitude lower than that of our previously studied cationic complex [Eu.1]^+^ under identical conditions (log *K*_a_ for ADP = 4.6 ± 0.1). Assuming a similar bidentate binding interaction with the diphosphate group of ADP,^26,27^ the reduced affinity of [Eu.ADPGlow]^−^ can be ascribed to its overall negative charge and the absence of stabilising hydrogen bond donors in the ADP binding site. These structural differences are important, as they not only diminish the binding strength but also lead to a significant decrease in emission response toward ATP and PPi (Fig. S22†), thus enhancing discrimination for ADP. This is largely ascribed to increased electrostatic repulsion between ATP and [Eu.ADPGlow]^−^, combined with a greater degree of luminescence quenching from water molecules in the second coordination sphere of the ATP-receptor complex. Ultimately, these structural features of [Eu.ADPGlow]^−^ afford it far greater sensing selectivity for ADP over ATP, PPi, AMP, and citrate, representing a major improvement in discrimination compared with the previous lead compound, [Eu.1]^+^ (Fig. S22†).

To gain further insight into the binding mode of ADP to [Eu.ADPGlow]^−^, we turned to ^1^H and ^31^P NMR spectroscopy. As discussed earlier, the ^1^H NMR spectrum of [Eu.ADPGlow]^−^ at room temperature was complicated by the presence of multiple conformations, evident from four sets of signals with significant exchange broadening (Fig. S3c†). Upon addition of ADP (in 1 : 1 D_2_O/CD_3_OD, pD 7.0, Fig. S24†), the original signals disappeared, coinciding with the emergence of a new set of resonances corresponding to the ADP-bound species. These new resonances displayed more pronounced line broadening, attributed to either increased conformational flexibility in the host–guest complex or the presence of more than one host–guest species in solution.

In the ^31^P NMR spectrum, four signals were observed for [Eu.ADPGlow]^−^ in the presence of ADP (Fig. S25†). The two ^31^P signals at −79 and −125 ppm, corresponding to bound ADP, were significantly broader than the free ADP signals at −4.8 and −9.1 ppm, further suggesting exchange between different binding modes for ADP. This observation is consistent with previous NMR analysis of the structurally related [Eu.1]^+^, as well as recent EXAFS and EPR studies, which established that ADP binds *via* both bidentate and monodentate modes, with a preference for the former. Further interpretation of the NMR data for [Eu.ADPGlow]^−^ is not straightforward, prompting us to seek additional insight through DFT calculations of the host–guest species.

### DFT computations of host–guest binding

**Fig. 5:**
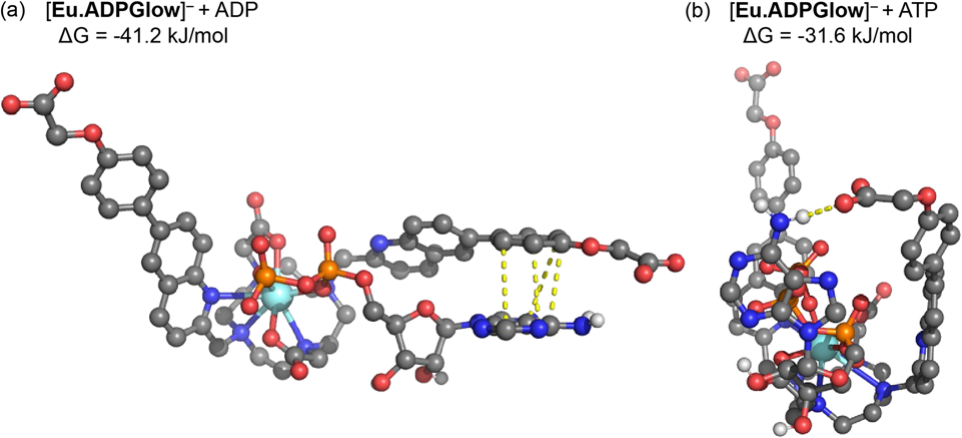
DFT optimised structures and binding free energies (Δ*G*) of [Eu.ADPGlow]^−^ bound to (a) ADP and (b) ATP. In part (a) dashed bonds indicate π–π stacking (3.45–3.92 Å) between the adenine base of ADP and the conjugated phenyl group of [Eu.ADPGlow]^−^. In part (b) a dashed bond represents a hydrogen bond (1.97 Å) between the peripheral carboxylate of [Eu.ADPGlow]^−^ and the adenine base of ATP.

The crystal structure of the Gd(iii) complex (Fig. 3) was used as a starting point to model the interactions of [Eu.ADPGlow]^−^ with ADP and ATP. Computations were performed at the r^2^SCAN-3c level of theory, along with the SMD solvation model for water, to model anion binding geometries and interaction energies.^54–56^ We substitute a Y(iii) ion for Eu(iii) to avoid complications arising from the f-electrons, noting that Y(iii) complexes have been shown previously as suitable models for their Eu(iii) analogues, based on the ionic radii varying by only 0.05 Å.^57,58^Fig. 5 presents the structures for ADP and ATP binding with [Eu.ADPGlow]^−^; further details are presented in Fig. S26.† In line with previous studies on anion adducts of the structurally related complex [Eu.1]^+^, we use bidentate binding modes wherein ADP binds predominantly *via* its α and β phosphates, while ATP binds *via* its α and γ phosphates.^26,27^ Binding of ADP (Fig. 5a) causes one of the quinoline arms to become detached from the europium(iii) ion, whereas the remaining ligand binding sites (macrocyclic nitrogens, second quinoline nitrogen and carboxylate oxygen atoms) remain intact. Viewing the dissociated quinoline arm, we notice pronounced π–π stacking interactions between the adenine base of ADP, highlighted *via* yellow dashed lines. Crucially, stacking occurs through the terminal phenyl group of the dissociated arm, rather than the quinoline unit. This interaction is not possible with our previously reported complexes, such as [Eu.1]^+^, which lack the conjugated phenyl group. Consequently, it is reasonable to associate the enhanced ADP-sensing performance of [Eu.ADPGlow]^−^ to the presence and influence of this conjugated phenyl group.

The binding mode observed for ATP (Fig. 5b) is distinctly different to that of ADP. Binding of [Eu.ADPGlow]^−^ to ATP not only dissociates one of the quinoline arms but also weakens binding to the macrocyclic nitrogens (Fig. S26†), highlighting the increased steric demands imposed by the three phosphate groups on the binding site of [Eu.ADPGlow]^−^. Due to this different binding geometry, we do not find stacking interactions between adenine and the quinoline arm, with their interactions instead stabilised by a hydrogen bond.

The computed binding free energies for PPi, ADP and ATP are 28.9, 41.2 and 31.6 kJ mol^−1^, respectively (see Table S2†), clearly highlighting the increased affinity for ADP. Experimental binding free energies for these three species determined from the apparent binding constants (Table 2) are 26.6, 30.4 and 27.4 kJ mol^−1^, respectively, showing that the models provide reasonable estimates, though the computed binding energies are somewhat overestimated. This may arise from factors such as ionic strength, speciation of the anion, explicit hydration effects not covered by the continuum model, and thermostatistical corrections to the binding free energies. Efforts to develop more accurate methods to account for these factors are currently underway and will be reported in due course.

### Time-resolved detection of ADP in simulated physiological media

Our ultimate goal is to utilise [Eu.ADPGlow]^−^ in the analysis of real biological samples, which present far greater complexity than the buffered aqueous solutions explored thus far. Towards this goal, we conducted competitive titration experiments to assess the performance of [Eu.ADPGlow]^−^ in more biologically relevant conditions. First, we confirmed the ability of [Eu.ADPGlow]^−^ to reliably recognise ADP in the presence of physiological concentrations of human serum albumin (HSA, 0.4 mM) and bicarbonate (27 mM). At this point, we utilised the long-lived emission of [Eu.ADPGlow]^−^ to record time-resolved luminescence measurements (integration time = 60–400 μs), gating out short-lived fluorescence from biological fluorophores (*e.g.* tryptophan and tyrosine residues). Addition of 0.4 mM HSA to [Eu.ADPGlow]^−^ caused a minor (12-fold) increase in time-resolved Eu(iii) emission intensity and no change in spectral form, whereas 27 mM bicarbonate caused a negligible increase in emission (Fig. S27†). Crucially, neither HSA protein nor bicarbonate significantly affected the probe's selective emission response towards ADP (Fig. S27†), underscoring its potential use in more complex biological environments.

Encouraged by these results, we wished to establish whether the probe could detect ADP against a background of high ATP concentration. A competitive titration experiment involving incremental addition of ADP over a physiologically relevant range (0–1.2 mM) caused a 5-fold increase in time-resolved emission within the Δ*J* = 2 band (Fig. S28†), confirming that millimolar levels of ATP have little to no impact on the probe's ability to detect ADP.

**Fig. 6:**
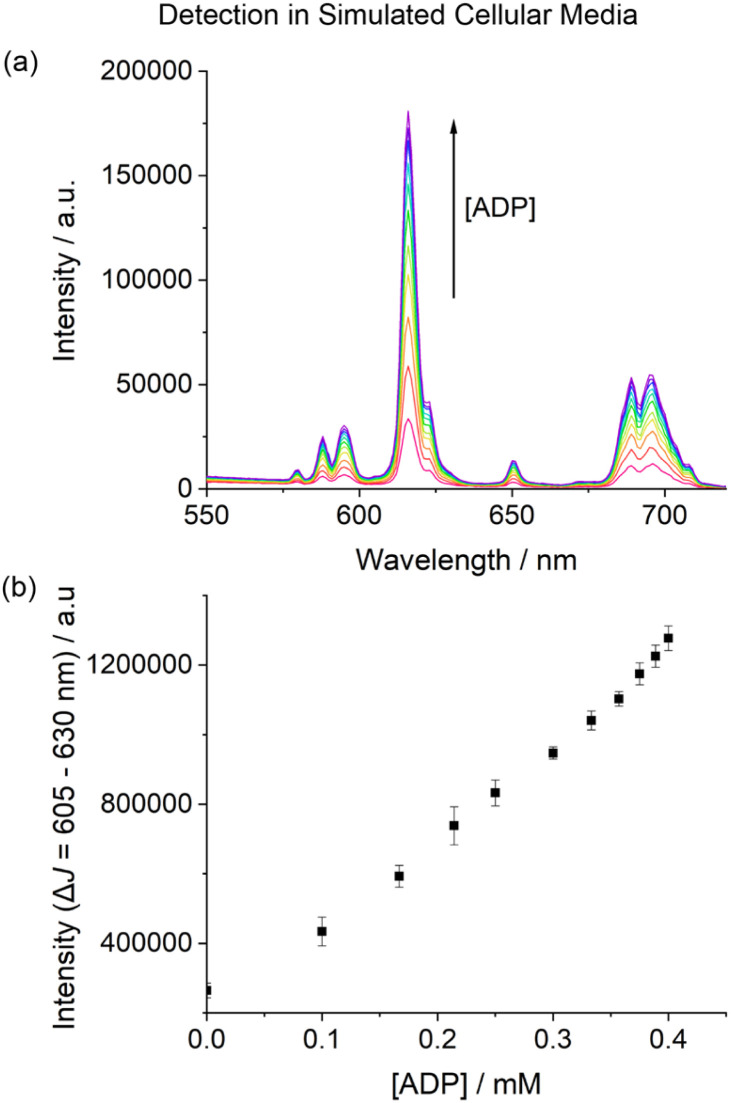
Selective detection of ADP in a simulated cellular media based on a Krebs saline solution, containing ATP (1 mM), KCl (145 mM), NaCl (5 mM), CaCl_2_ (2.5 mM), MgCl_2_ (1.5 mM), NaHCO_3_ (27 mM), Na_2_SO_4_ (0.5 mM), sodium lactate (1.0 mM), sodium citrate (0.15 mM), glucose (5.5 mM) and HSA (0.4 mM) in 10 mM HEPES buffer (pH 7.0). (a) Change in emission spectra of [Eu.ADPGlow]^−^ (10 μM) upon addition of ADP in simulated cellular media; (b) plot of the emission intensity of the Δ*J* = 2 region (605–630 nm) showing a 5-fold linear increase in emission upon adding 10–400 μM ADP. *λ*_ex_ = 337 nm, 295 K.

Next, we examined the performance of [Eu.ADPGlow]^−^ in an aqueous medium designed to simulate the complex ionic environment within cells. The media is based on a modified Krebs saline solution routinely used for cell culture experiments, containing ATP (1 mM), NaCl (145 mM), KCl (5 mM), CaCl_2_ (2.5 mM), MgCl_2_ (1.5 mM), NaHCO_3_ (27 mM), Na_2_SO_4_ (0.5 mM), sodium lactate (1.0 mM), sodium citrate (0.15 mM), glucose (5.5 mM) and HSA (0.4 mM), buffered in 10 mM HEPES at pH 7.0. Remarkably, titration of ADP to [Eu.ADPGlow]^−^ under these conditions induced a reproducible 5-fold increase in time-resolved emission within the Δ*J* = 2 band (Fig. 6). Importantly, the emission increase was approximately linear over the physiologically relevant ADP concentration range of 10–400 μM (Fig. 6b).^59^ These promising results highlight the potential of [Eu.ADPGlow]^−^ as a sensitive probe for detecting cellular ADP levels, prompting us to undertake cellular imaging experiments using fluorescence microscopy.

### Cellular uptake and localisation studies

**Fig. 7:**
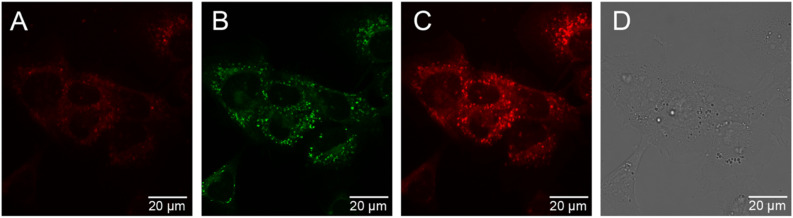
NIH-3T3 cellular images of [Eu.ADPGlow]^−^ (10 μM, 2 hours incubation) with LysoTracker Green™ (LTG). (A) [Eu.ADPGlow]^−^ (*λ*_ex_ = 355 nm, *λ*_em_ = 600–735 nm); (B) LTG highlighting predominantly lysosomal localisation (*λ*_ex_ = 488 nm, *λ*_em_ = 500–600 nm); (C) [Eu.ADPGlow]^−^ and NIH-3T3 autofluorescence (*λ*_ex_ = 355 nm, *λ*_em_ = 500–735 nm); (D) corresponding transmission image. Scale bars = 20 μm.

The cellular uptake behaviour of [Eu.ADPGlow]^−^ was investigated in NIH-3T3 cells using epifluorescence and laser scanning confocal microscopy.^60^ Following incubation of [Eu.ADPGlow]^−^ (10 μM) in NIH-3T3 cells for 2 hours, microscopy images revealed uptake of the Eu(iii) complex with localization predominantly within the lysosomes (*λ*_ex_ 355 nm, *λ*_em_ 605–720 nm), verified by co-localization studies using LysoTracker™ Green (*λ*_ex_ 488 nm, *λ*_em_ 500–600 nm, 5 minutes loading) but with mitochondrial staining also evident (Pearson's correlation coefficient for lysosomal co-staining, *P* = 0.58) (Fig. 7). We were able to incubate the cells with the Eu(iii) compound for extended time periods (up to 24 hours); the brightness of the observed images did not vary significantly between the 2 hours and the 24 hours incubation and the cells appeared to be non-toxic at the tested 10 μM loading concentration. See the ESI† for a detailed analysis of probe brightness within cells. Analysis of ICP-MS data revealed that for 9 × 10^5^ NIH-3T3 cells incubated with [Eu.ADPGlow]^−^ (10 μM) for 2 hours, a given cell contained 2.25 μM (±5%) of Eu(iii) ion, which indicates minimal accumulation of [Eu.ADPGlow]^−^ within the cells during the incubation period, contributing to the low overall europium(iii) emission observed within the cells. The low emission of [Eu.ADPGlow]^−^ in the absence of ADP, combined with its excellent selectivity for ADP, indicates that [Eu.ADPGlow]^−^ could effectively visualise ADP changes in living cells in response to physiological perturbations. We plan to improve these features further by modification of the antenna of the [Eu.ADPGlow]^−^ to increase its brightness, cellular uptake and localisation profile.^33^

## Conclusions

In summary, we have developed a novel water-soluble europium(iii) probe, [Eu.ADPGlow]^−^, which exhibits very high selectivity and sensitivity for ADP, making it a valuable tool for monitoring dynamic biological processes involving this anion. The probe incorporates π-conjugated quinolyl-phenoxyacetate antennae, ensuring efficient excitation at 355 nm, high water solubility, and resistance to non-specific binding to albumin protein. The probe's affinity for ADP (log *K*_a_ = 3.59 ± 0.01) results in a pronounced luminescence ‘switch on’ with negligible interference from ATP, pyrophosphate, phosphate, bicarbonate, and many other biological anions. This selective recognition allows for time-resolved detection of ADP across the physiologically relevant concentration range (10–400 μM), even in the presence of millimolar levels of ATP.

Photophysical studies revealed that the probe's emission intensity is stable across physiological pH ranges and its emission lifetime is significantly enhanced upon ADP binding, indicating effective displacement of coordinated water. The probe stands out as one of rare examples demonstrating selectivity for ADP over ATP and pyrophosphate, a critical feature for accurate detection in complex biological environments.

The Eu(iii) probe can permeate mammalian cells, showing a broad distribution within mitochondria and lysosomes. This, coupled with its low background emission (*i.e.*, minimal emission signal without ADP) makes [Eu.ADPGlow]^−^ a promising candidate for real-time visualization of dynamic cellular ADP levels. Our findings underscore the potential of [Eu.ADPGlow]^−^ to enhance the understanding of the role of ADP in various biochemical and cellular processes, whilst offering a convenient luminescence tool for monitoring kinase and ATPase activities in real-time. Future work will focus on further optimizing the probe for cellular applications, including introduction of peripheral substituents to promote cellular uptake and subcellular localisation.^33^ Overall, we anticipate that the broad applicability and excellent selectivity of [Eu.ADPGlow]^−^ will make it an exceptionally popular tool.

## Data availability

The data supporting this article have been included as part of the ESI,† including synthesis and characterisation of ligands and corresponding Eu(iii)/Gd(iii) complexes, UV-vis and emission spectral data, anion binding studies, X-ray crystallography (PDF) and Single Crystal X-ray Structure (cif.) data.

## Author contributions

The manuscript was written by S. J. B. and S. E. B, with contributions from each author. S. E. B. carried out the synthesis and characterisation, photophysical measurements and anion binding studies. U. R. performed the DFT computational experiments with support from F. P., and P. S. undertook the cell imaging experiments with support from R. P.

## Conflicts of interest

There are no conflicts to declare.

## Supplementary Material

SC-OLF-D4SC07188C-s001

SC-OLF-D4SC07188C-s002
