# Supplementary material for: A switch-on luminescent europium(iii) probe for selective and time-resolved detection of adenosine diphosphate (ADP)
Source: Chem Sci. 2025 Feb 19;16(13):5602–12. doi: 10.1039/d4sc07188c (PMC11868914; doi:10.1039/d4sc07188c)
Supplement: SC-016-D4SC07188C-s001 [file SC-016-D4SC07188C-s001.pdf]

Supplementary Information (SI) for Chemical Science.
This journal is © The Royal Society of Chemistry 2025

Electronic Supporting Information

A Switch-On Luminescent Europium(III) Probe for Selective and Time-Resolved Detection of Adenosine Diphosphate (ADP)

Samantha E. Bodman,[a] Patrycja Stachelek,[b] Umatur Rehman,[a] Felix Plasser,[a] Robert Pal[b] and Stephen J Butler[a]*

[a] Department of Chemistry, Loughborough University, Epinal Way, Loughborough, LE11 3TU, UK.
[b] Department of Chemistry, Durham University, South Road, Durham DH1 3LE, UK.
*Correspondence: S.J.Butler@lboro.ac.uk

Contents

1. Materials and Methods S2
2. Characterisation Eu(III) and Gd(III) complexes S6
   HPLC traces [**Eu.ADPGlow**]$^-$ and [**Gd.ADPGlow**]$^-$
   ^{1}H NMR spectra of [**Eu.6Ph**]$^+$, [**Eu.6PhOMe**]$^+$, and [**Eu.ADPGlow**]$^-$
   Mass spectrometry of [**Eu.6Ph**]$^+$, [**Eu.6PhOMe**]$^+$, and [**Eu.ADPGlow**]$^-$
3. Synthesis and characterisation of ligands and corresponding Ln(III) complexes S9
4. Photophysical measurements of Eu(III) and Gd(III) complexes S54
5. X-ray Crystallography of [**Gd.6PhOMe**]$^+$ S66
6. References S75

1. Materials and Methods

General Considerations

Reagent grade chemicals, including the anhydrous solvents, were purchased from Sigma Aldrich and Fluorochem and used without further purification.

Nuclear Magnetic Resonance

^{1}H, ^{13}C, ^{31}P, COSY, HMQC and HMBC NMR spectra were recorded in the stated deuterated solvent on a JEOL ECS-400 or 500 spectrometers (^{1}H at 400 MHz, ^{13}C at 101 MHz; ^{1}H at 500 MHz, ^{13}C at 126 MHz, ^{31}P at 202 MHz, respectively), at 298 K. Chemical shifts are expressed in ppm and are adjusted to the chemical shift of the residual NMR solvent resonances ($CDCl_3$: ^{1}H δ = 7.26 ppm, ^{13}C δ = 77.16 ppm, CD_3OD: ^{1}H δ = 3.31 ppm, ^{13}C δ = 49.00 ppm or DMSO-d_6: ^{1}H δ = 2.50 ppm, ^{13}C δ = 39.52 ppm). The coupling constants are expressed in Hz.

Liquid Chromatography Mass Spectrometry

Liquid Chromatography Electrospray Mass spectra were recorded on a Shimadzu Prominence LC system with a Shimadzu SPDM20A Photodiode Array Detector, a Shimazu CTO-20A column oven, Shimadzu SIL-20A autosampler and a Shimadzu LCMS 20 mass spectrometer controlled using LabSolutions software. The system operates in positive ion mode, with acetonitrile as the carrier solvent. The flow rate was maintained at 0.7 mL/min over a gradient of 5 to 95% acetonitrile in water (0.1% formic acid) for 10 minutes. High resolution mass spectra were recorded using a Thermofisher Q-Exactive orbitrap mass spectrometer.

Column Chromatography

Column chromatography was performed using flash silica gel 60 (particle size 40–63 microns) purchased from Apollo Scientific. Thin layer chromatography (TLC) was performed on aluminium sheet silica gel plates with 0.2 mm thick silica gel 60 F254 using the stated mobile phase.

High Performance Liquid Chromatography

Preparative RP-HPLC was performed using a Waters 2489 UV/Visible detector performed at 254 nm, a Waters 1525 Binary HPLC pump controlled by the Waters Breeze 2 HPLC system software. Separation was achieved using a semi-preparative XBridge C18 (5 μm OBD 19 × 100 mm) column at a flow rate maintained at 17 mL/min). A solvent system composed of either water (0.1% formic acid)/methanol (0.1% formic acid) or water (50 mM NH_4HCO_3)/acetonitrile was used over the stated linear gradient (usually 0 to 100% organic solvent over 17 - 25 mins). Analytical RP-HPLC was performed using a XBridge C18 5 μm 4.6 × 100 mm at a flow rate maintained at 2.0 mL/min using the same gradients and solvents.

Luminescence Experiments

Luminescence spectra were recorded on a Camlin Photonics luminescence spectrometer with FluoroSENS version 3.4.7.2024 software. Emission spectra were obtained using a 40 μL or 100 μL Hellma Analytics quartz cuvettes. Excitation light was set at the absorption maxima and emission recorded in the range 350–720 nm using an integration time of 0.5 seconds, increment of 1.0 nm, excitation slit of 0.2 nm and emission slit of 0.5 nm. Quantum yields were measured using quinine sulfate in 0.05 M H_2SO_4 as a standard (Φ_{em}= 0.60, λ_{ex} =

350 nm).[1] Emission lifetime measurements were performed on the FluoroSENS instrument. Measurements were taken of 1 mL of 0.1 absorbance samples of Eu(III) complexes in 10 mM HEPES at pH 7.0, unless stated otherwise. Measurements were obtained by indirect excitation of the Eu(III) ion via the quinoline antennae using a short pulse of light at 328 nm ([**Eu.6Ph**]$^+$), 340 nm ([**Eu.6PhOMe**]$^+$), or 337 nm ([**Eu.ADPGlow**]$^-$) followed by monitoring the integrated intensity of the light emitted at 615 nm, with 500 data points collected over a 10 millisecond time period. The decay curves were plotted in Origin Labs 2019 version 9.6.0.172, and fitted to the equation:

$$\mathrm{I} = \mathrm{A0} + \mathrm{A1}\, e^{-kt}$$

where I is the intensity at time, t, following excitation, A_0 is the intensity when decay has ceased, A_1 is the pre-exponential factor and k is the rate constant for the depopulation of the excited state.

The hydration state, q, of the Eu(III) complexes was determined using the modified Horrocks equation:[2]

$$\mathrm{q\,(Eu)} = 1.2\,(\frac{1}{\mathrm{tH_2O}} - \frac{1}{\mathrm{tD_2O}} - 0.25 - 0.075\mathrm{n})$$

where t_{H2O} and t_{D2O} are the emission lifetime times in water and D_2O, respectively, and n is the number of carbonyl-bound amide NH groups.

The methanol hydration state, m, of the Eu(III) complexes was determined using:

$$\mathrm{m\,(Eu)} = 2.1\,(\frac{1}{\mathrm{tCH_3OH}} - \frac{1}{\mathrm{tCD_3OD}})$$

where t_{CH3OH} and t_{CD3OD} are the emission lifetime times in methanol and methanol-d_4, respectively.

Anion Binding Titrations

Anion binding titrations were carried out in duplicate in degassed 10 mM HEPES buffer at pH 7.0 for complexes [**Eu.ADPGlow**]$^-$. Stock solutions of anions (e.g. inorganic phosphate, ADP) containing Eu(III) complex (0.1 Abs) were made up at 0.4, 4 and 40 mM anion. The appropriate anion stock solution was added incrementally to 100 µL of Eu(III) complex (0.1 Abs) and the emission spectrum was recorded after each addition. The ratio of emission bands 605 – 630 nm/ 585 – 600 nm (Δ*J* = 2 / Δ*J* = 1) was plotted as a function of anion concentration. The data was analysed using a nonlinear least-squares curve fitting procedure, based on a 1:1 binding model described by the equation:

$$\mathrm{FB} = \frac{\frac{1}{\mathrm{K_a}} + [\mathrm{A}] + [\mathrm{Eu}] - \sqrt{(\frac{1}{\mathrm{K_a}} + [\mathrm{A}] + [\mathrm{Eu}])^2 + 4[\mathrm{A}][\mathrm{Eu}]}}{2[\mathrm{Eu}]}$$

where FB is the fraction bound, calculated by $(I-I_0)/(I_1-I_0)$ where I is the emission intensity at [A], I_0 is the initial emission intensity, and I_1 is the final emission intensity. [A] is the total concentration of anion in solution, [Eu] is the total concentration of Eu(III) complex, K_a is the apparent binding constant.

Triplet State Energy Measurements

Phosphorescence spectra were recorded using the FluoroSENS instrument. Measurements were taken of 0.2 absorbance samples of the Gd(III) complexes in diethyl ether/isopentane/ethanol (*v/v* 5:5:2) at 293 K and 77 K, unless otherwise stated. Spectra were obtained by excitation of the quinoline antennae at 328 nm (**Gd.6Ph**]$^+$), 342 nm ([**Gd.6PhOMe**]$^+$), or 337 nm ([**Gd.ADPGlow**]$^-$) followed by a time-resolved measurement of emission (from 60 µs to 400 µs), collecting data in 1 nm steps between 350–720 nm.

pH Titrations

A solution of Eu(III) complex (0.1 Abs) in water was adjusted to pH 11.0 by the addition of 1 M NaOH and an emission spectrum recorded. The pH was decreased slowly by 0.2 – 0.5 units by the addition of 1 M or 0.1 M HCl solution and an emission spectrum recorded at each pH. The ratio of emission bands 605–630 nm/ 585–600 nm ($\Delta J = 2$ / $\Delta J = 1$) was plotted as a function of pH and fitted to a sigmoidal curve using OriginLab 2019 to determine the pK_a value.

X-ray Crystallography

Single crystal X-ray diffraction experiments were performed by the UK National Crystallography Service on a Rigaku FRE+ diffractometer with HF Varimax confocal mirrors, an UG2 goniometer and HyPix 6000HE detector. The crystals were collected at 100(2) K. The structure was solved by direct methods using ShelXT[3] and refined with ShelXL[4] using a least squares method. Olex2 software[5] was used as the solution, refinement and analysis program.

Computational Details

All Density Functional Theory (DFT) calculations were carried using r^{2}SCAN-3c method[6] within Orca version 5.0.1[7]. For considering the effects of water as solvent, the solvation model based on density (SMD)[8] was employed. Europium(III) was replaced by yttrium(III) in all the calculations to avoid complications deriving from the 4f electrons of europium(III), following preliminary tests highlighting that this is a viable approach.[9,10] The reported binding free energies are derived from single point electronic structure computations in connection with SMD solvation free energies. The reaction considered for computing binding energies is as follows:

$$Eu—H_2O + Analyte \rightarrow Eu—Analyte + H_2O$$

Here Eu—H_2O and Eu—Analyte represent water-bound and analyte bound europium complexes respectively where the analyte can be pyrophosphate, ADP or ATP. Furthermore, the experimentally determined apparent binding constants are converted to the binding free energies by using the following set of equations:

$$\log K' = \log K_a + \log[\mathrm{H_2O}] \qquad (1)$$

$$\Delta^{\mathrm{e}}\mathrm{G} = -\mathrm{RT}\mathbf{ln}K' \qquad (2)$$

Here *K'* is the binding constant directly determined from the computed standard free binding energies (Δ^{e}G) and K_a is the measured apparent binding constant corrected for the molar concentration of water, i.e., [H_2O] = 55.6 mol/dm^3.

Cell Culture

A detailed investigation of the cellular behaviour of the Eu(III) complex was conducted using mouse skin fibroblasts (NIH-3T3) and human prostate adenocarcinoma (PC3) cell lines using fluorescence and laser scanning confocal microscopy. Cells were maintained in exponential growth as monolayers in F-12/DMEM (Dulbecco's Modified Eagle Medium) 1:1 that was supplemented with 10% foetal bovine serum (FBS). Cells were grown in 75 cm^2 plastic culture flasks, with no prior surface treatment. Cultures were incubated at 37 °C, 20% average humidity and 5% (v/v) CO_2. Cells were harvested by treatment with 0.25% (v/v) trypsin solution for 5 min at 37 °C. Cell suspensions were pelleted by centrifugation at 1000 rpm for 3 min and were re-suspended by repeated aspiration with a sterile plastic pipette. Microscopy Cells were seeded in 12-well plates on 13 mm 0.17 mm thick standard glass coverslips or un-treated iBibi 100 uL live cell channels and allowed to grow to 40% – 60% confluence, at 37 °C in 5% CO_2. At this stage, the medium was replaced, and cells were

treated with [**Eu.ADPGlow**]$^-$ and co-stains as appropriate. For imaging DMEM media (10% FBS) lacking phenol red was used from this point onwards. Following incubation, the coverslips were washed with phosphate-buffered saline (PBS; pH 7.4), mounted on slides and the edges sealed with colourless, quick-dry nail varnish to prevent drying out of the sample.

Cell toxicity measurements were run using a ChemoMetec A/S NucleoCounter3000-Flexicyte instrument with Via1-cassette cell viability cartridge (using the cell stain Acridine Orange for cell detection, and the nucleic acid stain DAPI for detecting non-viable cells). In cellular uptake studies, cells were seeded in 6-well plates and allowed to grow to 80–100% confluence, at 37 °C in 5% CO_2. At this stage, the medium was replaced with media containing [**Eu.ADPGlow**]$^-$ as detailed above and total cellular europium was determined using ICP-MS, inductively coupled plasma mass spectrometry by Dr. C. Ottley in the Department of Earth Sciences at Durham University.

Steady State Fluorescence Microscopy

Steady state fluorescence images were recorded using a PhMoNa[11] enhanced Leica SP5 II LSCM confocal microscope equipped with a HCX PL APO 63x/1.40 NA LambdaBlue Oil immersion objective. Data were collected using 5x digital magnification at 400 Hz/line scan speed (4 line average, bidirectional scanning) at 355 nm (3rd harmonic NdYAG laser) with 3 mW laser power. In order to achieve excitation with maximal probe emission, the microscope was equipped with a triple channel imaging detector, comprising two conventional PMT systems and a HyD hybrid avalanche photodiode detector. The latter part of the detection system, when operated in the BrightRed mode, is capable of improving imaging sensitivity by 25%, reducing signal to noise by a factor of 5. Frame size was determined at 2048 x 2048 pixel, with 0.6 airy disc unit determining the applied pinhole diameter rendering on voxel to be corresponding to 24.02 x 24.02 nm (frame size 49.16 x 49.16 μm) with a section thickness of 380 nm. A He, Ne or Ar ion laser was used when commercially available organelle-specific stains (e.g. LysoTrackerRedTM) were used to corroborate cellular compartmentalization.

2. Characterisation of Eu(III) and Gd(III) complexes

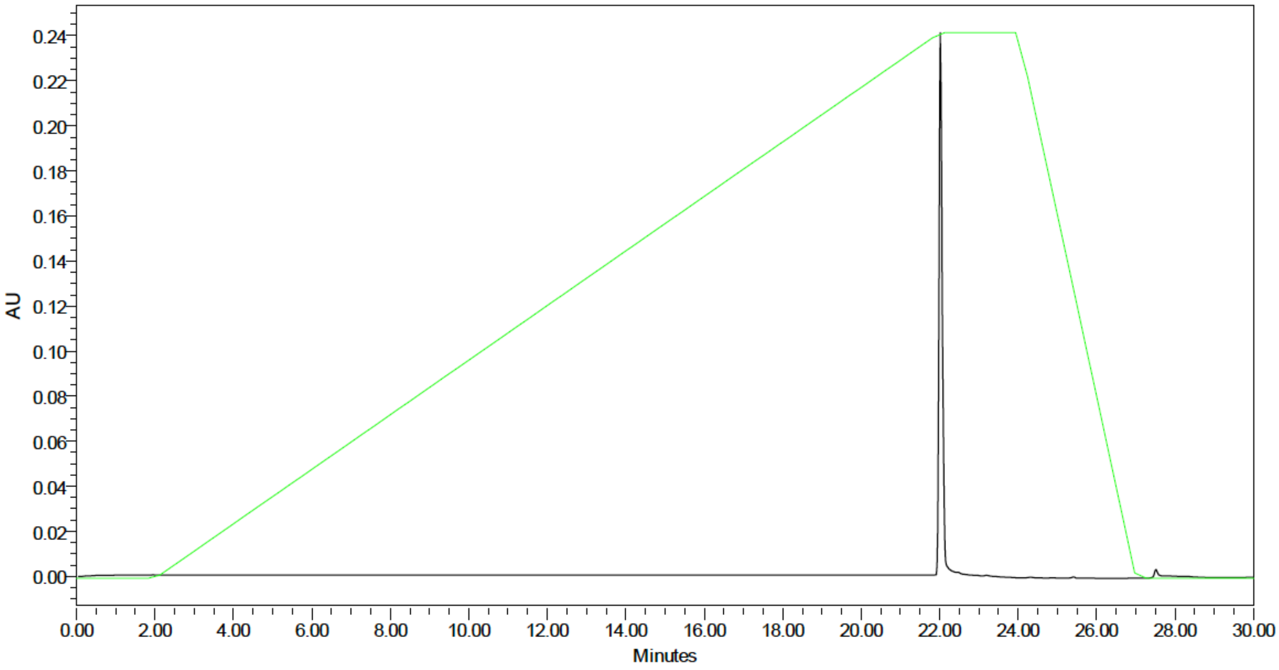

Figure S1. Analytical trace of complex [**Eu.ADPGlow**]$^{-}$. Conditions: RP-HPLC [gradient: 0 − 100% acetonitrile in 100 mM NH_4HCO_3 over 30 minutes, at 2 mL per minute; t_R = 22.00 min].

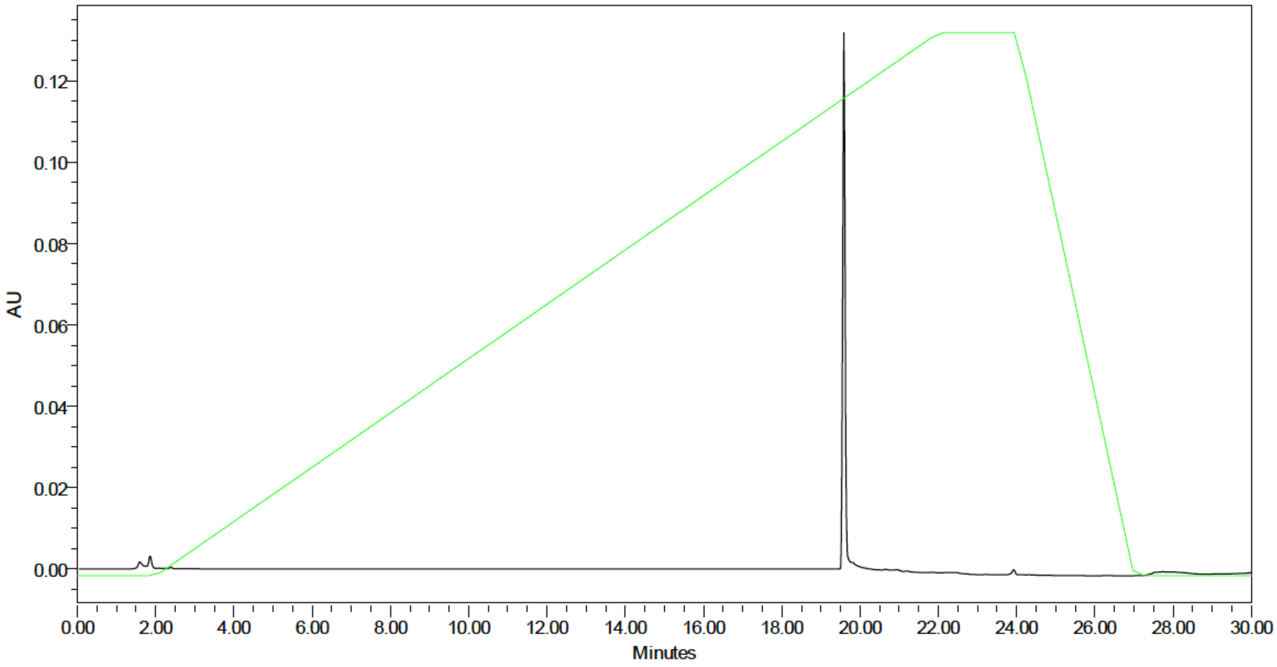

Figure S2. Analytical trace of complex [**Gd.ADPGlow**]$^{-}$. Conditions: RP-HPLC [gradient: 0 − 100% acetonitrile in 100 mM NH_4HCO_3 over 30 minutes, at 2 mL per minute; t_R = 19.58 min].

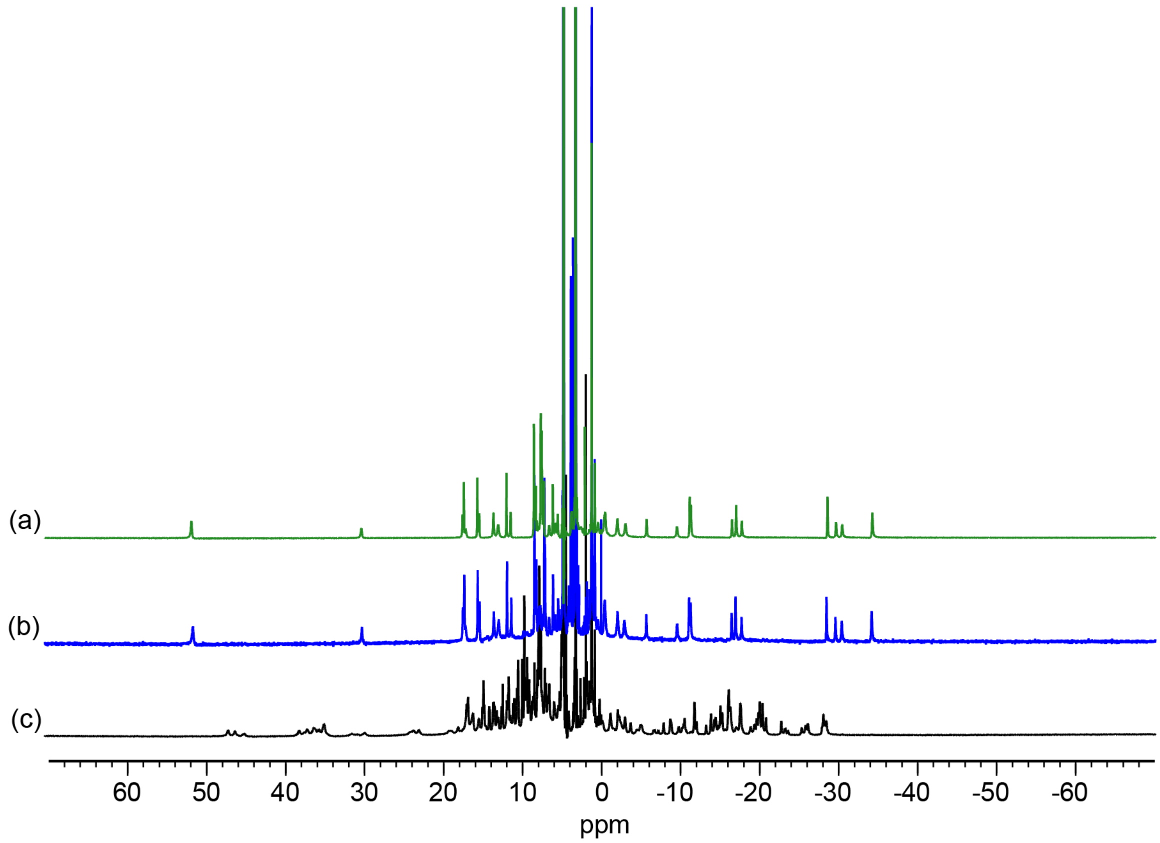

Figure S3. ^{1}H NMR spectra (500 MHz, CD_3OD) of (a) [**Eu.6Ph**]$^{+}$, (b) [**Eu.6PhOMe**]$^{+}$, and (c) [**Eu.ADPGlow**]$^{-}$ recorded at 298 K.

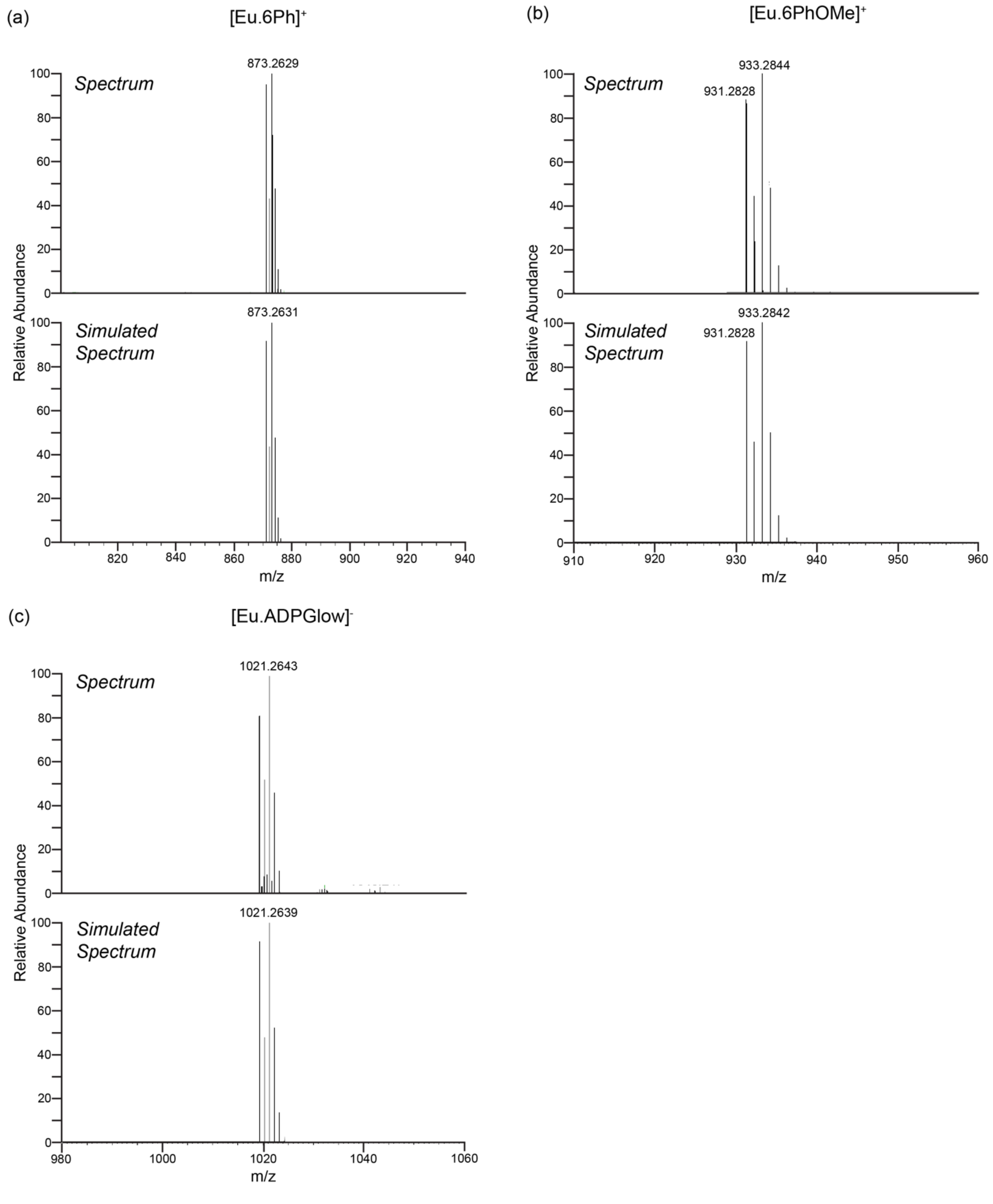

Figure S4. High resolution mass spectra of (a) [**Eu.6Ph**]$^+$, (b) [**Eu.6PhOMe**]$^+$, (c) [**Eu.ADPGlow**]$^-$ measured in methanol at 293 K.

3. Synthesis and characterisation of ligands and corresponding Ln(III) complexes

2-Methyl-6-phenylquinoline (**1a**)

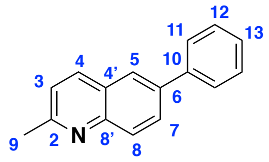

6-Bromoquinaldine (0.30 g, 2.70 mmol), phenyl boronic acid (0.20 g, 4.05 mmol) and potassium carbonate (0.60 g, 8.10 mmol) were added to an oven-dried Schlenk with a condenser attached under a nitrogen atmosphere. Anhydrous dioxane (20 mL) and oxygen-free water (3 mL) were added, followed by palladium-tetrakis(triphenylphosphine) (0.16 g, 0.14 mmol) and the reaction was heated to 60 °C for 16 hours. The reaction as cooled to room temperature, filtered through celite and the filtrate was removed under reduced pressure. The residue was dissolved in dichloromethane (50 mL) then washed with water (50 mL). The organic layer was separated, and the aqueous layer washed with dichloromethane (3 x 50 mL). The organic layers combined, washed with brine (100 mL), dried ($MgSO_4$) and solvent evaporated under reduced pressure. The product was obtained after column chromatography (silica gel; 5:95 ethyl acetate/hexane) to give the product as a white solid (0.548, 93%).

^{1}H NMR (500 MHz, $CDCl_3$): δ 8.10 – 8.08 (2H, m, H^4, H^8), 7.96 – 7.92 (2H, m, H^5, H^7), 7.72 – 7.71 (2H, m, H^{11}), 7.49 (2H, t, *J* = 7.7 Hz, H^{12}), 7,39 (1H, t, *J* = 7.3 Hz, H^{13}), 7.31 (1H, d, *J* = 8.4 Hz, H^3), 2.77 (3H, s, H^9). ^{13}C NMR (126 MHz, $CDCl_3$): δ 159.1 (C^2), 147.3 ($C^{8'}$), 140.6 (C^{10}), 138.6 (C^6), 136.5 (C^4), 129.2 (C^7), 129.1 (C^8), 129.0 (C^{12}), 127.7 (C^{13}), 127.5 (C^{11}), 126.3 (C^5), 125.3 ($C^{4'}$) 122.5 (C^3), 25.5 (C^9). ESI-MS (*m/z)*: Found $[M + H]^+$ 220.1121, calc $[C_{16}H_{13}N + H]^+$ 220.1121.

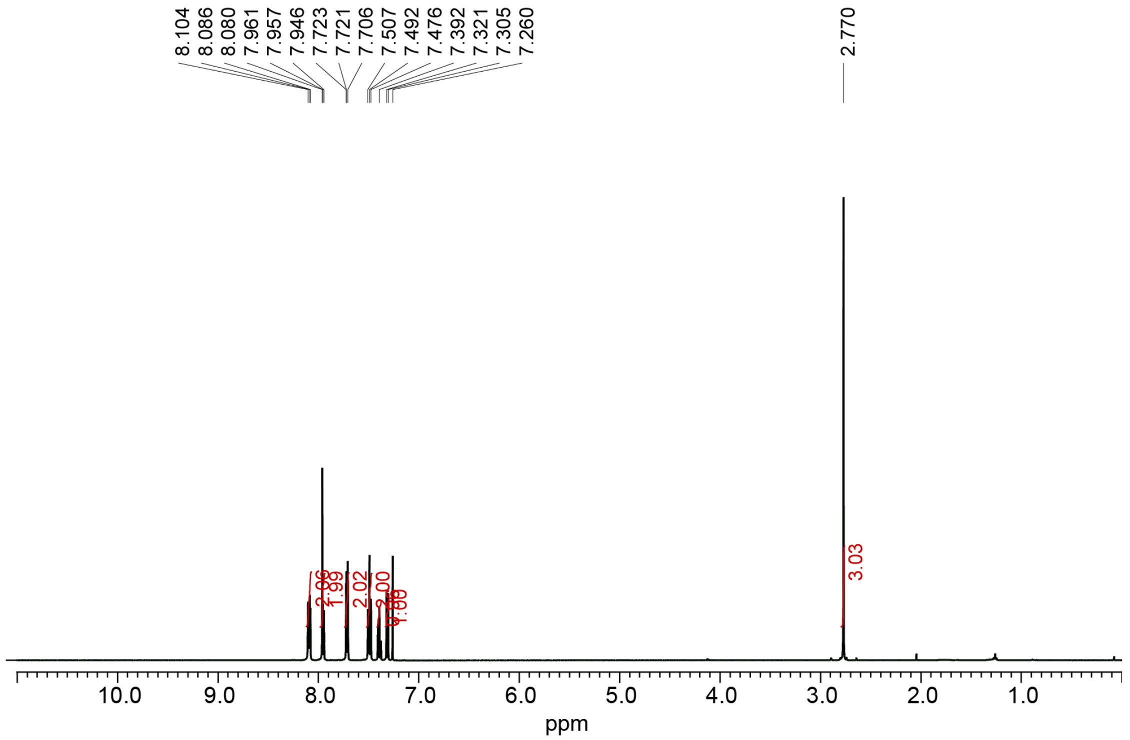

^{1}H NMR spectrum (500 MHz, $CDCl_3$, 298 K) of 2-methyl-6-phenylquinoline (**1a**).

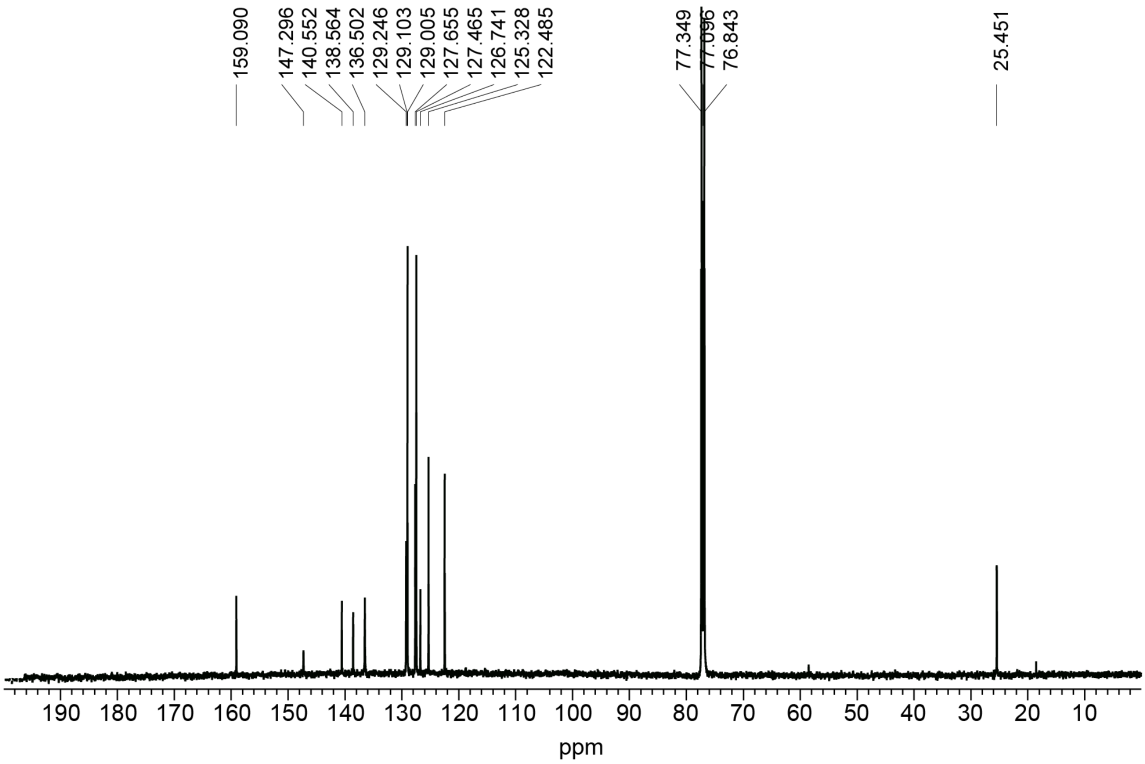

^{13}C NMR spectrum (126 MHz, $CDCl_3$, 298 K) of 2-methyl-6-phenylquinoline (**1a**).

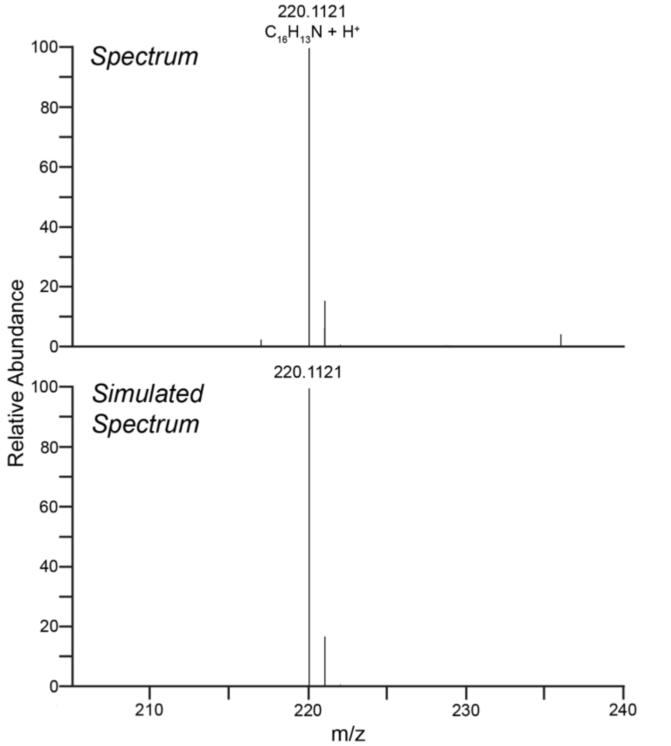

High-resolution mass spectra of 2-methyl-6-phenylquinoline (**1a**).

6-Phenyl-2-quinolinecarboxaldehyde (**2a**)

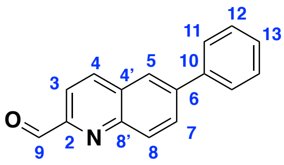

2-Methyl-6-phenylquinoline (0.49 g, 2.23 mmol) and anhydrous dioxane (30 mL) were added to oven-dried glassware under a nitrogen atmosphere. Selenium dioxide (0.50 g, 4.56 mmol) was added as one solid portion and the reaction was heated to 60 °C overnight. The reaction was cooled to room temperature then brine (20 mL) and ethyl acetate (30 mL) were added. The biphasic mixture was passed through a celite plug then the organic layer was separated. The aqueous layer was extracted with ethyl acetate (3 x 30 mL), organic layers combined and washed with brine (2 x 100 mL), dried ($MgSO_4$) and the solvent removed under reduced pressure, to obtain pure product as a pale-yellow solid (0.45 g, 87%).

^{1}H NMR (500 MHz, $CDCl_3$): δ 10.24 (1H, d, J = 0.6 Hz, H^9), 8.34 (1H, d, J = 8.5 Hz, H^4), 8.31 (1H, d, J = 8.8 Hz, H^8), 8.10 – 8.04 (3H, m, H^7, H^5, H^3), 7.75 – 7.73 (2H, m, H^{11}), 7.52 (2H, t, J = 7.6 Hz, H^{12}), 7.44 (1H, t, J = 7.4 Hz, H^{13}). ^{13}C NMR (126 MHz, $CDCl_3$): δ 193.7 (C^9), 152.6 (C^2), 147.4 ($C^{8'}$), 142.1 (C^6), 139.8 (C^{10}), 137.6 (C^4), 130.9 (C^8), 130.4 ($C^{4'}$), 130.4 (C^7), 129.2 (C^{12}), 128.4 (C^{13}), 127.6 (C^{11}), 125.4 (C^5), 117.9 (C^3). ESI-MS (*m/z)*: Found $[M + H]^+$ 234.0914, calc $[C_{16}H_{11}NO + H]^+$ 234.0913; Found $[M + Na]^+$ 256.0732, calc $[C_{16}H_{11}NO + Na]^+$ 256.0733.

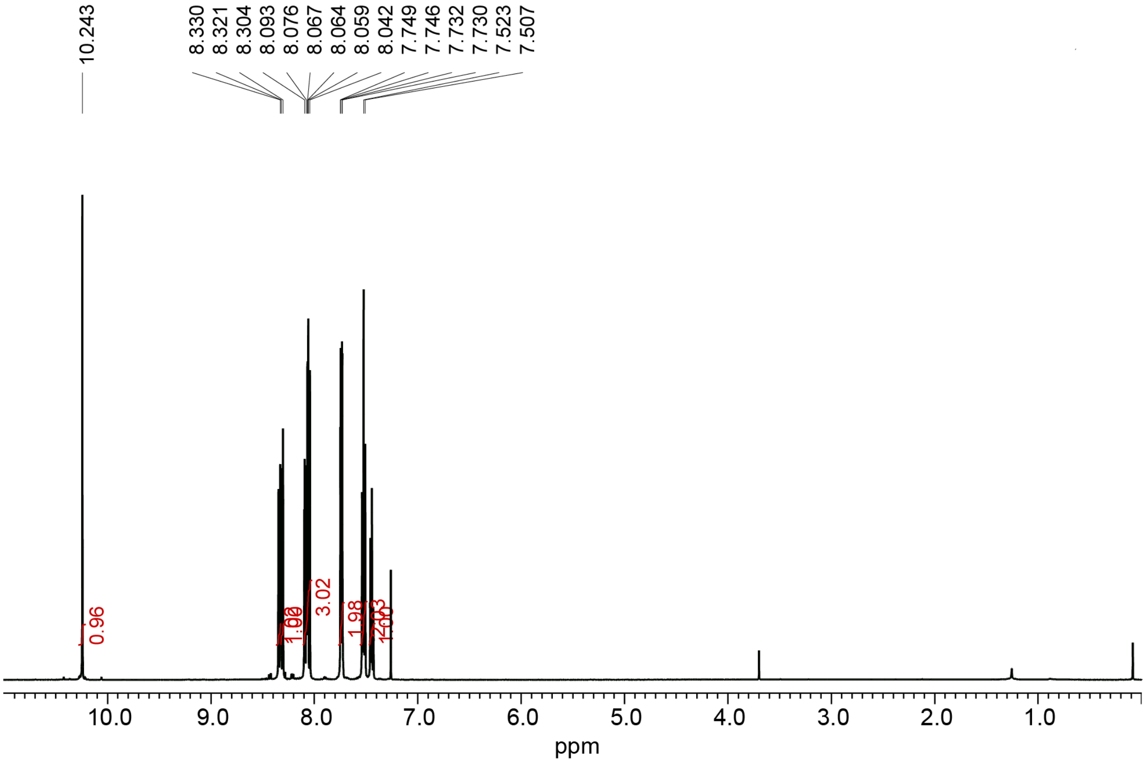

^{1}H NMR spectrum (500 MHz, $CDCl_3$, 298 K) of 6-phenyl-2-quinolinecarboxaldehyde (**2a**).

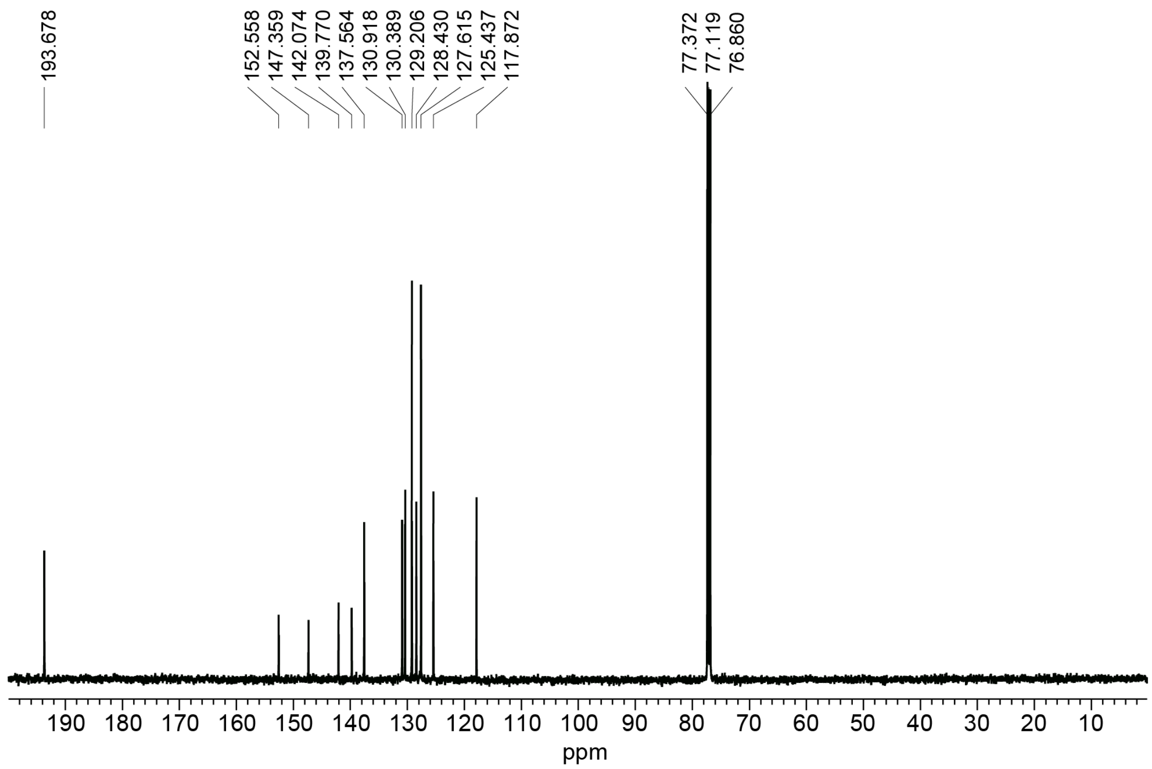

^{13}C NMR spectrum (126 MHz, $CDCl_3$, 298 K) of 6-phenyl-2-quinolinecarboxaldehyde (**2a**).

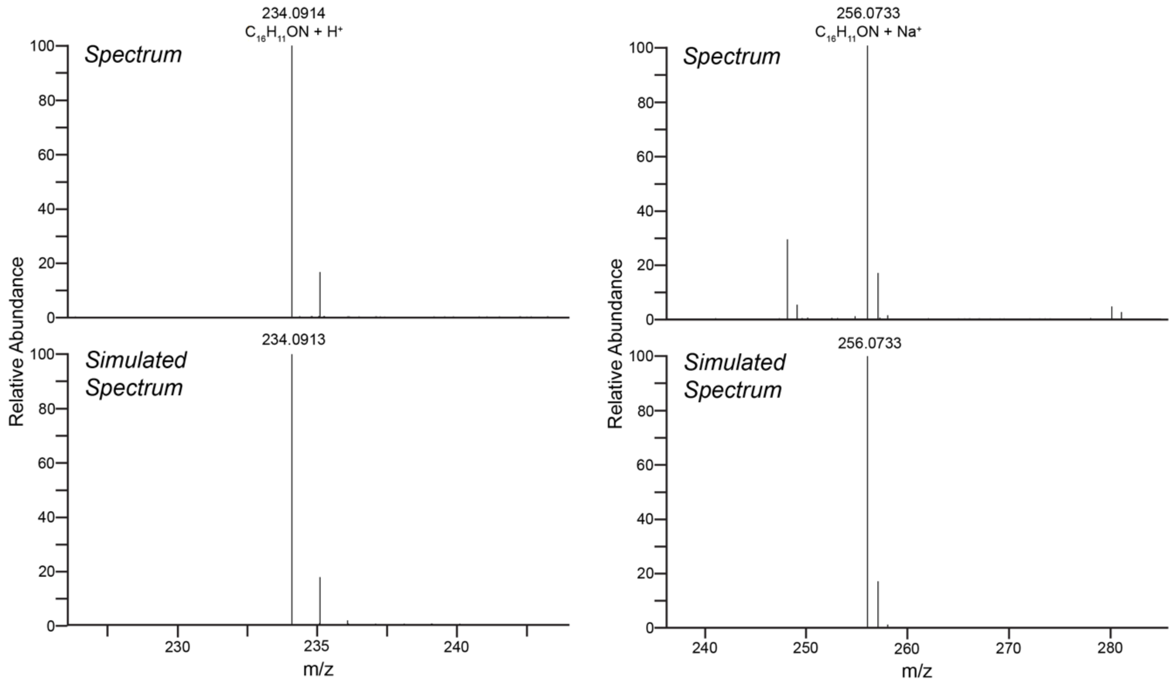

High-resolution mass spectra of 6-phenyl-2-quinolinecarboxaldehyde (**2a**).

6-Phenyl-2-quinolinemethanol (**3a**)

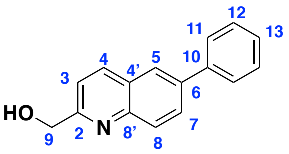

6-Phenyl-2-quinolinecarboxaldehyde (0.40, 1.72 mmol) was dissolved in anhydrous ethanol (10 mL) under a nitrogen atmosphere and cooled to 0 °C. Sodium borohydride (0.08 g, 2.06 mmol) was carefully added as one solid portion and stirred at 0 °C for one hour, then allowed to warm to room temperature. The reaction was quenched with NH_4Cl solution (20 mL) then the solvent evaporated under reduced pressure. The aqueous solution was extracted with chloroform (3 x 30 mL), organics combined, washed with brine (2 x 50 mL), dried ($MgSO_4$) and the solvent evaporated under reduced pressure to yield the product was a yellow solid (0.25, 61%).

^{1}H NMR (500 MHz, $CDCl_3$): δ 8.20 (1H, d, *J* = 8.5 Hz, H^4), 8.16 (1H, d, *J* = 8.5 Hz, H^8), 8.01 – 7.99 (2H, m, H^5, H^7), 7.72 (2H, d, *J* = 7.3 Hz, H^{11}), 7.51 (2H, t, *J* = 7.6 Hz, H^{12}), 7.41 (1H, t, *J* = 7.4 Hz, H^{13}), 7.33 (1H, d, *J* = 8.2 Hz, H^3), 4.95 (2H, s, H^9). ^{13}C NMR (126 MHz, $CDCl_3$): δ 159.0 (C^2), 146.0 ($C^{8'}$), 140.3 (C^{10}), 139.4 (C^6), 137.3 (C^4), 129.8 (C^7), 129.1 (C^{12}), 128.9 (C^8), 127.9 (C^{13}), 127.6 ($C^{4'}$), 127.5 (C^{11}), 125.5 (C^5), 118.9 (C^3), 64.2 (C^9). ESI-MS (*m/z)*: Found $[M + H]^+$ 236.1070, calc $[C_{16}H_{13}NO + H]^+$ 236.1070; Found $[M + Na]^+$ 258.0889, calc $[C_{16}H_{13}NO + Na]^+$ 258.0889.

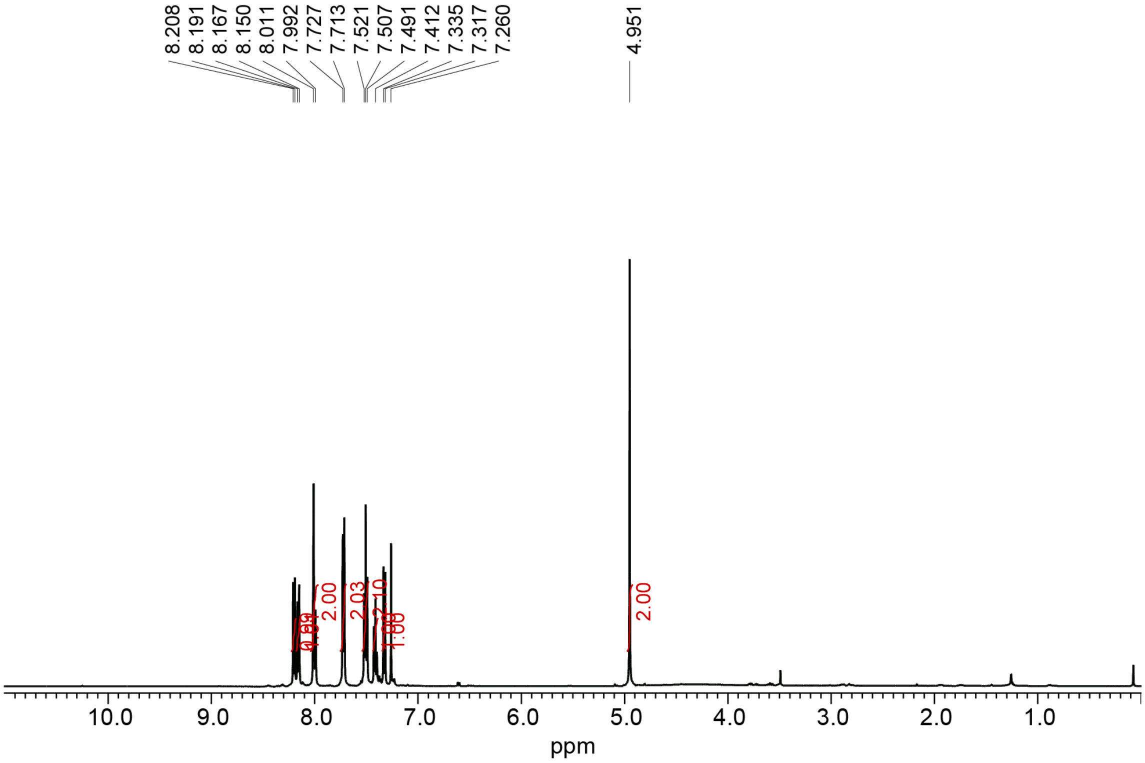

^{1}H NMR spectrum (500 MHz, $CDCl_3$, 298 K) of 6-phenyl-2-quinolinemethanol (**3a**).

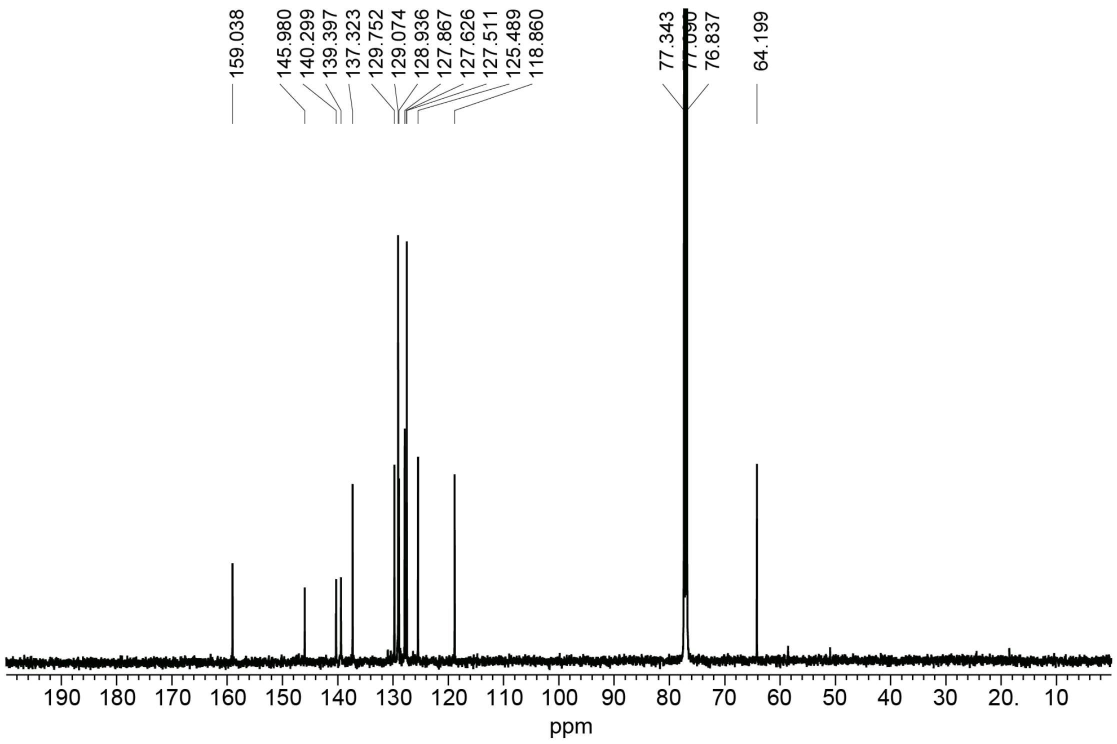

^{13}C NMR spectrum (126 MHz, $CDCl_3$, 298 K) of 6-phenyl-2-quinolinemethanol (**3a**).

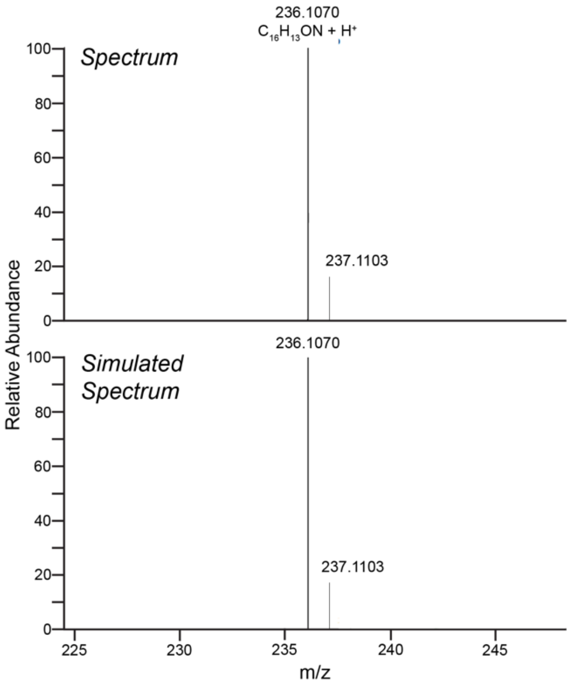

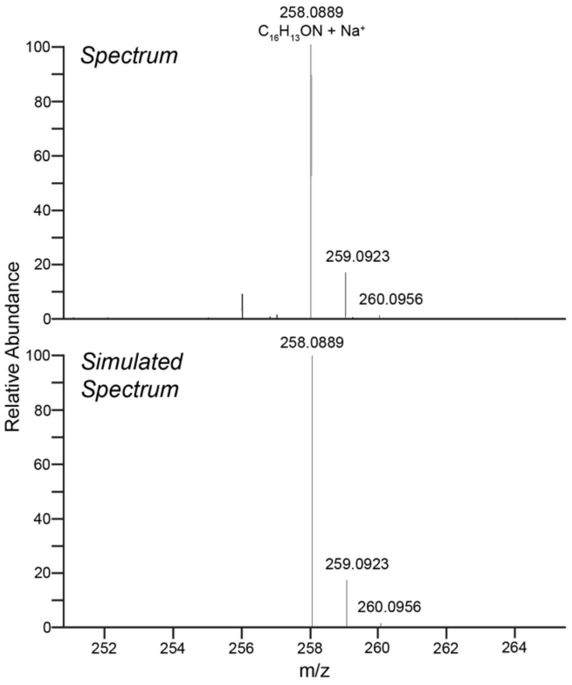

High-resolution mass spectra of 6-phenyl-2-quinolinemethanol (**3a**).

6-Phenyl-2-(chloromethyl)quinoline (**4a**)

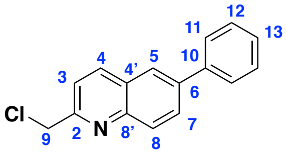

In an oven-dried Schlenk, 6-(2-phenylethynyl)-2-quinolinemethanol (0.40 g, 1.54 mmol) was dissolved in anhydrous dichloromethane (8 mL) under a nitrogen atmosphere. Diisopropylethylamine (0.40 mL, 2.31 mmol) and methanesulfonyl chloride (0.12 mL, 1.51 mmol) were added and the mixture was stirred for 1 hour. Water (20 mL) was added, and the organic layer separated. The aqueous layer was extracted with dichloromethane (2 x 20 mL) and the combined organic layers were washed with brine (2 x 50 mL), the organic layer dried ($MgSO_4$) and concentrated under reduced pressure. After storage in the freezer overnight, gave the chlorinated product 6-phenyl-2-(chloromethyl)quinoline as a yellow solid (0.25 g, 96%).

^{1}H NMR (500 MHz, $CDCl_3$): δ 8.26 (1H, d, *J* = 8.2 Hz, H^{4}), 8.15 (1H, d, *J* = 9.2 Hz, H^{8}), 8.01 – 8.00 (2H, m, H^{5}, H^{7}), 7.72 (2H, d, *J* = 7.3 Hz, H^{11}), 7.64 (1H, d, *J* = 8.5 Hz, H^{3}), 7.51 (2H, t, J = 7.6 Hz, H^{12}), 7.41 (1H, t, *J* = 7.3 Hz, H^{13}), 4.86 (2H, s, H^{9}). ^{13}C NMR (126 MHz, $CDCl_3$): δ 156.7 (C^{2}), 146.8 ($C^{8'}$), 140.2 (C^{10}), 139.9 (C^{6}), 137.6 (C^{4}), 129.8 (C^{7}), 129.7 (C^{8}), 129.1 (C^{12}), 128.0 (C^{13}), 127.7 ($C^{4'}$), 127.5 (C^{11}), 125.3 (C^{5}), 121.0 (C^{3}), 47.4 (C^{9}). ESI-MS (*m/z)*: Found $[M + H]^+$ 254.0731, calc $[C_{16}H_{12}NCl + H]^+$ 254.0731.

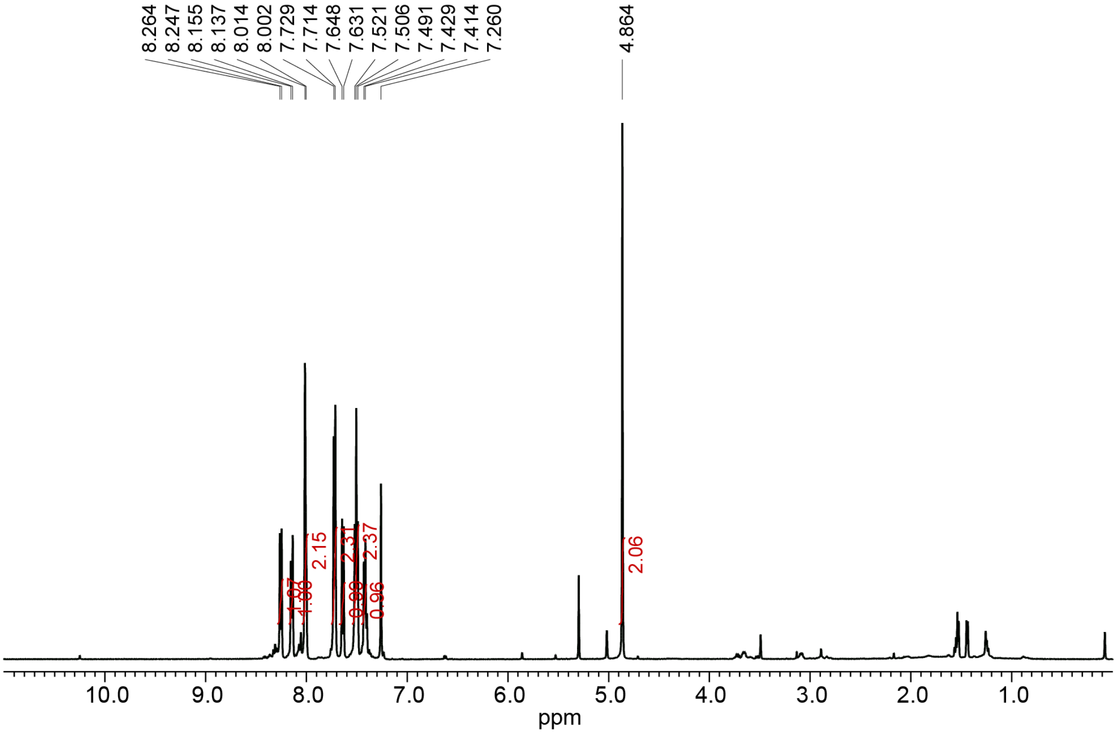

^{1}H NMR spectrum (500 MHz, $CDCl_3$, 298 K) of 6-phenyl-2-(chloromethyl)quinoline (**4a**).

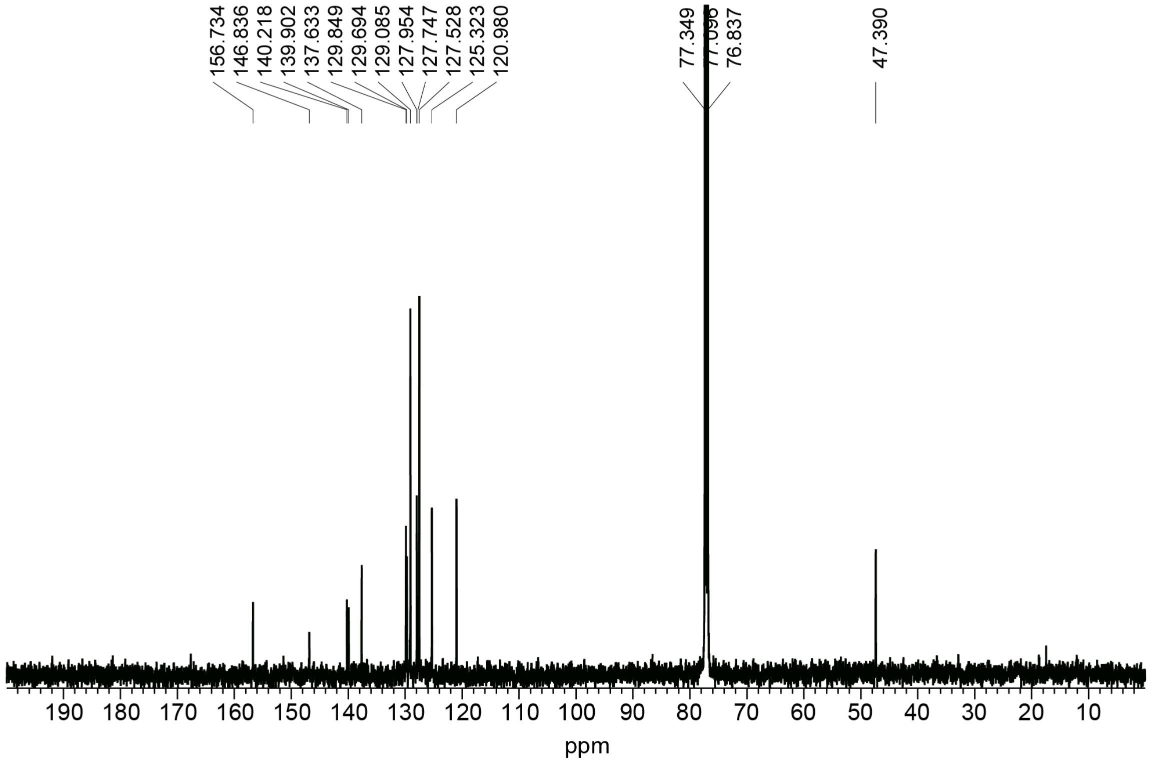

^{13}C NMR spectrum (126 MHz, $CDCl_3$, 298 K) of 6-phenyl-2-(chloromethyl)quinoline (**4a**).

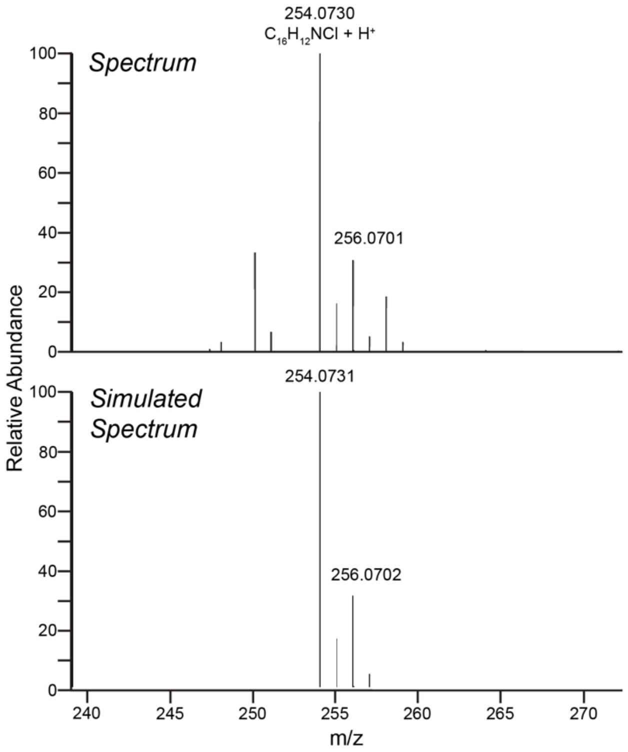

High-resolution mass spectra of 6-phenyl-2-(chloromethyl)quinoline (**4a**).

4,10-*Bis*((6-phenyl-quinolin-2-yl)-methyl)-1,4,7,10-tetraazacyclododecane-1,7-diyl)-diacetate (**5a**)

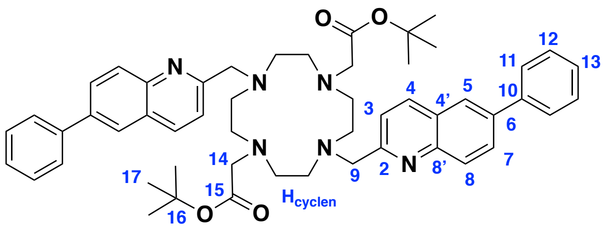

To an solution of DO2A-*tert*-butyl ester (0.11 g, 0.27 mmol) and potassium carbonate (0.12 g, 0.83 mmol) in anhydrous acetonitrile (10 mL), was added 6-phenyl-2-(chloromethyl)quinoline (0.25 g, 0.98 mmol). The yellow solution was stirred at 60 °C for 18 hours, after which potassium iodide was added (0.08 g, 0.48 mmol) and stirred at 60 °C for an additional 24 hours. The reaction was cooled to room temperature, salts removed through centrifugation (1500 rpm for 5 minutes). The organic layer was removed, and the salts washed with acetonitrile (2 x 8 mL). The organic layers combined, and the solvent removed under reduced pressure. The crude material was purified by column chromatography (silica gel; neat dichloromethane to 9:1 dichloromethane/methanol) to give the desired protected ligand, as a pale-yellow solid (0.156 g, 69%).

^{1}H NMR (500 MHz, $CDCl_3$): δ 8.26 (2H, J = 8.2 Hz, H^4), 8.01 – 7.98 (4H, m, H^5, H^8), 7.56 (4H, d, J = 7.3 Hz, H^{11}), 7.51 (2H, d, J = 8.8 Hz, H^7), 7.43 – 7.39 (6H, m, H^3, H^{12}), 7.36 – 7.33 (2H, m, H^{13}), 3.99 – 2.53 (16H, m, H^{cyclen}), 3.45 (4H, s, H^9), 2.93 (4H, s, H^{14}), 1.16 (18H, s, H^{17}). ^{13}C NMR (126 MHz, $CDCl_3$): δ 172.0 (C^{15}), 159.3 (C^2), 147.3 ($C^{8'}$), 140.0 (C^{10}), 139.0 (C^6), 137.5 (C^4), 130.1 (C^8), 129.3 (C^7), 129.1 (C^{12}), 127.9 ($C^{4'}$), 127.7 (C^{13}), 127.3 (C^{11}), 125.4 (C^5), 122.2 (C^3), 82.1 (C^{16}), 60.2 (C^9), 58.0 (C^{14}), 51.1 (C^{cyclen}), 50.8 (C^{cyclen}), 28.1 (C^{17}). ESI-MS (*m/z)*: Found $[M + H]^+$ 835.4905, calc $[C_{52}H_{62}N_6O_4 + H]^+$ 835.4905; Found $[M + Na]^+$ 857.4725, calc $[C_{52}H_{62}N_6O_4 + Na]^+$ 857.4725.

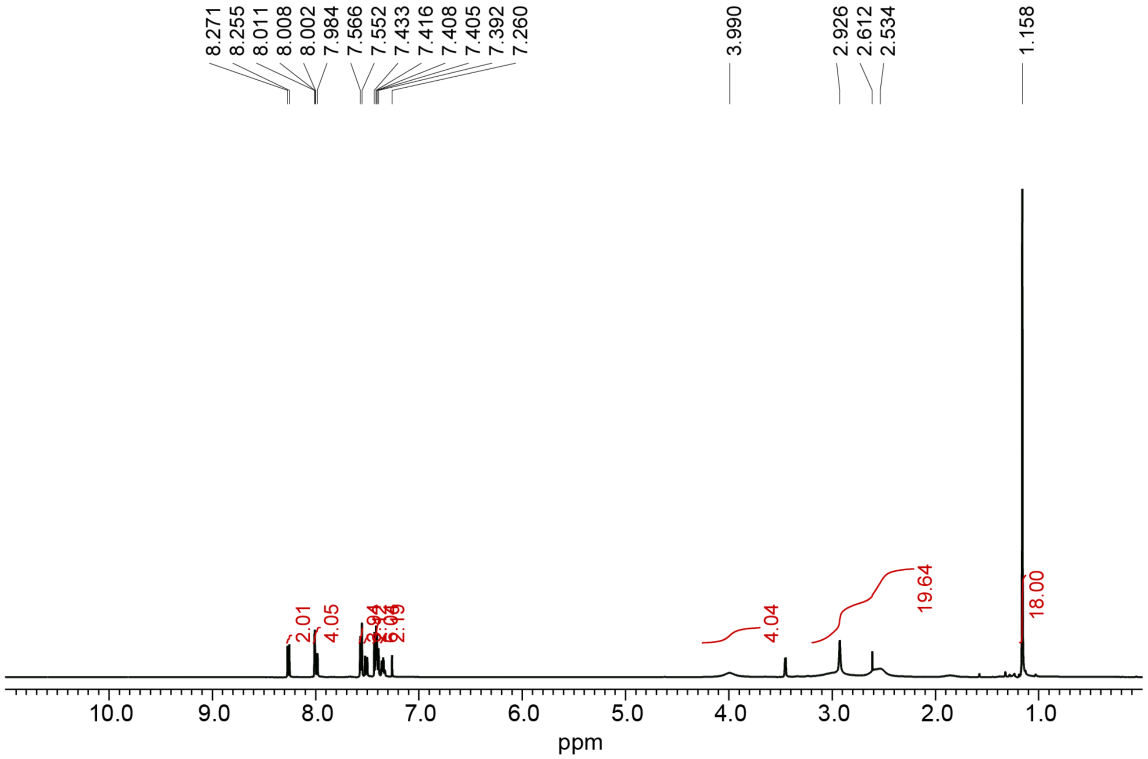

^{1}H NMR (500 MHz, $CDCl_3$, 298 K) of 4,10-*bis*((6-phenyl-quinolin-2-yl)-methyl)-1,4,7,10-tetraazacyclododecane-1,7-diyl)-diacetate (**5a**).

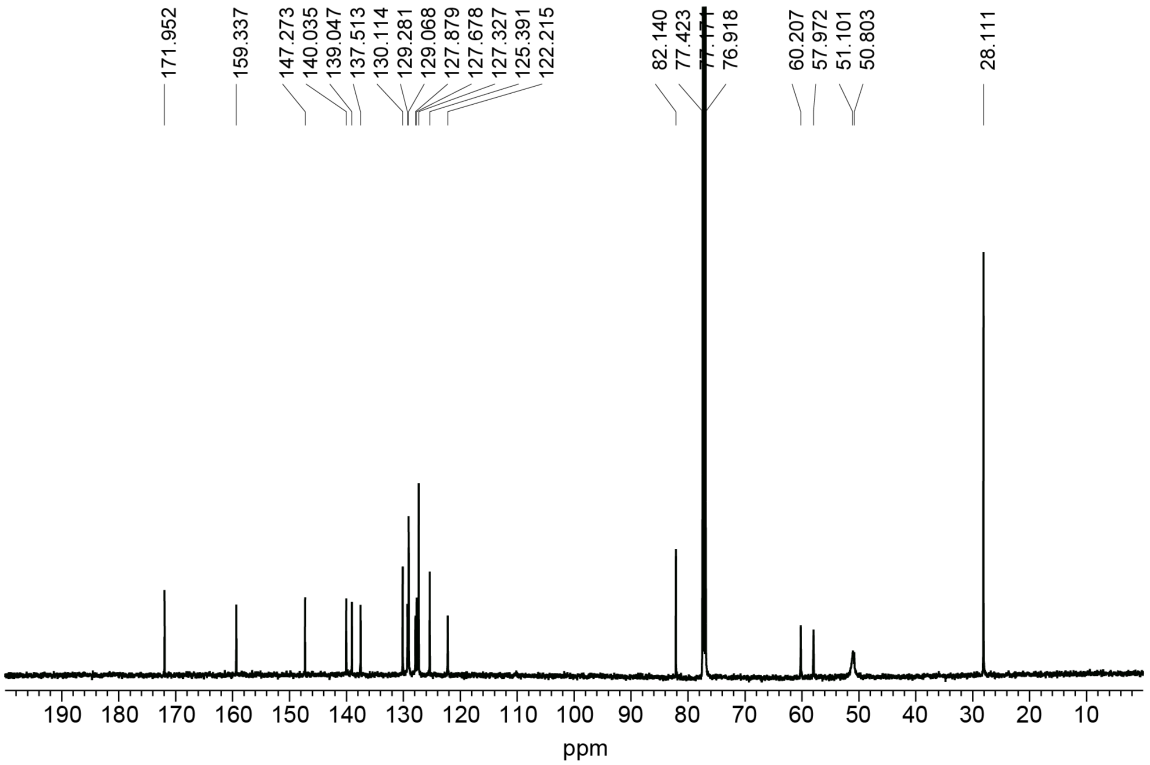

^{13}C NMR spectrum (126 MHz, $CDCl_3$, 298 K) of 4,10-*bis*((6-phenyl-quinolin-2-yl)-methyl)-1,4,7,10-tetraazacyclododecane-1,7-diyl)-diacetate (**5a**).

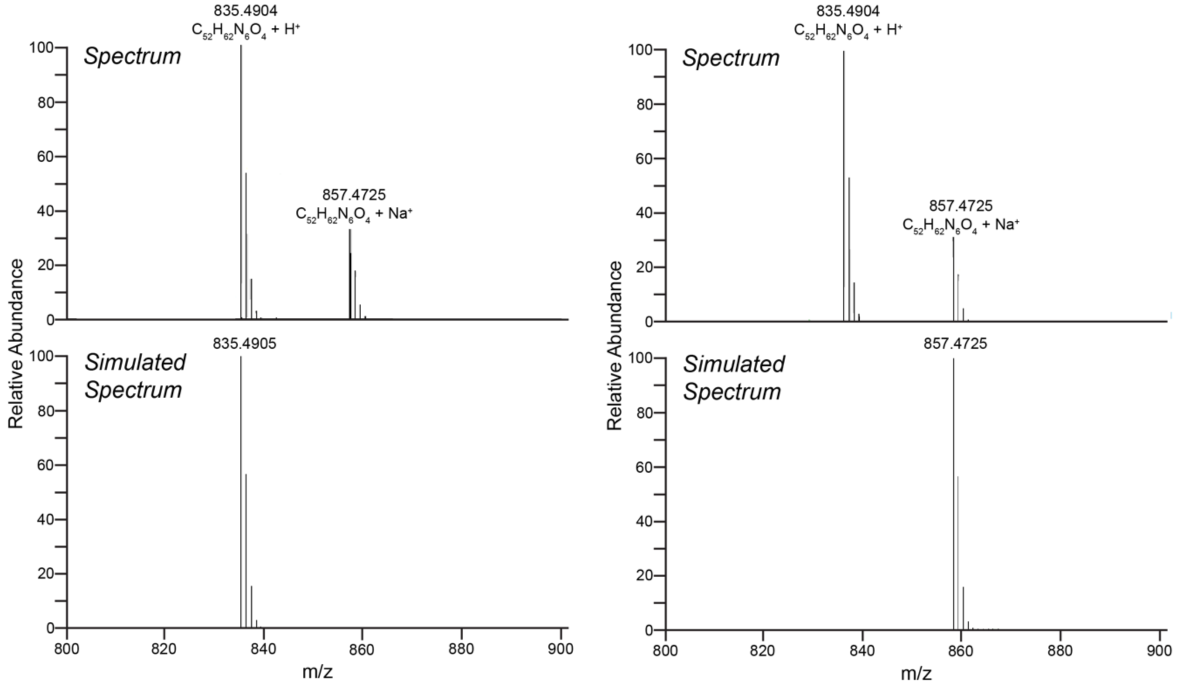

High-resolution mass spectra of 4,10-*bis*((6-phenyl-quinolin-2-yl)-methyl)-1,4,7,10-tetraazacyclododecane-1,7-diyl)-diacetate (**5a**).

4,10-*Bis*((6-phenyl-quinolin-2-yl)-methyl)-1,4,7,10-tetraazacyclododecane-1,7-diyl)-diacetic acid (**6a**)

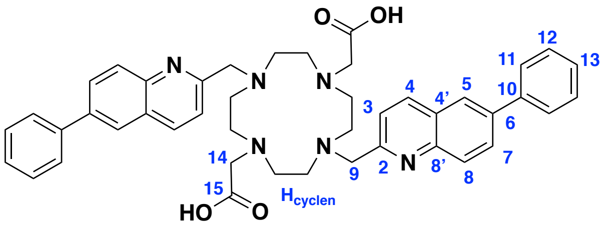

To a solution of 4,10-*bis*((6-phenyl-quinolin-2-yl)-methyl)-1,4,7,10-tetraazacyclododecane-1,7-diyl)-diacetate (80 mg, 98 μmol) in dichloromethane (1 mL) was added trifluoroacetic acid (1 mL). The reaction mixture was stirred at room temperature for 16 hours and the trifluoroacetic acid was co-evaporated with dichloromethane (5 x 25 mL) to give the deprotected ligand as a pale brown solid (71 mg, quant.).

^{1}H NMR (500 MHz, $CDCl_3$): δ 8.52 (2H, d, *J* = 8.5 Hz, H^4), 8.22 (2H, s, H^5), 8.09 (2H, d, *J* = 8.8 Hz, H^8), 7.60 – 7.56 (6H, m, H^{11}, H^3), 7.44 (2H, dd, *J* = 8.8 Hz, 1.9 Hz, H^7), 7.36 – 7.35 (6H, m, H^{12}, H^{13}), 5.07 (4H, s, H^9), 3.90 – 3.83 (12H, m, H^{14}, H^{cyclen}), 3.45 – 3.28 (8H, m, H^{cyclen}). ^{13}C NMR (126 MHz, $CDCl_3$): δ 172.4 (C^{15}), 151.5 (C^2), 146.0 ($C^{8'}$), 139.9 (C^6), 139.5 (C^{10}), 138.5 (C^4), 129.7 (C^7), 129.3 (C^8), 128.8 (C^{12}), 128.4 (C^{13}), 127.7 ($C^{4'}$), 126.9 (C^{11}), 124.9 (C^5), 120.1 (C^3), 58.7 (C^9), 52.8 (C^{14}), 52.2 (C^{cyclen}), 48.0 (C^{cyclen}). ESI-MS (*m*/*z*): Found $[M + H]^+$ 723.3652, calc $[C_{44}H_{46}N_6O_4 + H]^+$ 723.3653; Found $[M + Na]^+$ 745.3472, calc $[C_{44}H_{46}N_6O_4 + Na]^+$ 745.3473; Found $[M + 2H]^{2+}$ 362.1861, calc $[C_{44}H_{46}N_6O_4 + 2H]^{2+}$ 362.1861.

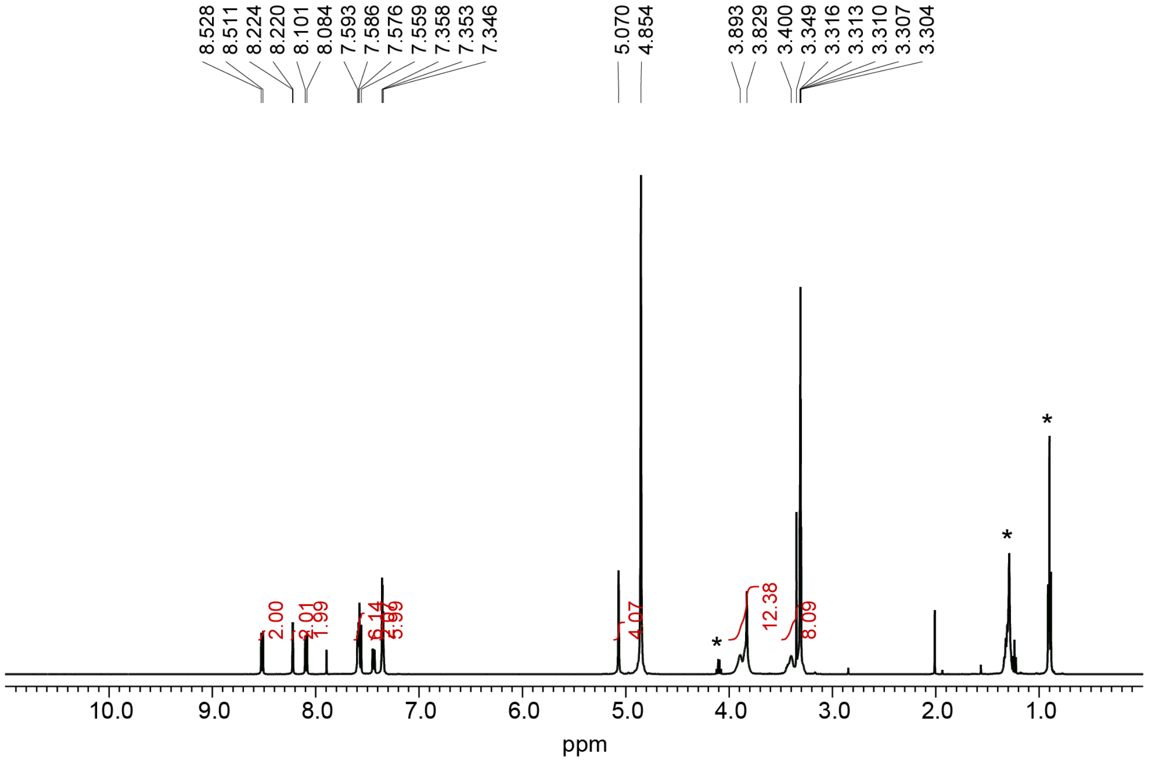

^{1}H NMR spectrum (500 MHz, $CDCl_3$, 298 K) of 4,10-*bis*((6-phenyl-quinolin-2-yl)-methyl)-1,4,7,10-tetraazacyclododecane-1,7-diyl)-diacetic acid (**6a**).

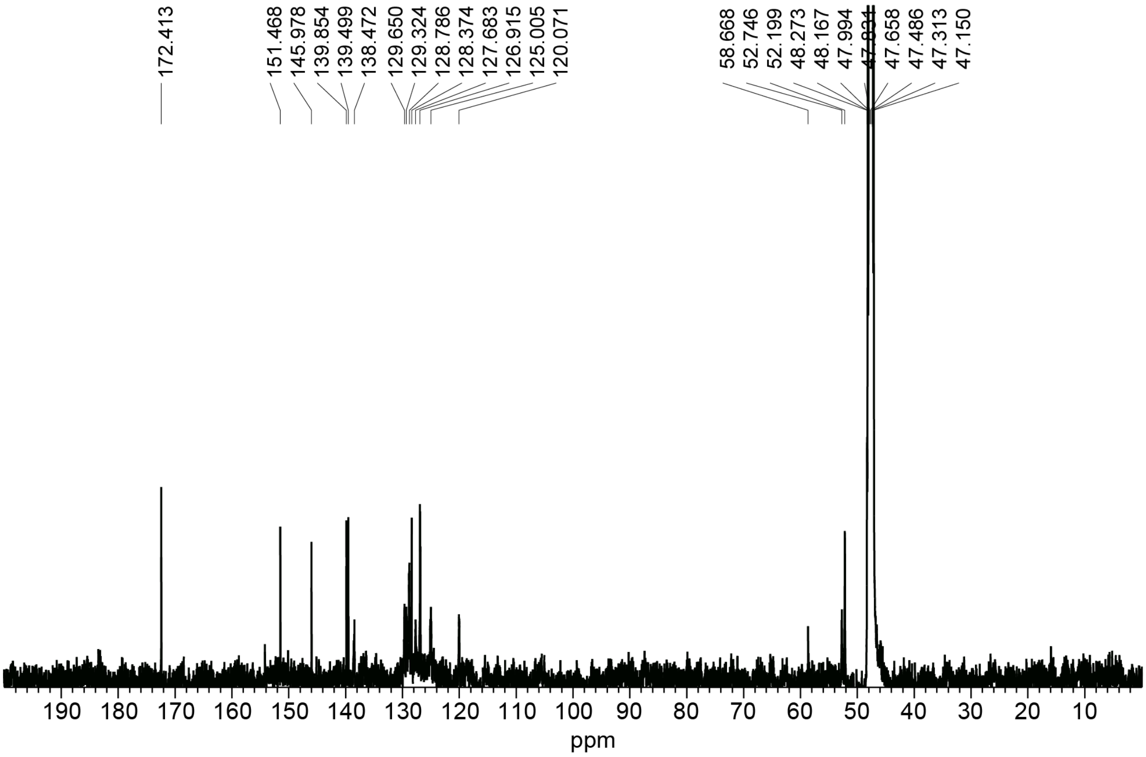

^{13}C NMR spectrum (126 MHz, $CDCl_3$, 298 K) of 4,10-*bis*((6-phenyl-quinolin-2-yl)-methyl)-1,4,7,10-tetraazacyclododecane-1,7-diyl)-diacetic acid (**6a**).

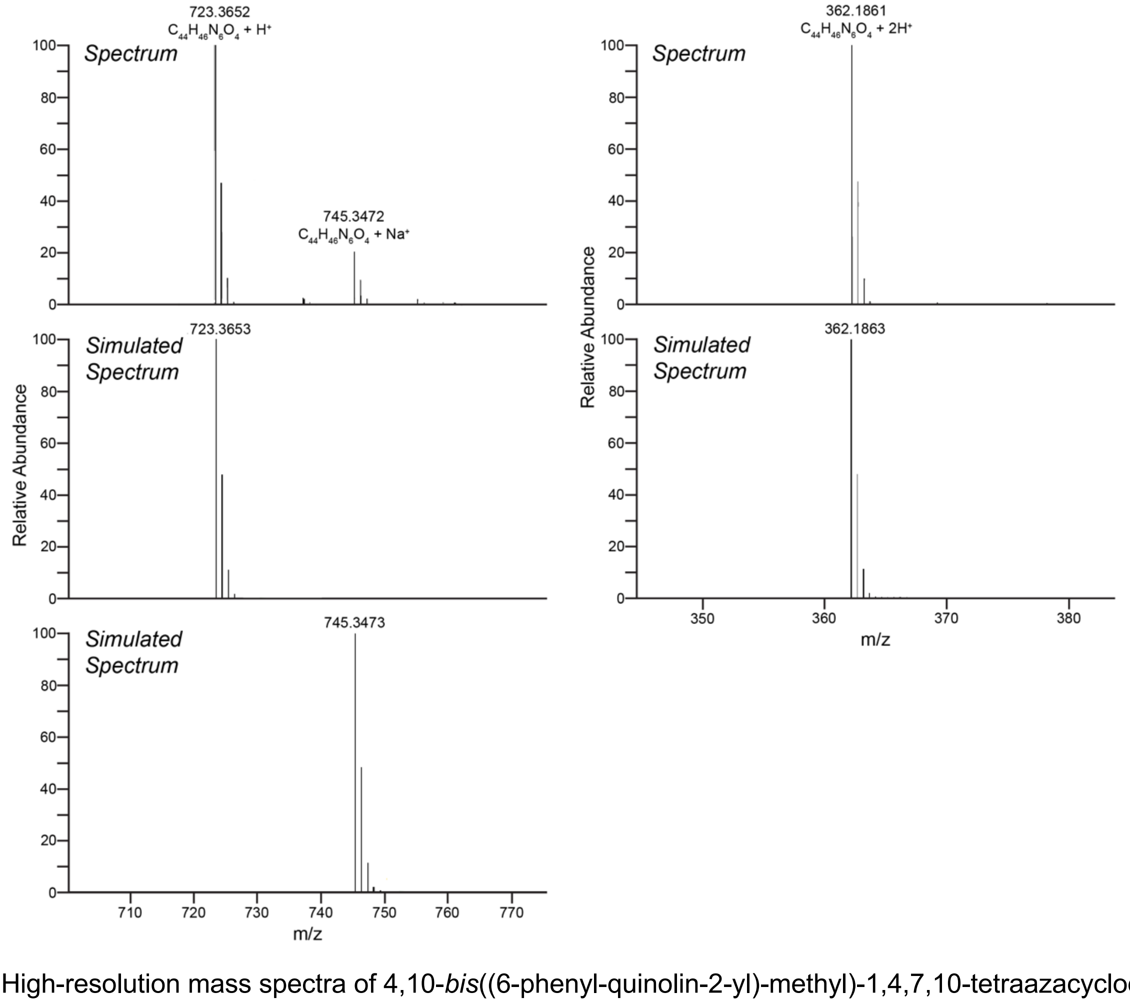

High-resolution mass spectra of 4,10-*bis*((6-phenyl-quinolin-2-yl)-methyl)-1,4,7,10-tetraazacyclododecane-1,7-diyl)-diacetic acid (**6a**).

[Eu.6Ph]$^+$

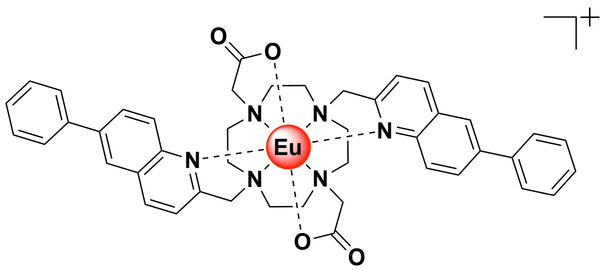

The deprotected ligand (50 mg, 0.05 mmol) was dissolved in methanol (3 mL). Potassium carbonate (2 mg, 0.01 mmol) and europium(III) trifluoromethanesulfonate (46 mg, 0.08 mmol) were added and the reaction heated to 50 °C for 48 hours. The reaction was cooled to room temperature, filtered and the solvent evaporated under reduced pressure. The crude material was purified by column chromatography (silica gel; neat dichloromethane to 4:1 dichloromethane/methanol) to give the desired complex as an off-white solid (16 mg, 30%).

^{1}H NMR (500 MHz, CD_3OD): δ 51.9, 30.4, 17.6, 17.5, 17.2, 15.7, 15.5, 13.7, 13.1, 12.1, 11.5, 8.6, 8.3, 8.2, 7.7, 7.6, 7.3, 6.6, 6.2, 5.9, 5.6, 4.9, 4.8, 3.3, 3.3, 2.1, 1.3, 0.8, 0.5, -0.4, -2.0, -3.0, -5.7, -9.5, -11.1, -11.3, -16.5, -17.0, -17.7, -28.6, -29.7, -30.5, -34.3. ESI-MS (*m/z*): Found [M]$^+$ 873.2630, calc $[C_{44}H_{44}EuN_6O_4]^+$ 873.2631. Photophysical data measured in methanol: λ_{max} = 328 nm, ε = 9000 M^{-1} cm^{-1}, Φ_{em} = 9.6%, τ_{CH3OH} = 0.83 ms, τ_{CD3OD} = 1.30 ms, *m* = 0.8.

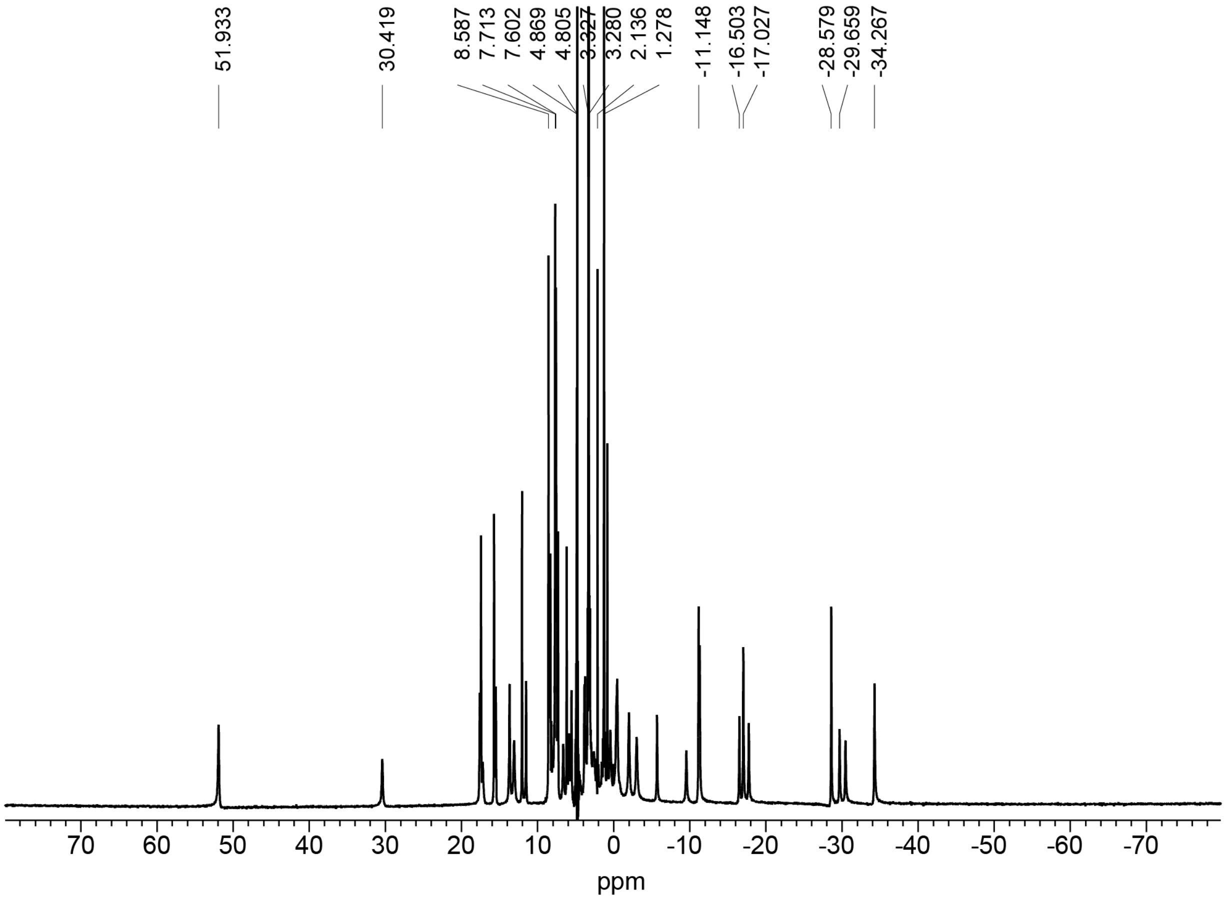

^{1}H NMR spectrum (500 MHz, CD_3OD, 298 K) of **[Eu.6Ph]**$^+$.

[Gd.6Ph]$^+$

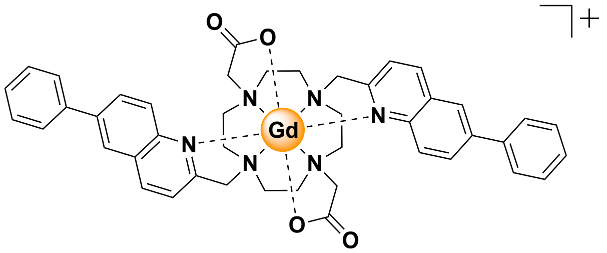

The deprotected ligand (10 mg, 0.01 mmol) was dissolved in methanol (2 mL). Potassium carbonate (4 mg, 0.03 mmol) and gadolinium(III) trifluoromethanesulfonate (17 mg, 0.03 mmol) were added and the reaction heated to 50 °C for 4 hours. The reaction was cooled to room temperature, filtered and the solvent evaporated under reduced pressure. The crude material was purified by column chromatography (silica gel; neat dichloromethane to 4:1 dichloromethane/methanol) to give the desired complex as a white solid (13 mg, 51%).

ESI-MS (*m/z*): Found $[M]^+$ 878.2665, calc $[C_{44}H_{44}GdN_6O_4]^+$ 878.2660. Photophysical data measured in methanol: λ_{max} = 329 nm.

6-(4-Methoxyphenyl)-2-methylquinoline (**1b**)

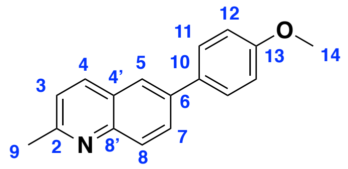

6-Bromoquinaldine (0.60 g, 2.70 mmol), 4-methoxyphenyl boronic acid (0.49 g, 3.24 mmol) and potassium carbonate (1.12 g, 8.11 mmol) were added to an oven-dried Schlenk with a condenser attached under a nitrogen atmosphere. Anhydrous dioxane (24 mL) and oxygen-free water (6 mL) were added, followed by palladium-tetrakis(triphenylphosphine) (0.21 g, 0.27 mmol) and the reaction was heated to 60 °C for 16 hours. The reaction was cooled to room temperature, filtered through celite and the filtrate was removed under reduced pressure. The residue was dissolved in dichloromethane (50 mL) then washed with water (50 mL). The organic layer was separated, and the aqueous layer washed with dichloromethane (3 x 50 mL). The organic layers combined, washed with brine (100 mL), dried ($MgSO_4$) and solvent evaporated under reduced pressure. The product was obtained after column chromatography (silica gel; 5:95 ethyl acetate/hexane) to give the product as a white solid (0.42, 63%).

^{1}H NMR (500 MHz, $CDCl_3$): δ 8.09 – 8.06 (2H, m, H^8, H^4), 7.92 – 7.89 (2H, m, H^7, H^5), 7.64 (2H, d, J = 8.8 Hz, H^{11}), 7.29 (1H, d, J = 8.2 Hz, H^3), 7.02 (2H, d, J = 8.8 Hz, H^{12}), 3.87 (3H, s, H^{14}), 2.76 (3H, s, H^9). ^{13}C NMR (126 MHz, $CDCl_3$): δ 159.5 (C^{13}), 158.7 (C^2), 146.9 ($C^{8'}$), 138.2 (C^6), 136.5 (C^4), 132.9 (C^{10}), 129.1 (C^7), 128.9 (C^8), 128.5 (C^{11}), 126.8 ($C^{4'}$), 124.5 (C^5), 122.5 (C^3), 114.5 (C^{12}), 55.5 (C^{14}), 25.3 (C^9). ESI-MS (*m/z)*: Found $[M + H]^+$ 250.1227, calc $[C_{17}H_{15}NO + H]^+$ 250.1226.

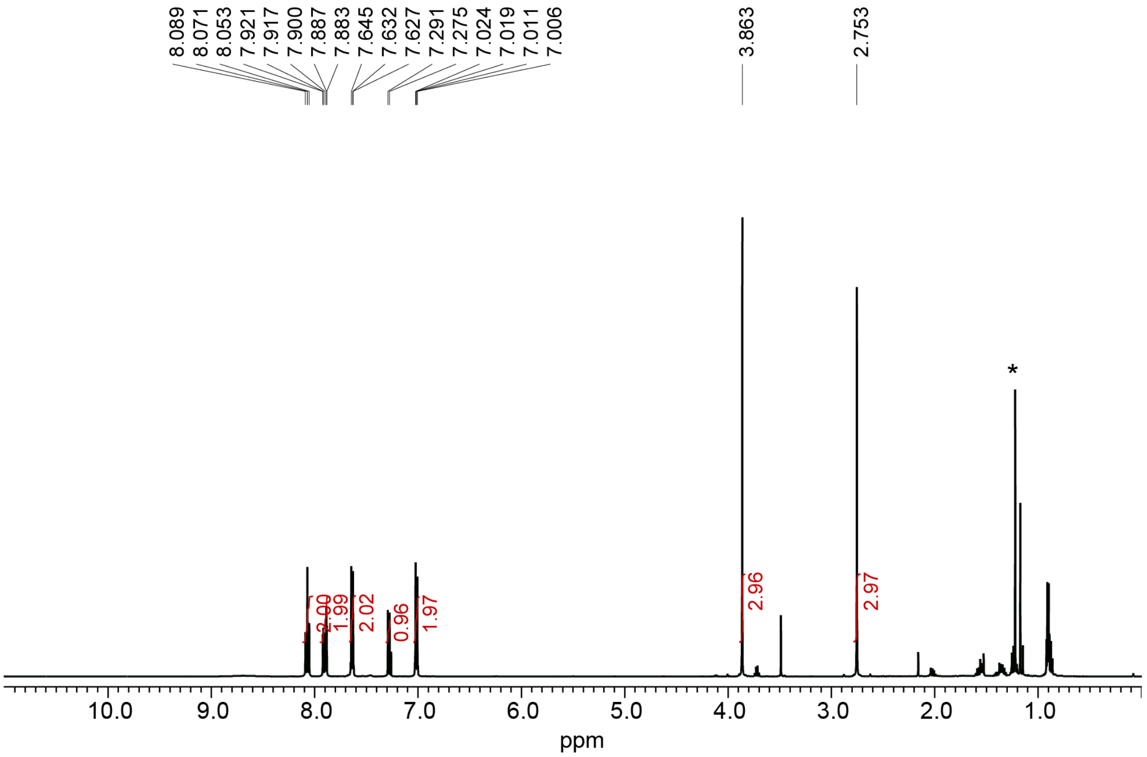

^{1}H NMR spectrum (500 MHz, $CDCl_3$, 298 K) of 6-(4-methoxyphenyl)-2-methylquinoline (**1b**).

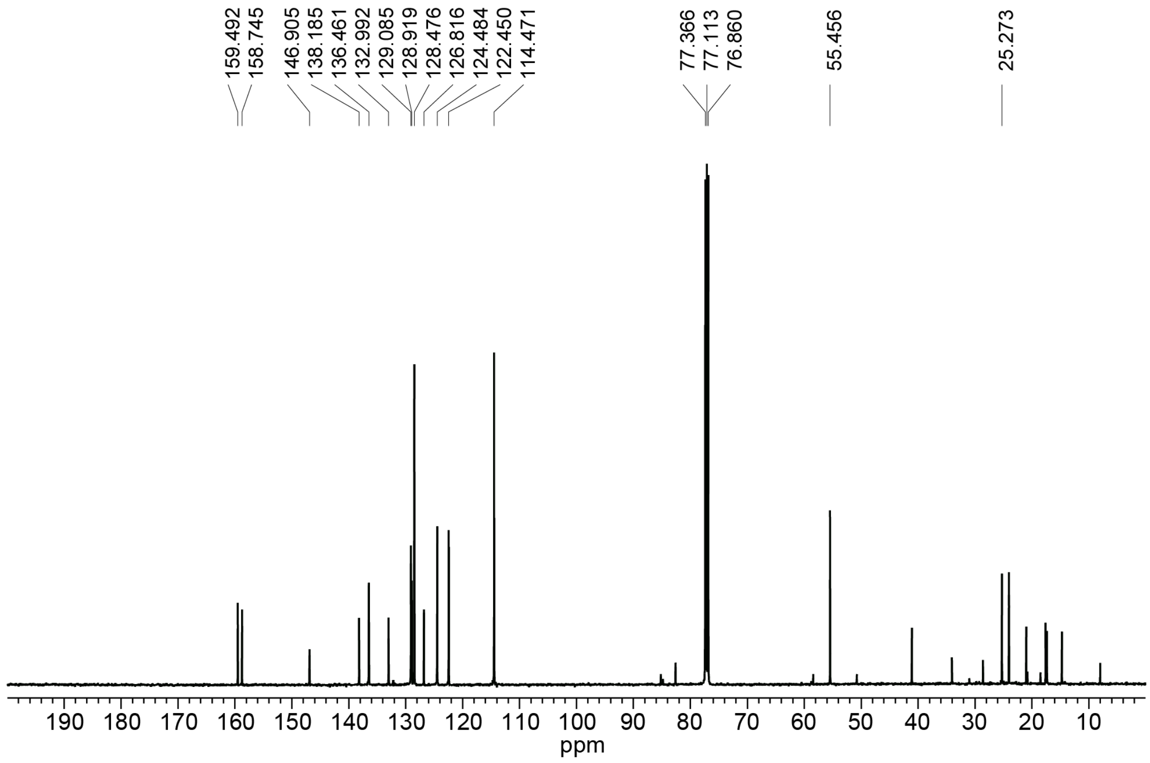

^{13}C NMR spectrum (126 MHz, $CDCl_3$, 298 K) of 6-(4-methoxyphenyl)-2-methylquinoline (**1b**).

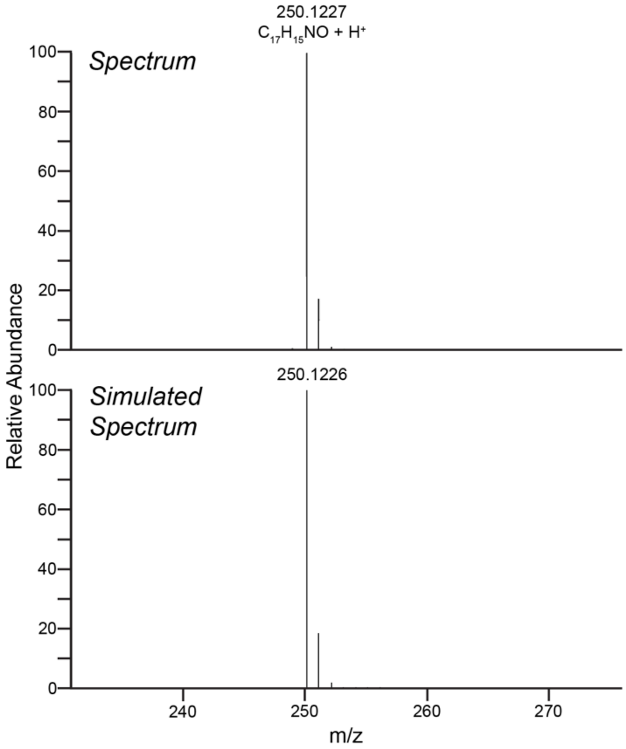

High-resolution mass spectra of 6-(4-methoxyphenyl)-2-methylquinoline (**1b**).

6-(4-Methoxyphenyl)-2-quinolinecarboxaldehyde (**2b**)

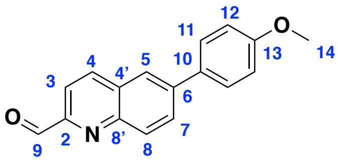

6-(4-Methoxyphenyl)-2-methylquinoline (0.32 g, 1.29 mmol) and anhydrous dioxane (20 mL) were added to an oven-dried glassware under a nitrogen atmosphere. Selenium dioxide (0.30 g, 2.73 mmol) was added as one solid portion, and the reaction was heated to 60 °C for 16 hours. The reaction was cooled to room temperature then brine (20 mL) and ethyl acetate (20 mL) were added. The biphasic mixture was passed through a celite plug then the organic layer was separated. The aqueous layer was extracted with ethyl acetate (3 x 20 mL), organic layers combined and washed with brine (2 x 50 mL), dried ($MgSO_4$) and the solvent removed under reduced pressure, to obtain pure product as a pale-yellow solid (0.30, 88%).

^{1}H NMR (500 MHz, $CDCl_3$): δ 10.27 (1H, s, H^9), 8.35 (1H, d, *J* = 8.5 Hz, H^4), 8.32 (1H, d, *J* = 8.8 Hz, H^8), 8.08 (1H, dd, *J* = 8.8 Hz, 1.9 Hz, H^7), 8.06 (1H, d, *J* = 8.2 Hz, H^3), 8.04 (1H, d, *J* = 1.9 Hz, H^5), 7.70 (2H, d, *J* = 8.8 Hz, H^{11}), 7.06 (2H, d, *J* = 8.8 Hz, H^{12}), 3.90 (3H, s, H^{14}). ^{13}C NMR (126 MHz, $CDCl_3$): δ 193.3 (C^9), 160.2 (C^{13}), 152.0 (C^2), 146.7 (C$^{8'}$), 141.8 (C^6), 137.7 (C^4), 132.1 (C^{10}), 130.6 (C$^{4'}$), 130.5 (C^7), 130.4 (C^8), 128.7 (C^{11}), 124.5 (C^5), 117.9 (C^3), 114.7 (C^{12}), 55.5 (C^{14}). ESI-MS (*m/z)*: Found [M + H]$^+$ 264.1019, calc [$C_{17}H_{13}NO_2$ + H]$^+$ 264.1019; Found [M + Na]$^+$ 286.0838, calc [$C_{17}H_{13}NO_2$ + Na]$^+$ 286.0838.

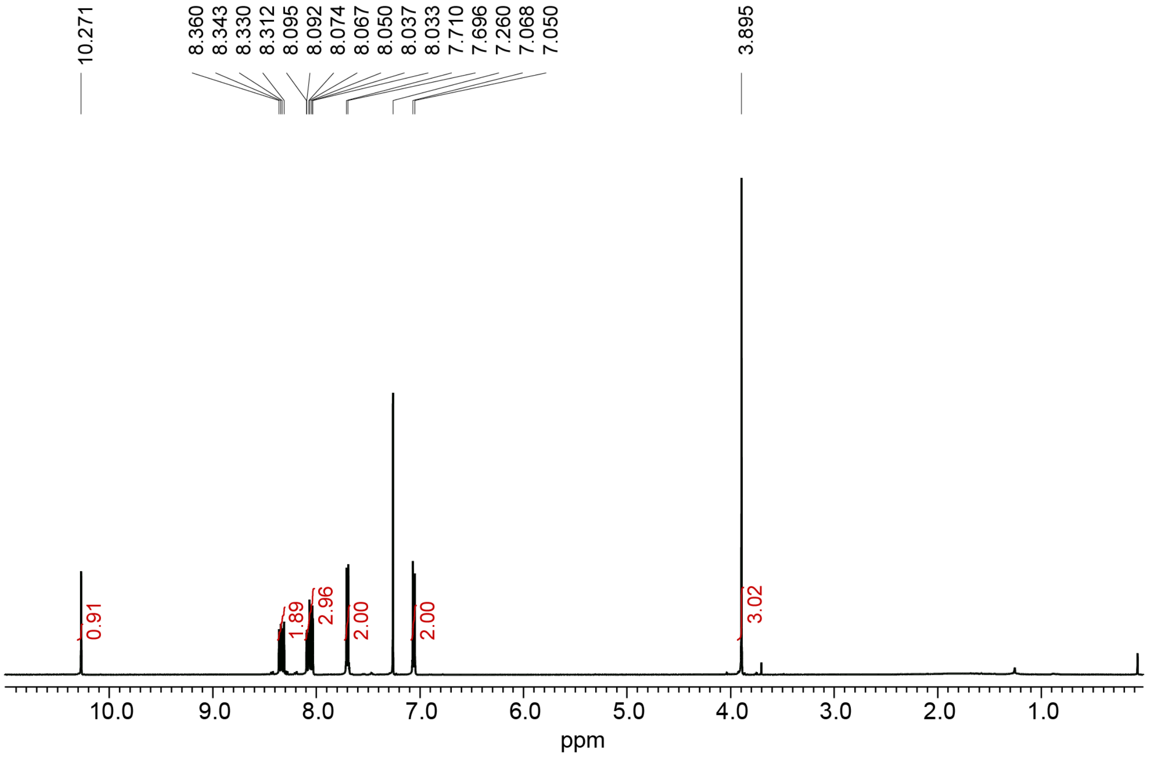

^{1}H NMR spectrum (500 MHz, $CDCl_3$, 298 K) of 6-(4-methoxyphenyl)-2-quinolinecarboxaldehyde (**2b**).

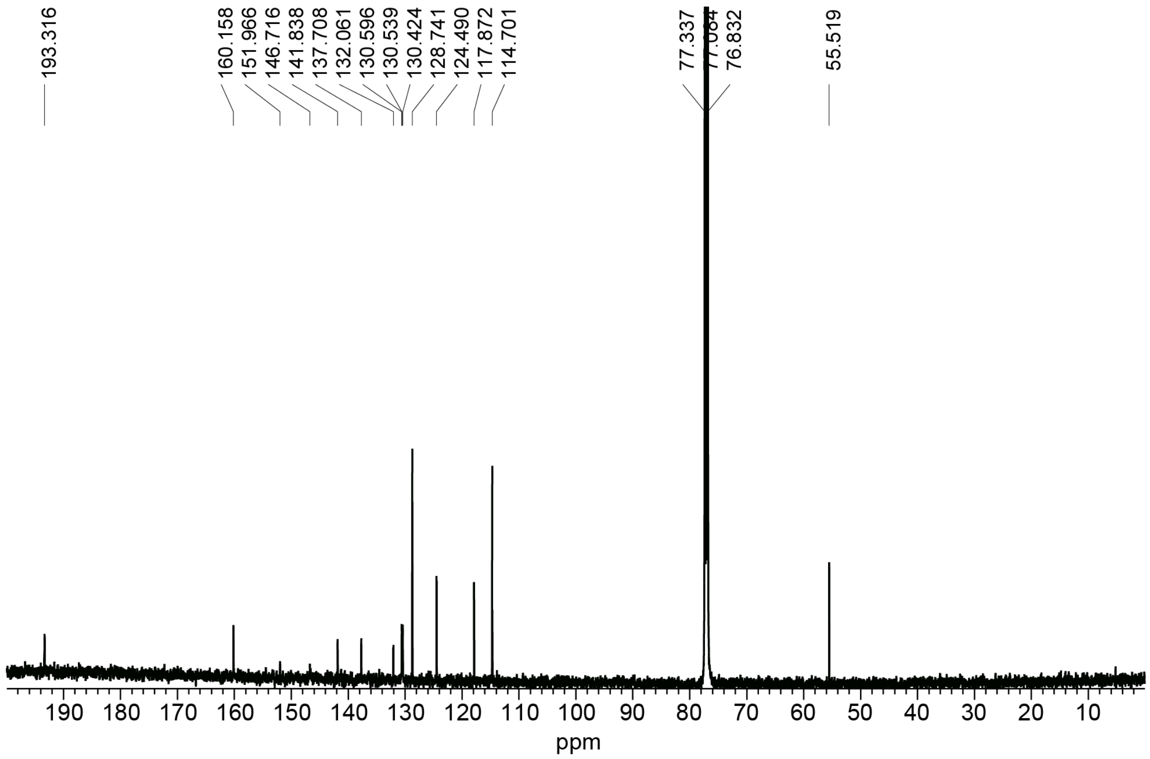

^{13}C NMR spectrum (126 MHz, $CDCl_3$, 298 K) of 6-(4-methoxyphenyl)-2-quinolinecarboxaldehyde (**2b**).

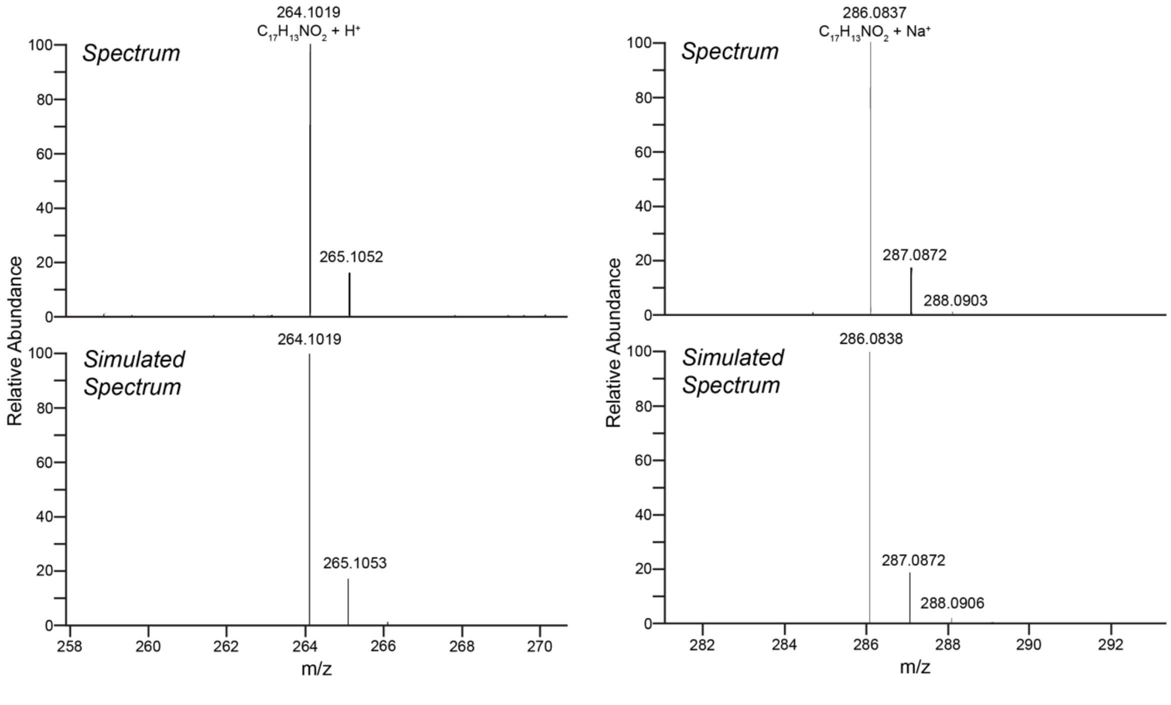

High-resolution mass spectra of 6-(4-methoxyphenyl)-2-quinolinecarboxaldehyde (**2b**).

6-(4-Methoxyphenyl)-2-quinolinemethanol (**3b**)

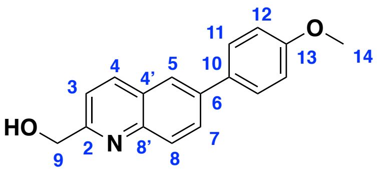

6-(4-Methoxyphenyl)-2-quinolinecarboxaldehyde (0.24, 1.04 mmol) was dissolved in anhydrous ethanol (10 mL) under a nitrogen atmosphere. Sodium borohydride (0.05 g, 1.24 mmol) was carefully added as one solid portion and stirred at room temperature for 2 hours. The reaction was quenched with NH_4Cl solution (10 mL) then the ethanol was evaporated under reduced pressure. The aqueous solution was extracted with chloroform (3 x 20 mL), organics combined, washed with brine (2 x 30 mL), dried ($MgSO_4$) and the solvent evaporated under reduced pressure to yield the product as a yellow solid (0.24, 87%).

^{1}H NMR (500 MHz, $CDCl_3$): δ 8.19 (1H, d, J = 8.5 Hz, H^4), 8.15 (1H, d, J = 8.5 Hz, H^8), 7.99 – 7.96 (2H, m, H^7, H^5), 7.66 (2H, d, J = 8.8 Hz, H^{11}), 7.32 (1H, d, J = 8.2 Hz, H^3), 7.04 (2H, d, J = 8.5 Hz, H^{12}), 4.95 (2H, s, H^9), 3.88 (3H, s, H^{14}). ^{13}C NMR (126 MHz, $CDCl_3$): δ 159.7 (C^{13}), 158.7 (C^2), 145.5 ($C^{8'}$), 139.1 (C^6), 137.4 (C^4), 132.7 (C^{10}), 129.7 (C^7), 128.6 (C^8), 128.6 (C^{11}), 128.0 ($C^{4'}$), 124.7 (C^5), 118.8 (C^3), 114.6 (C^{12}), 64.1 (C^9), 55.5 (C^{14}). ESI-MS (*m/z)*: Found $[M + H]^+$ 266.1175, calc $[C_{17}H_{15}NO_2 + H]^+$ 266.1176; Found $[M + Na]^+$ 288.0994, calc $[C_{17}H_{15}NO_2 + Na]^+$ 288.0995.

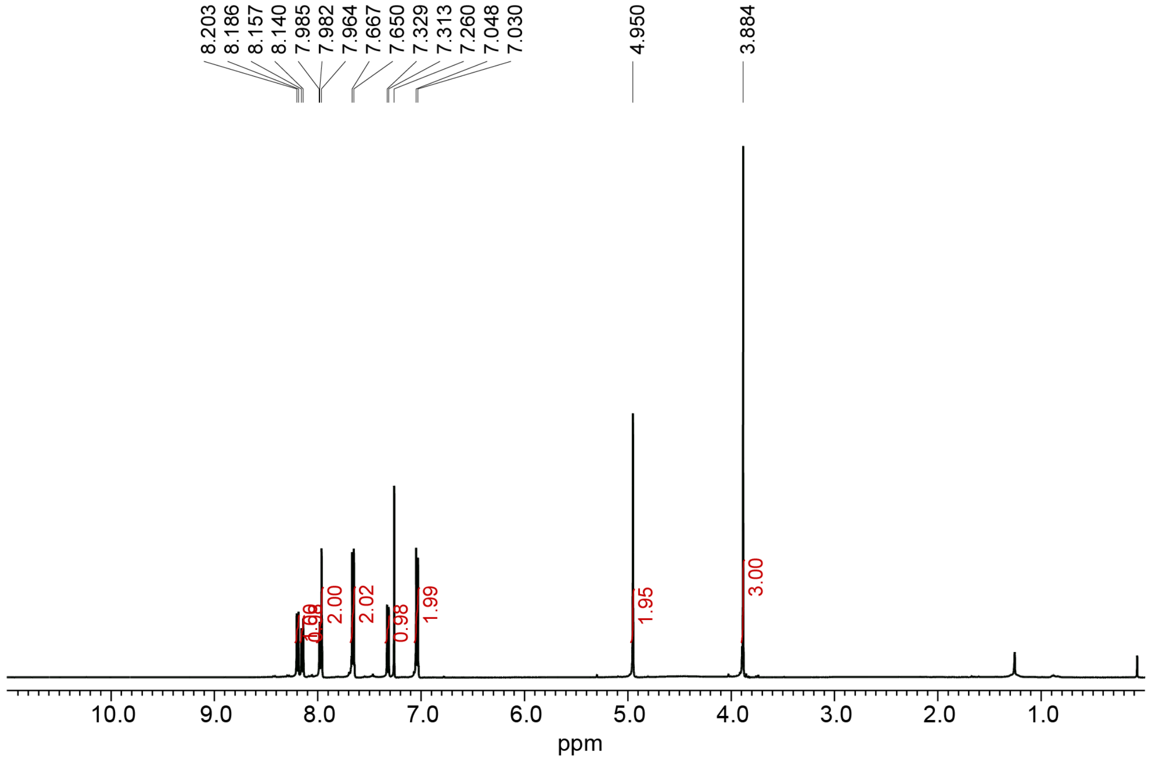

^{1}H-NMR spectrum (500 MHz, $CDCl_3$, 298 K) of 6-(4-methoxyphenyl)-2-quinolinemethanol (**3b**).

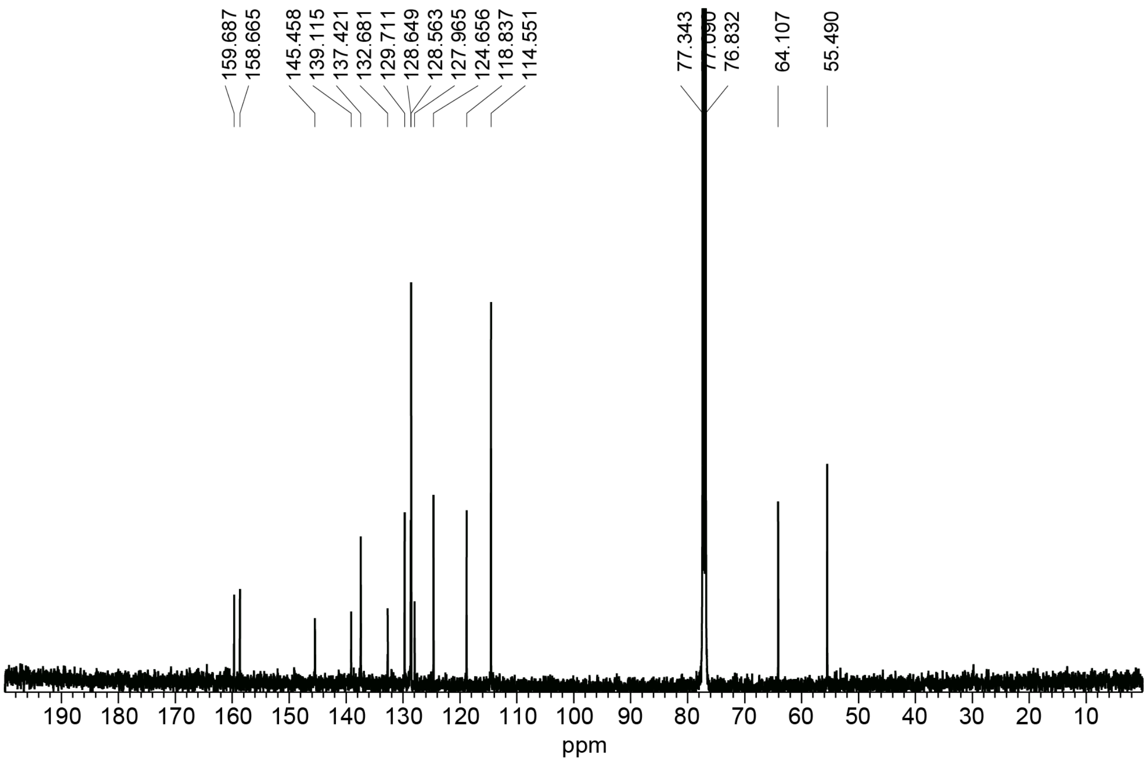

^{13}C-NMR spectrum (126 MHz, $CDCl_3$, 298 K) of 6-(4-methoxyphenyl)-2-quinolinemethanol (**3b**).

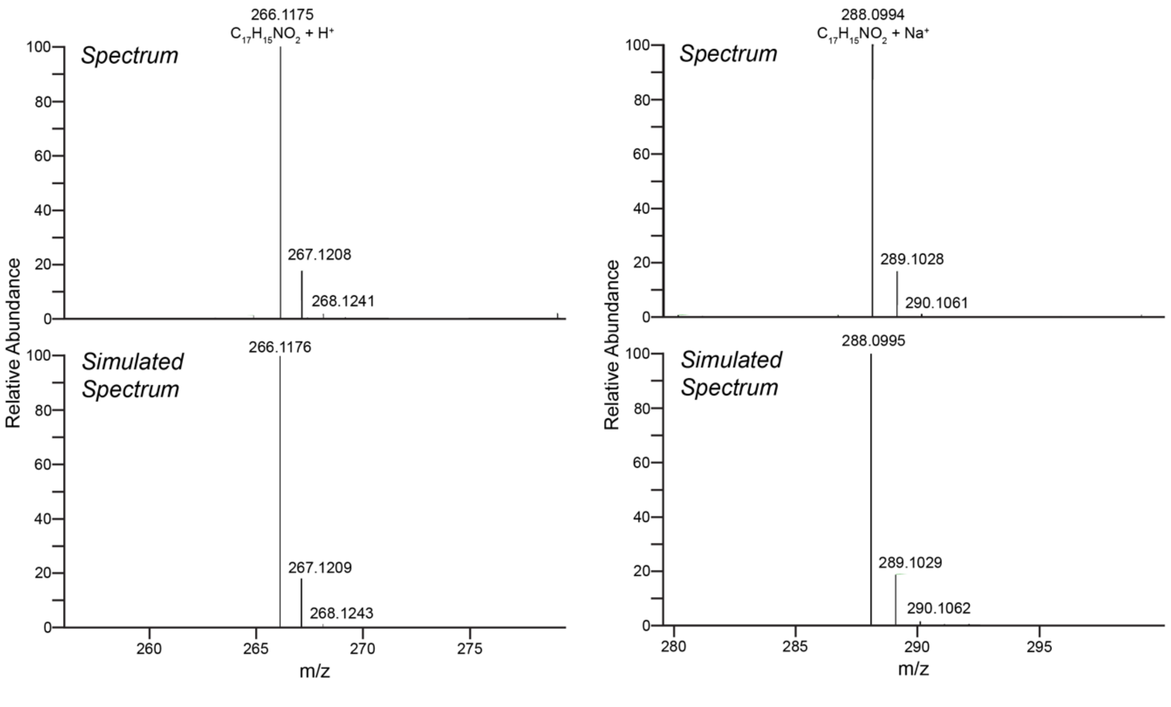

High-resolution mass spectra of 6-(4-methoxyphenyl)-2-quinolinemethanol (**3b**).

6-(4-Methoxyphenyl)-2-methanesulfonate-2-quinolinemethanol (**4b**)

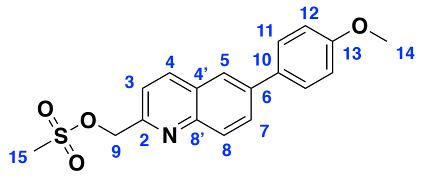

In an oven-dried Schlenk, 6-(4-methoxyphenyl)-2-quinolinemethanol (0.24 g, 0.91 mmol) was dissolved in anhydrous dichloromethane (5 mL) under a nitrogen atmosphere. Diisopropylethylamine (0.23 mL, 1.35 mmol) and methanesulfonyl chloride (0.09 mL, 0.88 mmol) were added and the mixture was stirred at room temperature for 2 hours. Water (20 mL) was added, and the organic layer separated. The aqueous layer was extracted with dichloromethane (2 x 30 mL) and the combined organic layers were washed with brine (50 mL), the organic layer dried ($MgSO_4$) and concentrated under reduced pressure, to give the product as a yellow solid (0.28 g, 93%).

^{1}H NMR (500 MHz, $CDCl_3$): δ 8.26 (1H, d, J = 8.2 Hz, H^{4}), 8.10 (1H, d, J = 8.8 Hz, H^{8}), 7.99 – 7.97 (2H, m, H^{7}, H^{5}), 7.66 (2H, d, J = 8.8 Hz, H^{11}), 7.60 (1H, d, J = 8.2 Hz, H^{3}), 7.04 (2H, d, J = 8.8 Hz, H^{12}), 5.51 (2H, s, H^{9}), 3.88 (3H, s, H^{14}), 3.12 (3H, s, H^{15}). ^{13}C NMR (126 MHz, $CDCl_3$): δ 159.8 (C^{13}), 153.5 (C^{2}), 146.8 ($C^{8'}$), 139.7 (C^{6}), 137.5 (C^{4}), 132.5 (C^{10}), 129.8 (C^{7}), 129.7 (C^{8}), 128.6 (C^{11}), 128.1 ($C^{4'}$), 124.5 (C^{5}), 120.1 (C^{3}), 114.6 (C^{12}), 72.3 (C^{9}), 55.5 (C^{14}), 38.2 (C^{15}). LR-MS ESI (m/z): Found $[M + H]^+$ 343.8, calc $[C_{18}H_{17}NO_4S + H]^+$ 344.1.

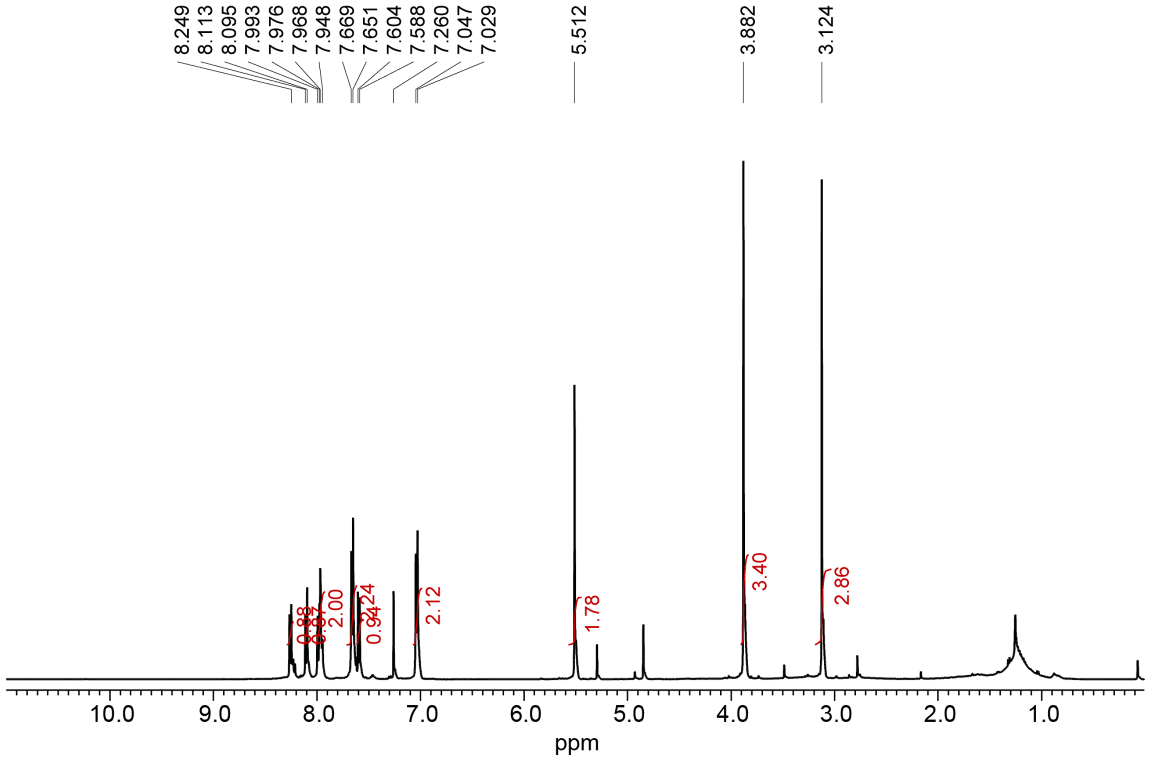

^{1}H NMR spectrum (500 MHz, $CDCl_3$, 298 K) of 6-(4-methoxyphenyl)-2-methanesulfonate-2-quinolinemethanol (**4b**).

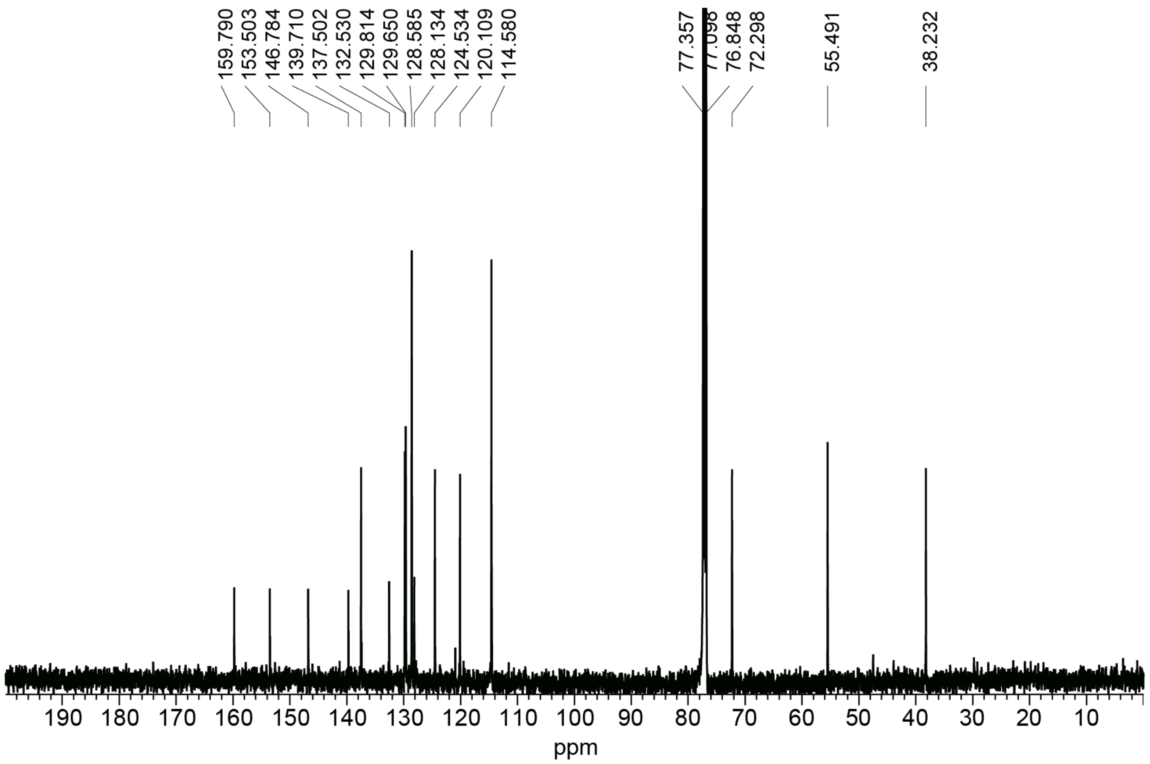

^{13}C NMR spectrum (126 MHz, $CDCl_3$, 298 K) of 6-(4-methoxyphenyl)-2-methanesulfonate-2-quinolinemethanol (**4b**).

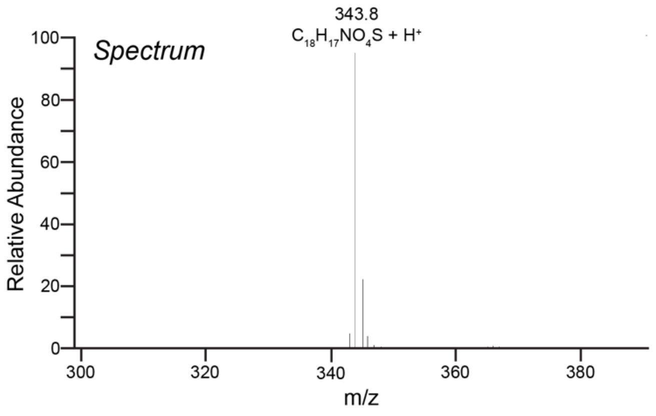

Low-resolution mass spectrum of 6-(4-methoxyphenyl)-2-methanesulfonate-2-quinolinemethanol (**4b**).

4,10-*Bis*((6-(4-methoxyphenyl)-quinolin-2-yl)-methyl)-1,4,7,10-tetraazacyclododecane-1,7-diyl)-diacetate (**5b**)

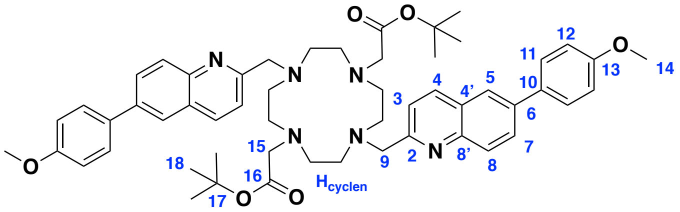

To a solution of DO2A-*tert*-butyl ester (85 mg, 0.21 mmol) and potassium carbonate (88 mg, 0.63 mmol) in anhydrous acetonitrile (8 mL), was added 6-(4-methoxyphenyl)-2-methanesulfonate-2-quinolinemethanol (218 mg, 0.63 mmol). The reaction mixture was stirred at 60 °C for 18 hours. The reaction was cooled to room temperature, salts removed through centrifugation (1500 rpm for 5 minutes). The organic layer was removed, and the salts washed with acetonitrile (2 x 10 mL). The organic layers combined, and the solvent removed under reduced pressure. The crude material was purified by column chromatography (silica gel; neat dichloromethane to 98:2 dichloromethane/methanol) to give the desired protected ligand, as a yellow solid (0.10 g, 54%).

^{1}H NMR (500 MHz, $CDCl_3$): δ 8.23 (2H, d, *J* = 8.2 Hz, H^4), 7.97 – 7.94 (4H, m, H^8, H^5), 7.50 – 7.47 (6H, m, H^{11}, H^7), 7.39 (2H, d, *J* = 8.5 Hz, H^3), 6.94 (4H, d, *J* = 8.8 Hz, H^{12}), 3.97 (4H, br s, H^9), 3.85 (6H, s, H^{14}), 3.45 – 2.54 (20H, m, H^{cyclen}, H^{15}), 1.15 (18H, s, H^{18}). ^{13}C NMR (126 MHz, $CDCl_3$): δ 171.9 (C^{16}), 159.6 (C^{13}), 159.0 (C^2), 147.0 ($C^{8'}$), 138.6 (C^6), 137.3 (C^4), 132.4 (C^{10}), 130.0 (C^8), 129.1 (C^7), 128.4 (C^{11}), 127.7 ($C^{4'}$), 124.5 (C^5), 122.1 (C^3), 114.5 (C^{12}), 82.1 (C^{17}), 60.2 (C^9), 57.9 (C^{15}), 55.5 (C^{14}), 51.0 (C^{cyclen}), 50.7 (C^{cyclen}) 28.1 (C^{18}). ESI-MS (*m*/*z)*: Found $[M + H]^+$ 895.5121, calc $[C_{54}H_{66}N_6O_6 + H]^+$ 985.5117; Found $[M + 2H]^{2+}$ 448.2595, calc $[C_{54}H_{66}N_6O_6 + 2H]^{2+}$ 448.2595.

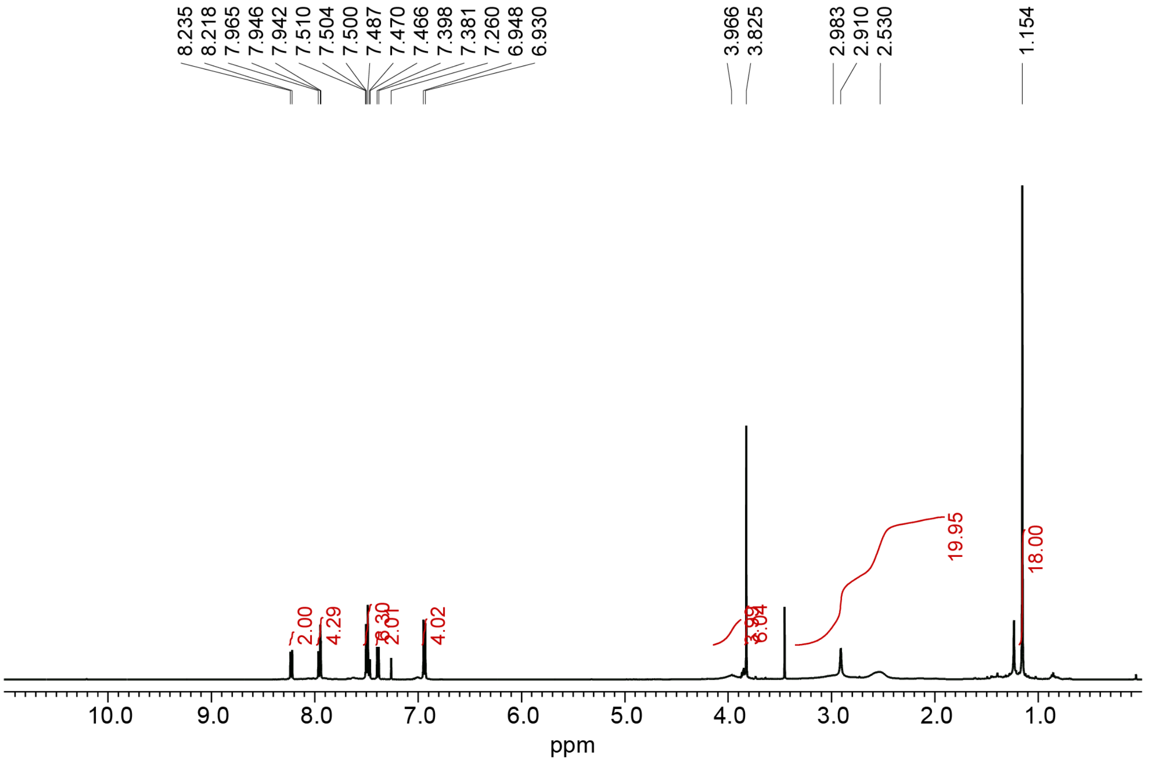

^{1}H NMR spectrum (500 MHz, $CDCl_3$, 298 K) of 4,10-*bis*((6-(4-methoxyphenyl)-quinolin-2-yl)-methyl)-1,4,7,10-tetraazacyclododecane-1,7-diyl)-diacetate (**5b**).

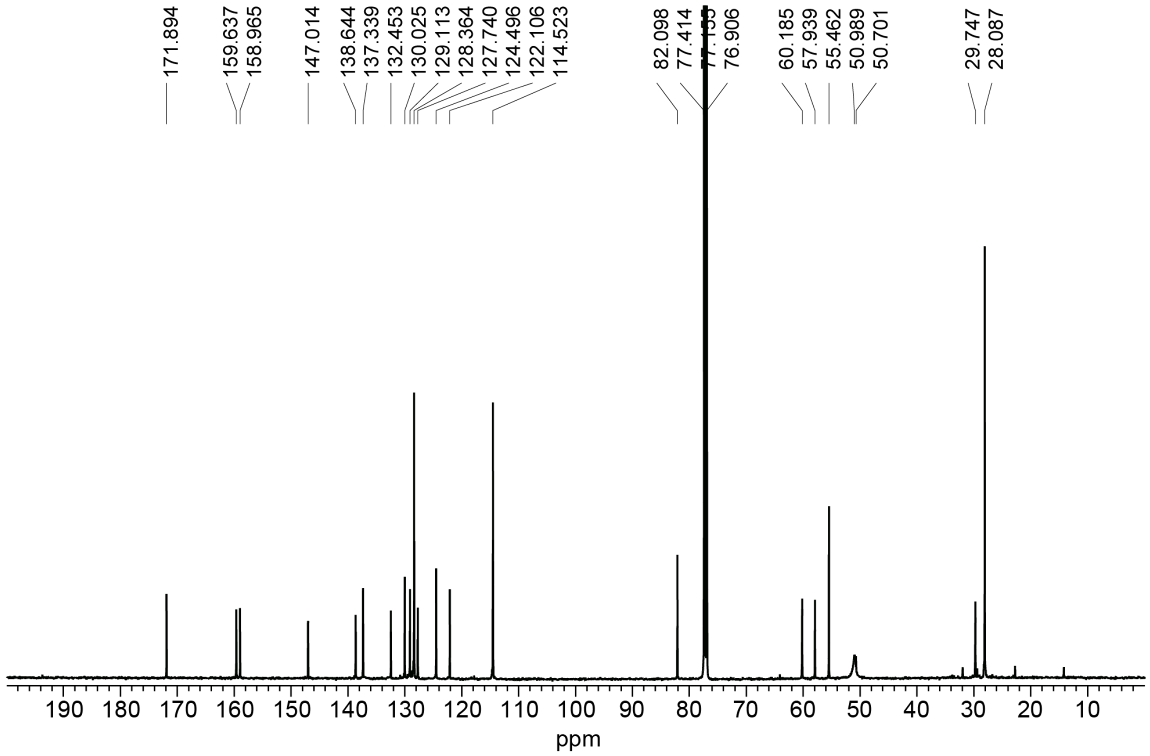

^{13}C NMR spectrum (126 MHz, $CDCl_3$, 298 K) of 4,10-*bis*((6-(4-methoxyphenyl)-quinolin-2-yl)-methyl)-1,4,7,10-tetraazacyclododecane-1,7-diyl)-diacetate (**5b**).

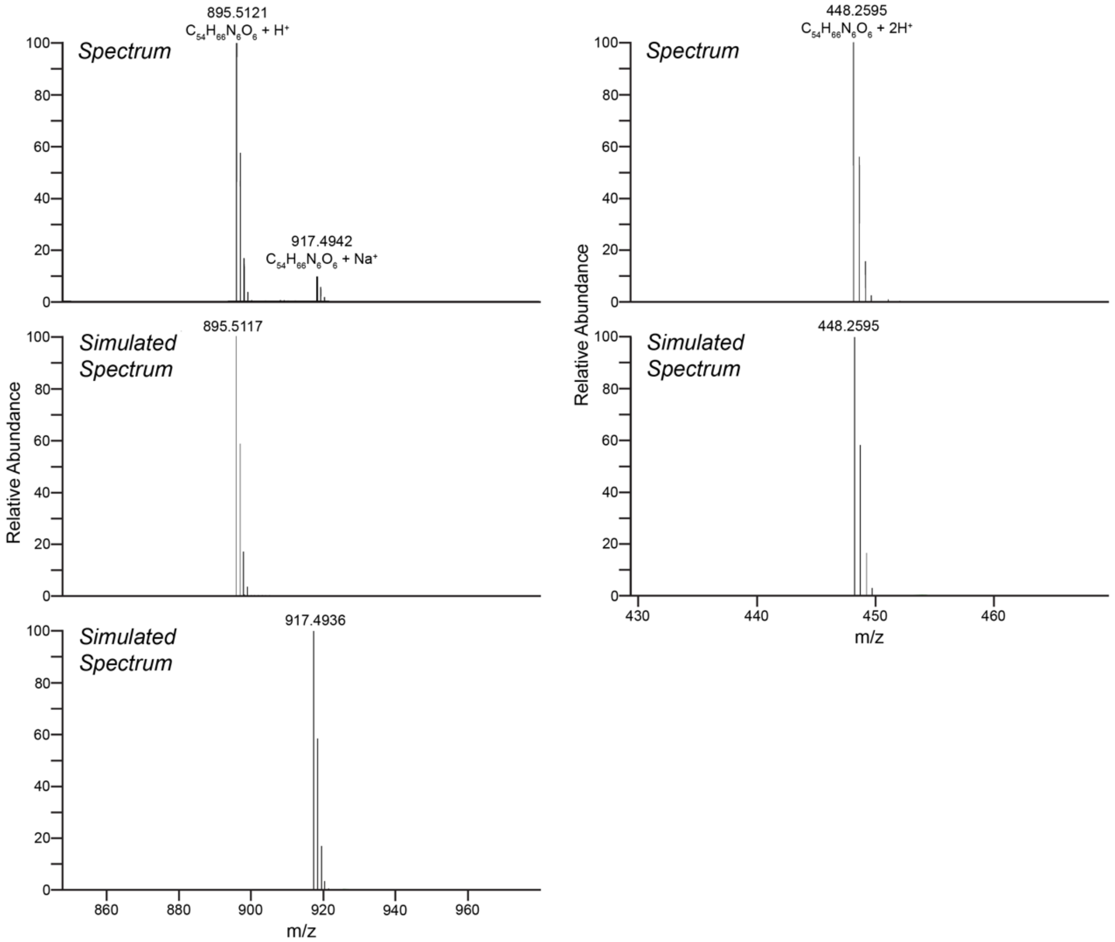

High-resolution mass spectra of 4,10-*bis*((6-(4-methoxyphenyl)-quinolin-2-yl)-methyl)-1,4,7,10-tetraazacyclododecane-1,7-diyl)-diacetate (**5b**).

4,10-*Bis*((6-(4-methoxyphenyl)-quinolin-2-yl)-methyl)-1,4,7,10-tetraazacyclododecane-1,7-diyl)-diacetic acid (**6b**)

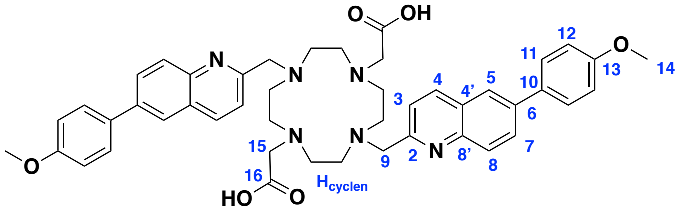

To a solution of 4,10-*bis*((6-(4-methoxyphenyl)-quinolin-2-yl)-methyl)-1,4,7,10-tetraazacyclododecane-1,7-diyl)-diacetate (58 mg, 65 μmol) in dichloromethane (1 mL) was added trifluoroacetic acid (1 mL). The reaction mixture was stirred at room temperature for 6 hours and the trifluoroacetic acid was co-evaporated with dichloromethane (5 x 25 mL) to give the deprotected ligand, isolated as the TFA salt, as a yellow solid (55.3 mg, 84%).

^{1}H NMR (500 MHz, CD_3OD): δ 8.46 (2H, d, *J* = 8.2 Hz, H^4), 8.13 (2H, d, *J* = 1.9 Hz, H^5), 7.98 (2H, d, *J* = 8.8 Hz, H^8), 7.52 – 7.48 (6H, m, H^3, H^{11}), 7.32 (2H, dd, *J* = 8.7 Hz, 2.1 Hz, H^7), 6.85 (4H, d, *J* = 8.5 Hz, H^{12}), 5.02 (4H, s, H^9), 3.87 – 3.78 (18H, m, H^{cyclen}, H^{14}, H^{15}), 3.90 – 3.27 (8H, m, H^{cyclen}). ^{13}C NMR (126 MHz, CD_3OD): δ 172.4 (C^{16}), 159.9 (C^{13}), 151.1 (C^2), 145.6 ($C^{8'}$), 139.4 (C^6), 138.4 (C^4), 131.7 (C^{10}), 129.5 (C^7), 129.2 (C^8), 128.5 ($C^{4'}$), 128.0 (C^{11}), 124.0 (C^5), 119.8 (C^3), 114.2 (C^{12}), 58.7 (C^9), 56.3 (C^{cyclen}), 56.1 (C^{cyclen}), 54.4 (C^{14}), 52.7 (C^{15}), 52.3 (C^{cyclen}), 48.0 (C^{cyclen}). ESI-MS (*m/z)*: Found [M + H]$^+$ 783.3863, calc [$C_{46}H_{50}N_6O_6$ + H]$^+$ 783.3865; Found [M + Na]$^+$ 805.3683, calc [$C_{46}H_{50}N_6O_6$ + Na]$^+$ 805.3684; Found [M + 2H]$^{2+}$ 392.1967, calc [$C_{46}H_{50}N_6O_6$ + 2H]$^{2+}$ 392.1969.

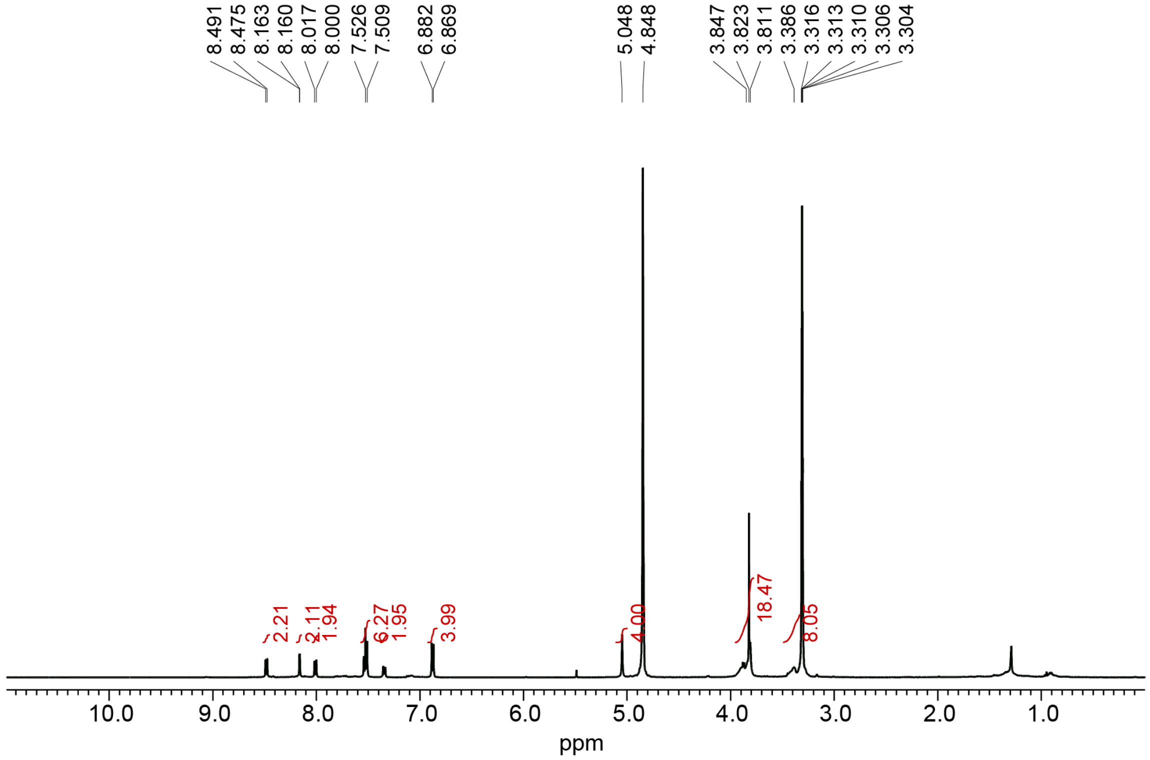

^{1}H NMR spectrum (500 MHz, CD_3OD, 298 K) of 4,10-*bis*((6-(4-methoxyphenyl)-quinolin-2-yl)-methyl)-1,4,7,10-tetraazacyclododecane-1,7-diyl)-diacetic acid (**6b**).

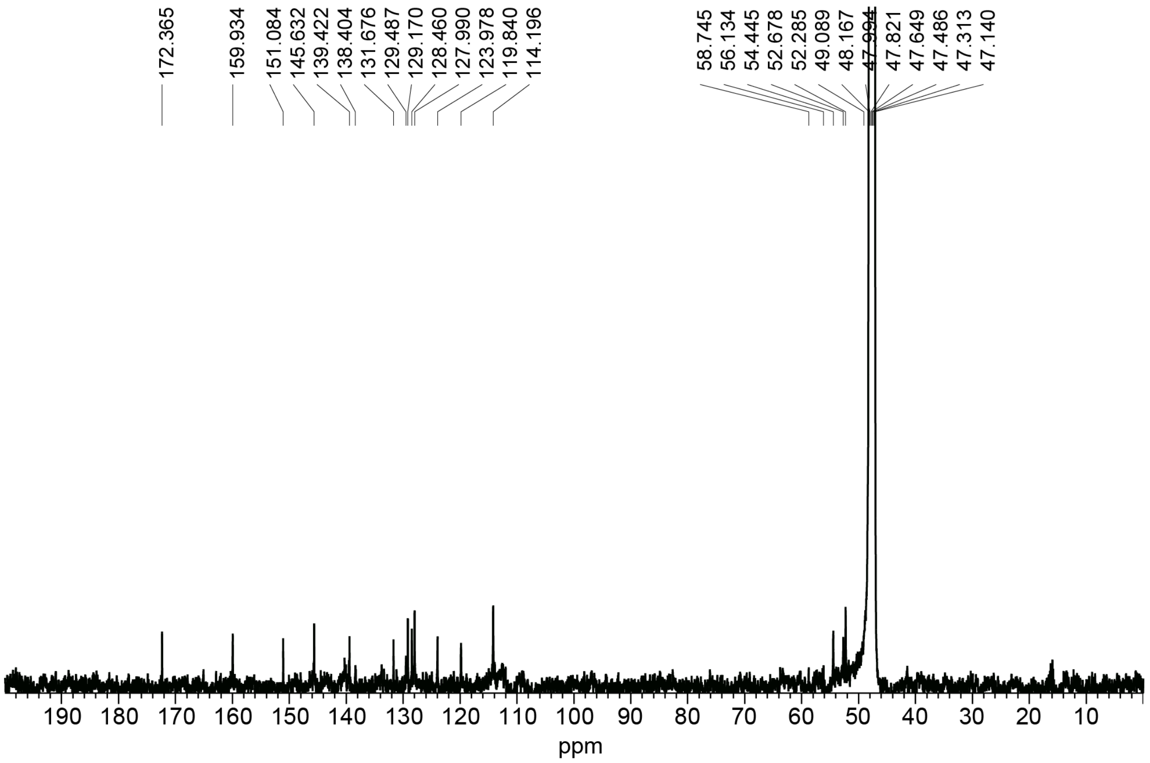

^{13}C NMR spectrum (126 MHz, CD_3OD, 298 K) of 4,10-*bis*((6-(4-methoxyphenyl)-quinolin-2-yl)-methyl)-1,4,7,10-tetraazacyclododecane-1,7-diyl)-diacetic acid (**6b**).

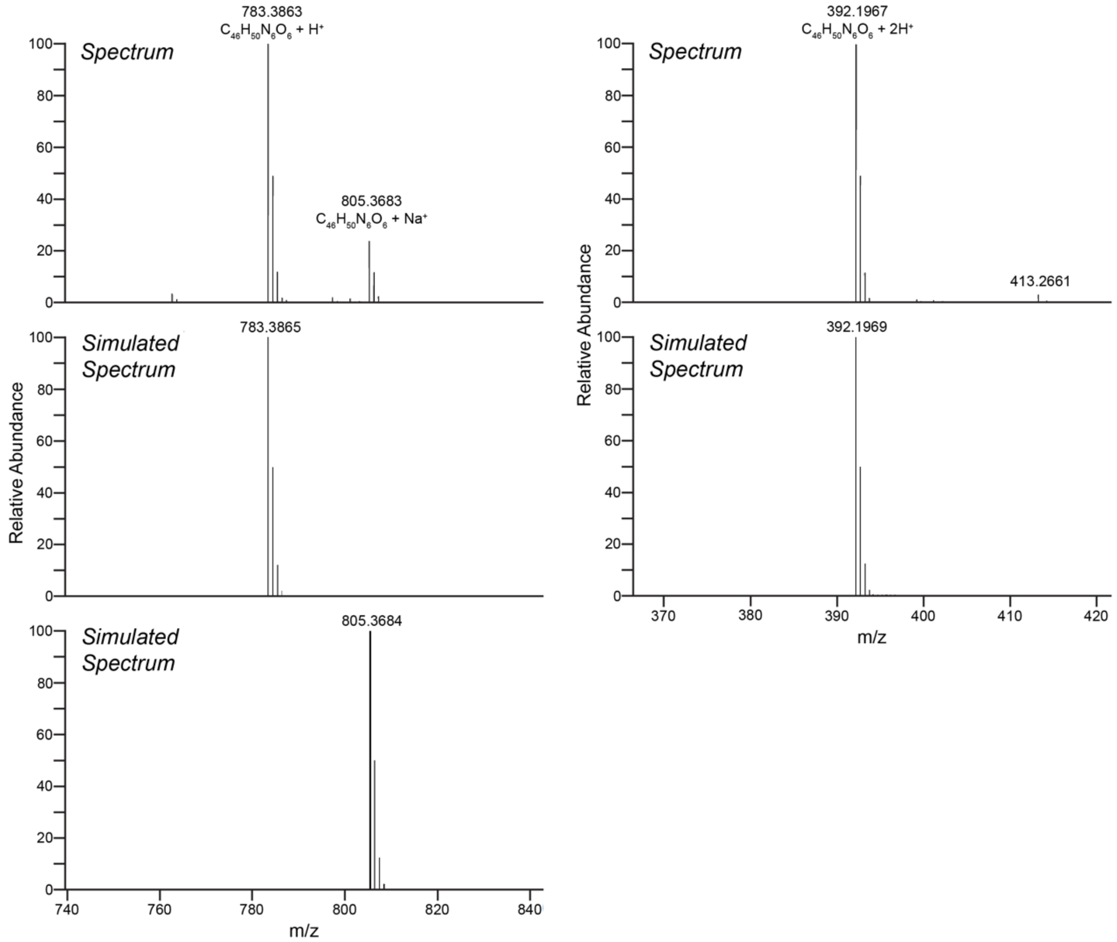

High-resolution mass spectra of 4,10-*bis*((6-(4-methoxyphenyl)-quinolin-2-yl)-methyl)-1,4,7,10-tetraazacyclododecane-1,7-diyl)-diacetic acid (**6b**).

[Eu.6PhOMe]+

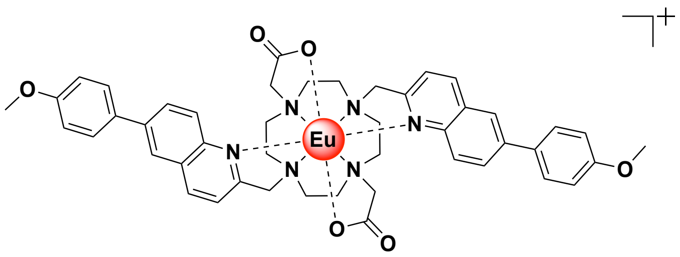

The deprotected ligand (29 mg, 0.03 mmol) was dissolved in methanol (2 mL). Potassium carbonate (2 mg, 0.02 mmol) and europium(III) trifluoromethanesulfonate (50 mg, 0.08 mmol) were added and the reaction heated to 50 °C for 48 hours. The reaction was cooled to room temperature, filtered and the solvent evaporated under reduced pressure. The crude material was purified by column chromatography (silica gel; neat dichloromethane to 9:1 dichloromethane/methanol) to give the desired complex as a pale-yellow solid (19 mg, 63%).

^{1}H NMR spectrum (500 MHz, CD_3OD): δ 51.8, 30.4, 17.6, 17.4, 15.7, 15.4, 13.7, 13.0, 12.0, 11.4, 8.5, 8.3, 7.7, 7.5, 7.3, 7.2, 6.6, 6.2, 5.8, 5.5, 5.3, 4.9, 4.8, 4.1, 3.9, 3.6, 3.4, 3.3, 3.1, 3.0, 2.8, 2.0, 1.8, 1.6, 1.3, 1.1, 0.9, 0.5, 0.1, -0.4, -2.0, -2.9, -5.7, -9.5, -11.1, -11.3, -16.5, -16.9, -17.7, -28.5, -29.6, -30.4, -34.2. ESI-MS (*m/z)*: Found $[M]^+$ 933.2844, calc $[C_{46}H_{48}EuN_6O_6]^+$ 933.2842. Photophysical data measured in methanol: λ_{max} = 340 nm, ε = 8000 M^{-1} cm^{-1}, Φ_{em} = 1.0%, τ_{CH3OH}= 0.88 ms, τ_{CD3OD} = 1.16 ms, *m* = 0.7.

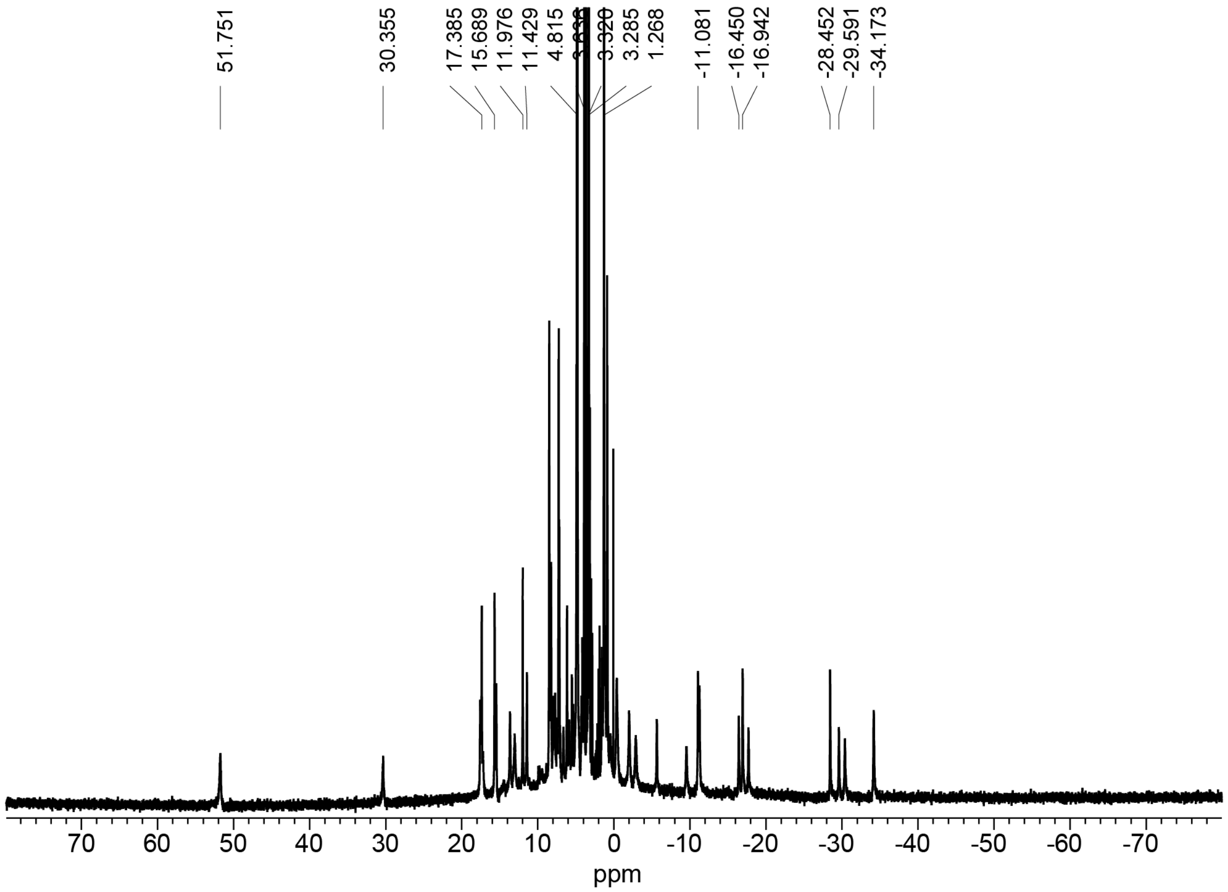

^{1}H NMR spectrum (500 MHz, CD_3OD, 298 K) of [**Eu.6PhOMe**]+.

[Gd.6PhOMe]+

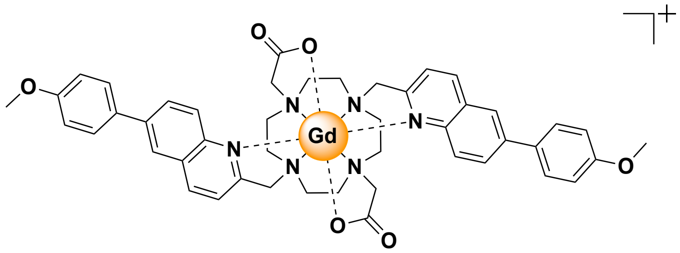

The deprotected ligand (20 mg, 0.03 mmol) was dissolved in methanol (2 mL) then potassium carbonate (7 mg, 0.05 mmol) and gadolinium(III) trifluoromethanesulfonate (31 mg, 0.05 mmol) were added and the reaction heated to 60 °C for 24 hours. The reaction was cooled to room temperature, filtered and the solvent evaporated under reduced pressure. The crude material was purified by column chromatography (silica gel; neat dichloromethane to 4:1 dichloromethane/methanol) to give the desired complex as a white solid (23 mg, 82%).

ESI-MS (*m/z*): Found $[M]^+$ 938.2878, calc $[C_{46}H_{48}GdN_6O_6]^+$ 938.2871. Photophysical data measured in methanol: λ_{max} = 342 nm.

4-(2-Methyl-6-quinolinyl)-phenol (**1c**)

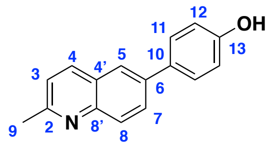

6-Bromoquinaldine (1.00 g, 4.54 mmol), 4-hydroxyphenyl boronic acid (0.94 g, 6.82 mmol) and potassium carbonate (1.26 g, 9.09 mmol) were added to an oven-dried Schlenk with a condenser attached under a nitrogen atmosphere. Anhydrous dioxane (30 mL) and oxygen-free water (8 mL) were added, followed by palladium-tetrakis(triphenylphosphine) (0.26 g, 0.23 mmol) and the reaction was heated to 60 °C for 18 hours. The reaction as cooled to room temperature, filtered through celite and the filtrate was removed under reduced pressure. The residue was taken up in ethyl acetate (100 mL) and the off-white solid collected through vacuum filtration (0.80 g, 75%).

^{1}H NMR spectrum (500 MHz, DMSO-d_6): δ 8.24 (1H, d, J = 8.5 Hz, H^4), 8.07 (1H, s, H^5), 7.97 – 7.91 (2H, m, H^7, H^8), 7.61 (2H, d, J = 8.5 Hz, H^{11}), 7.40 (1H, d, J = 8.2 Hz, H^3), 6.86 (2H, d, J = 8.2 Hz, H^{12}), 2.65 (3H, s, H^9). ^{13}C NMR (126 MHz, DMSO-d_6): δ 159.3* (C^{13}), 158.6 (C^2), 146.7 ($C^{8'}$), 138.0 (C^6), 136.6 (C^4), 129.5 (C^{10}), 129.0 (C^8), 128.6 (C^{11}), 128.5 (C^7), 127.1 ($C^{4'}$), 124.0 (C^5), 122.9 (C^3), 116.8 (C^{12}), 25.3 (C^9). ESI-MS (m/z): Found $[M + H]^+$ 236.1070, calc $[C_{16}H_{13}NO + H]^+$ 236.1070.

*Assigned through HMBC

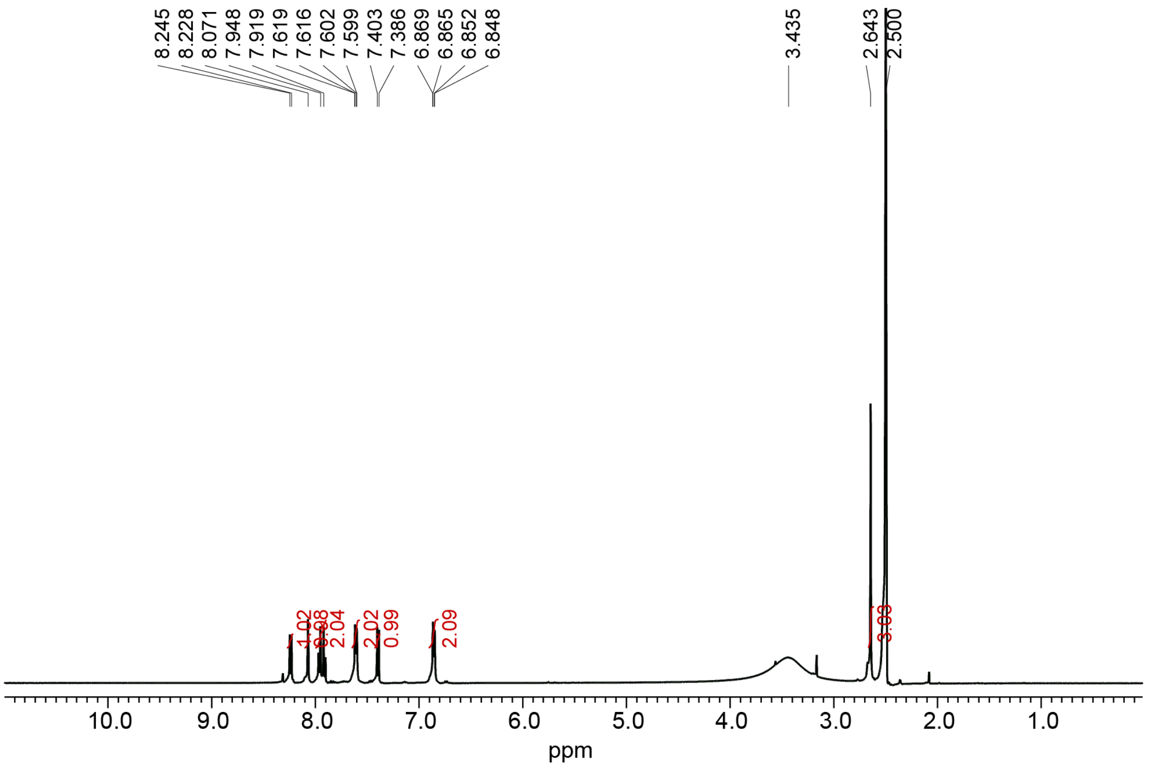

^{1}H NMR spectrum (500 MHz, DMSO-d_6, 298 K) of 4-(2-methyl-6-quinolinyl)-phenol (**1c**).

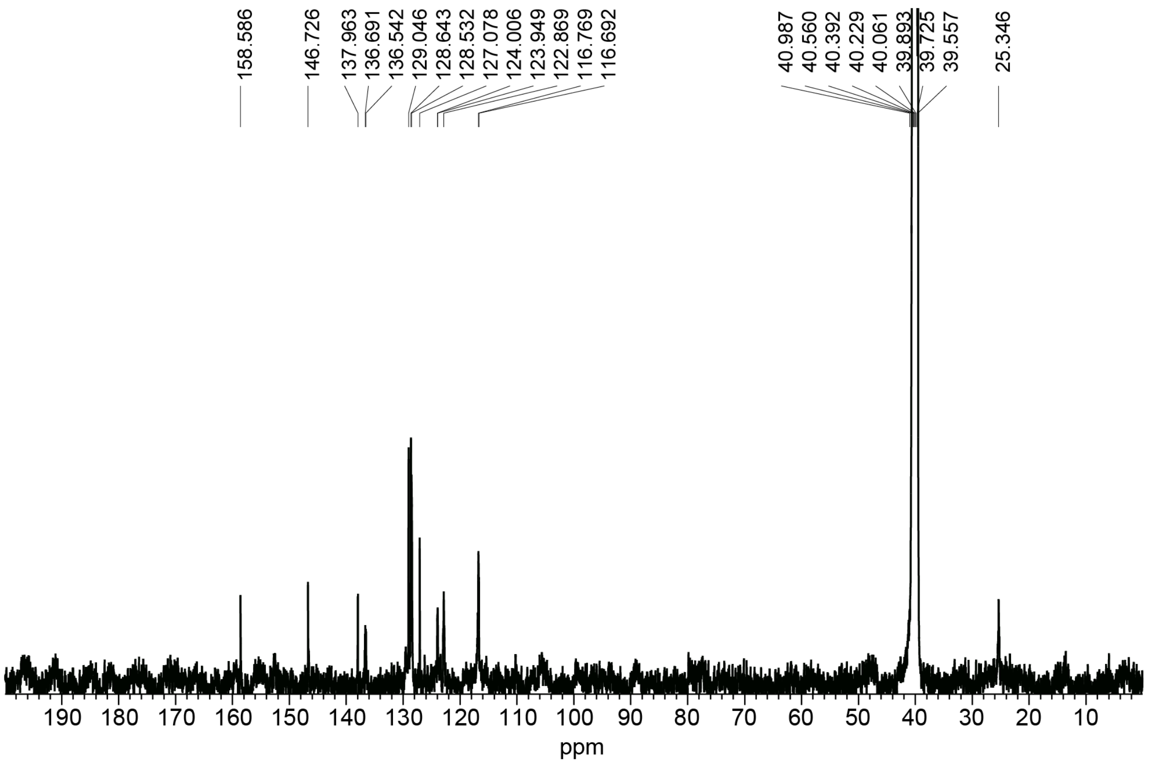

^{13}C NMR spectrum (126 MHz, DMSO-d_6, 298 K) of 4-(2-methyl-6-quinolinyl)-phenol (**1c**).

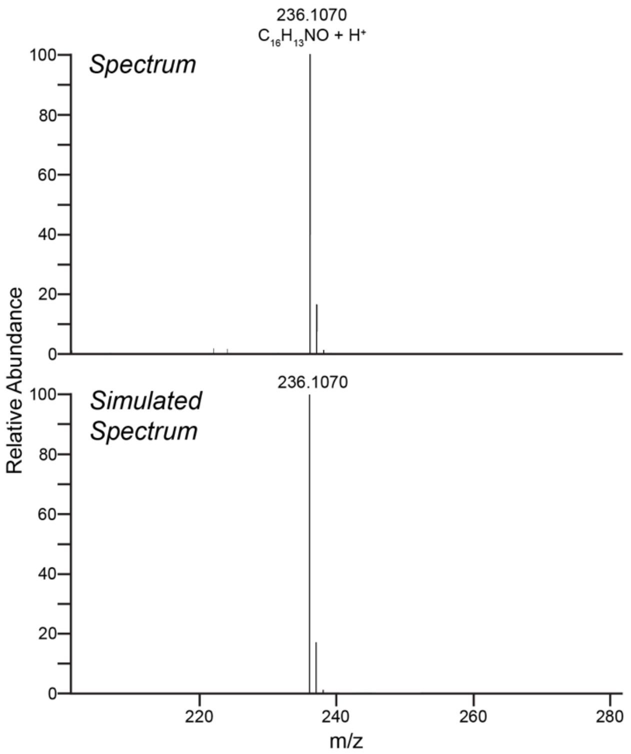

High-resolution mass spectra of 4-(2-methyl-6-quinolinyl)-phenol (**1c**).

6-(4-(Phenoxy)*tert*-butyl acetate)-2-methylquinoline or 6-(4-(1,1-dimethylethyl)-phenoxyacetate)-2-methylquinoline (**1d**)

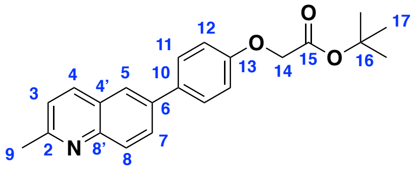

Under a nitrogen atmosphere, 4-(2-methyl-6-quinolinyl)-phenol (0.60 g, 2.54 mmol) and potassium carbonate (0.42 g, 3.05 mmol) were dissolved in anhydrous dimethylformamide (10 mL). Subsequently, *tert*-butyl chloroacetate (0.77 g, 5.08 mmol) was added and reaction stirred at room temperature for 8 hours. The reaction mixture was filtered, and solvent was evaporated under reduced pressure. The residue was taken up in water (50 mL) then extracted with dichloromethane (3 x 50 mL). The organic layers were combined, dried ($MgSO_4$) and solvent evaporated under reduced pressure to give the pure product as a white solid (0.60 g, 67%).

^{1}H NMR spectrum (500 MHz, $CDCl_3$): δ 8.08 – 8.04 (2H, m, H^4, H^8), 7.91 – 7.90 (2H, m, H^7, H^5), 7.64 (2H, d, J = 8.8 Hz, H^{11}), 7.30 (1H, d, J = 8.2 Hz, H^3), 7.02 (2H, d, J = 8.8 Hz, H^{12}), 4.58 (2H, s, H^{14}), 2.76 (3H, s, H^9), 1.51 (9H, s, H^{17}). ^{13}C NMR (126 MHz, $CDCl_3$): δ 168.0 (C^{15}), 158.9 (C^2), 157.8 (C^{13}), 147.1 ($C^{8'}$), 138.0 (C^6), 136.3 (C^4), 133.9 (C^{10}), 129.1 (C^7), 129.0 (C^8), 128.5 (C^{11}), 126.8 ($C^{4'}$), 124.7 (C^5), 122.4 (C^3), 115.1 (C^{12}), 82.6 (C^{16}), 65.9 (C^{14}), 28.1 (C^{17}), 25.5 (C^9). ESI-MS (*m/z*): Found $[M + H]^+$ 350.1751, calc $[C_{22}H_{23}NO_3 + H]^+$ 350.1751; Found $[M + Na]^+$ 372.1571, calc $[C_{22}H_{23}NO_3 + Na]^+$ 372.1570.

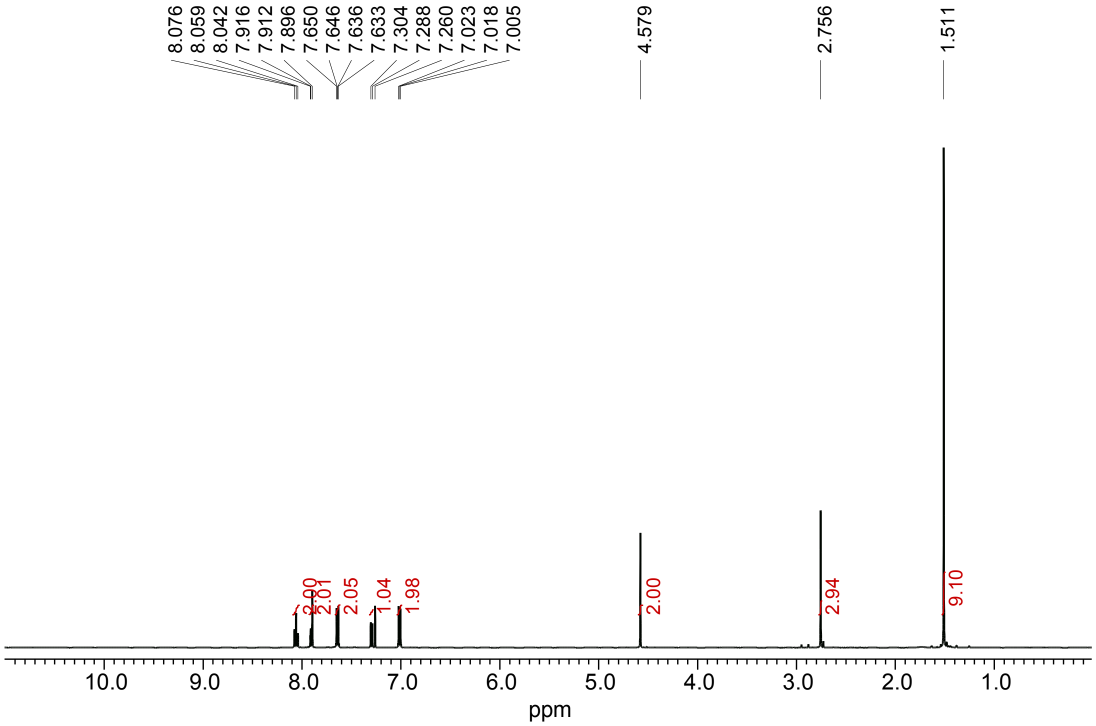

^{1}H NMR spectrum (500 MHz, $CDCl_3$, 298 K) of 6-(4-(phenoxy)*tert*-butyl acetate)-2-methylquinoline or 6-(4-(1,1-dimethylethyl)-phenoxyacetate)-2-methylquinoline (**1d**).

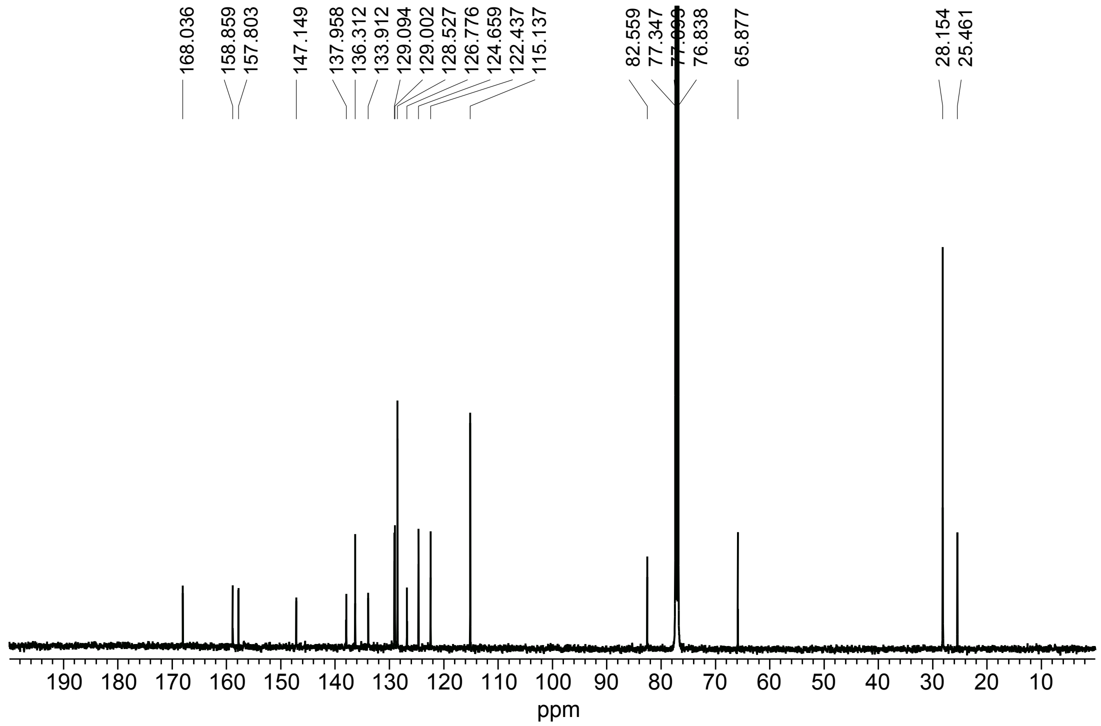

^{13}C NMR spectrum (126 MHz, $CDCl_3$, 298 K) of 6-(4-(phenoxy)*tert*-butyl acetate)-2-methylquinoline or 6-(4-(1,1-dimethylethyl)-phenoxyacetate)-2-methylquinoline (**1d**).

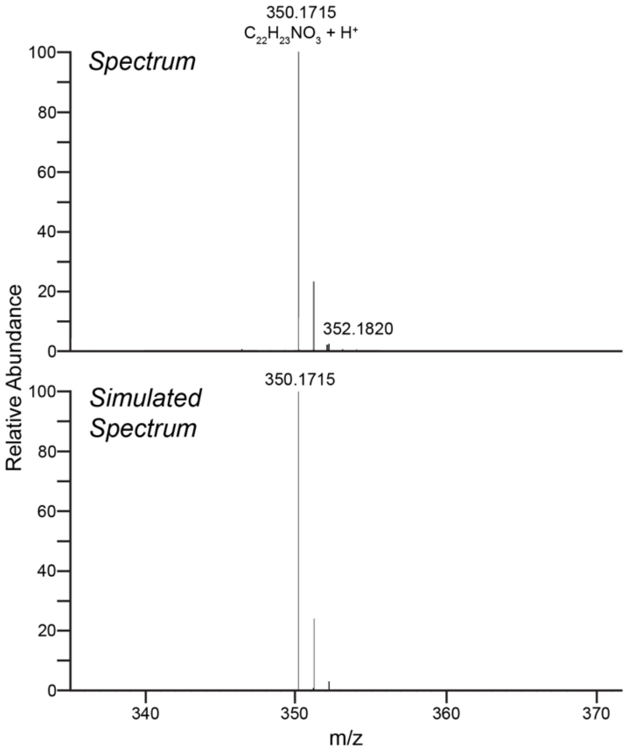

High-resolution mass spectra of 6-(4-(phenoxy)*tert*-butyl acetate)-2-methylquinoline or 6-(4-(1,1-dimethylethyl)-phenoxyacetate)-2-methylquinoline (**1d**).

6-(4-(1,1-Dimethylethyl)-phenoxyacetate)-2-quinolinecarboxaldehyde (**2c**)

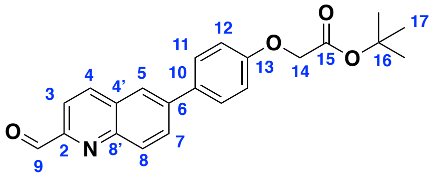

6-(4-(1,1-Dimethylethyl)-phenoxyacetate)-2-methylquinoline (0.25 g, 0.71 mmol) and anhydrous dioxane (15 mL) were added to oven-dried glassware under a nitrogen atmosphere. Selenium dioxide (0.21 g, 1.87 mmol) was added as one solid portion, and the reaction was heated to 60 °C for 18 hours. The reaction was cooled to room temperature then brine (30 mL) and ethyl acetate (50 mL) were added. The biphasic mixture was passed through a celite plug then the organic layer was separated. The aqueous layer was extracted with ethyl acetate (2 x 50 mL), organic layers combined and washed with brine (100 mL), dried ($MgSO_4$) and the solvent removed under reduced pressure to obtain pure product (0.24 g, 92%).

^{1}H NMR spectrum (500 MHz, $CDCl_3$): δ 10.23 (1H, s, H^9), 8.32 (1H, d, J = 8.5 Hz, H^4), 8.28 (1H, d, J = 8.8 Hz, H^8), 8.07 – 8.02 (3H, m, H^7, H^3, H^5), 7.69 (2H, d, J = 8.8 Hz, H^{11}), 7.04 (2H, d, J = 8.8 Hz, H^{12}), 4.60 (2H, s, H^{14}), 1.52 (9H, s, H^{17}). ^{13}C NMR (126 MHz, $CDCl_3$): δ 193.7 (C^9), 167.9 (C^{15}), 158.4 (C^{13}), 152.4 (C^2), 147.2 ($C^{8'}$), 141.5 (C^6), 137.4 (C^4), 133.0 (C^{10}), 130.9 (C^8), 130.5 ($C^{4'}$), 130.2 (C^7), 128.8 (C^{11}), 124.7 (C^5), 117.9 (C^3), 115.3 (C^{12}), 82.7 (C^{16}), 65.8 (C^{14}), 28.2 (C^{17}). ESI-MS (*m/z)*: Found $[M + H]^+$ 364.1542, calc $[C_{22}H_{21}NO_4 + H]^+$ 364.1543.

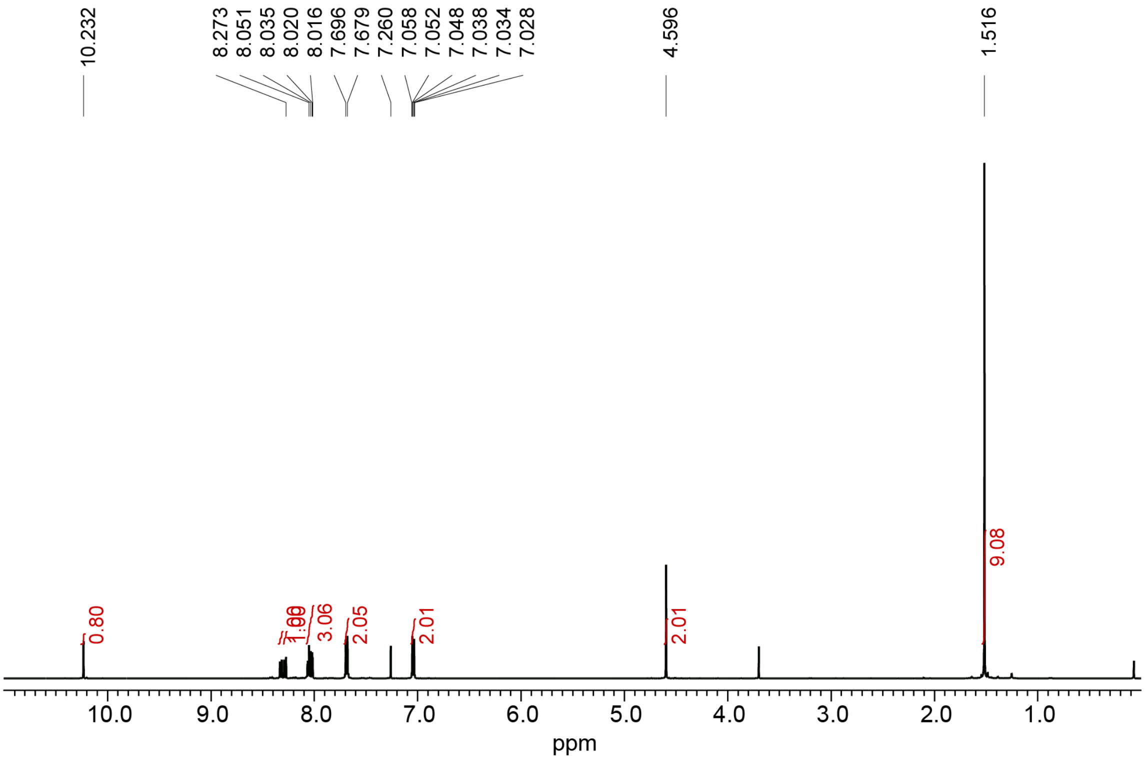

^{1}H NMR spectrum (500 MHz, $CDCl_3$, 298 K) of 6-(4-(1,1-dimethylethyl)-phenoxyacetate)-2-quinolinecarboxaldehyde (**2c**).

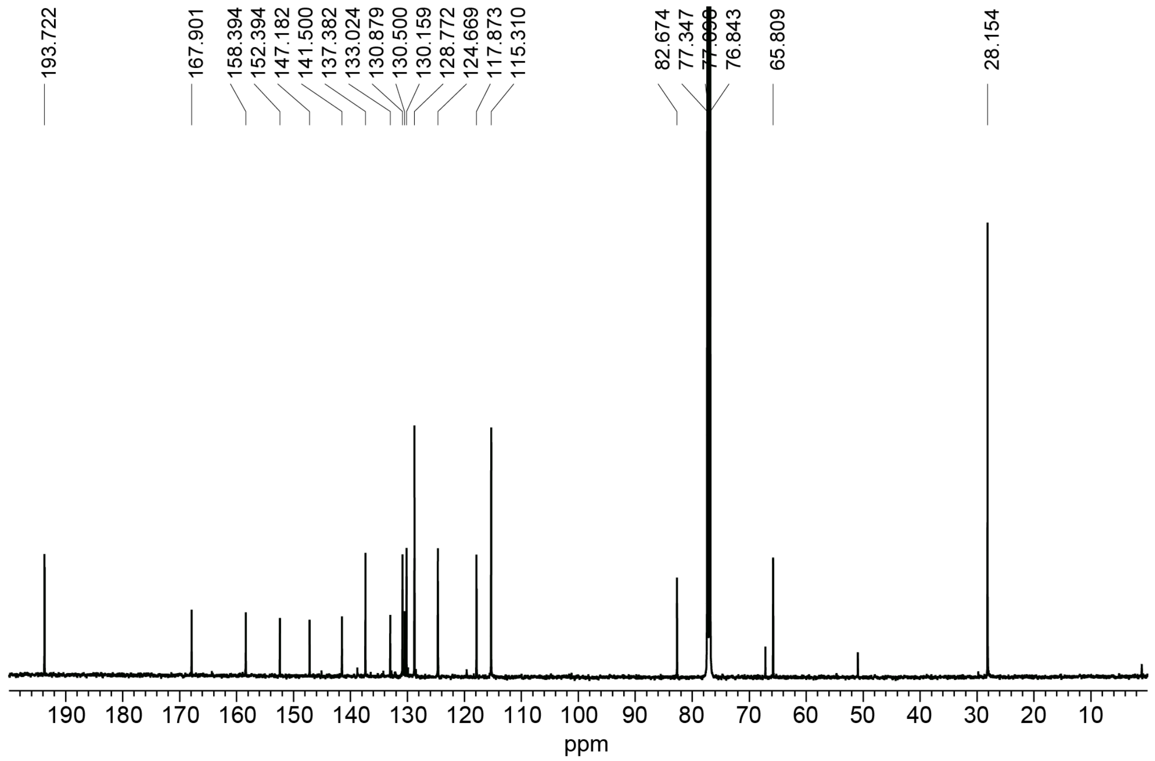

^{13}C NMR spectrum (126 MHz, $CDCl_3$, 298 K) of 6-(4-(1,1-dimethylethyl)-phenoxyacetate)-2-quinolinecarboxaldehyde (**2c**).

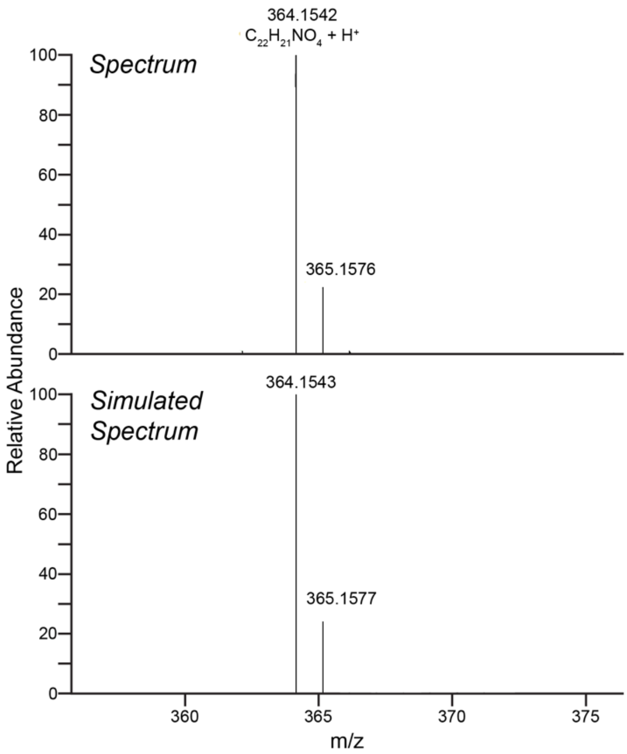

High-resolution mass spectra of 6-(4-(1,1-dimethylethyl)-phenoxyacetate)-2-quinolinecarboxaldehyde (**2c**).

6-(4-(1,1-Dimethylethyl)-phenoxyacetate)-2-quinolinemethanol (**3c**)

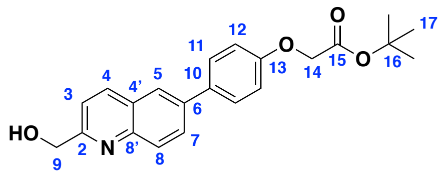

6-(4-(1,1-Dimethylethyl)-phenoxyacetate)-2-quinolinecarboxaldehyde (0.21 g, 0.55 mmol) was dissolved in anhydrous methanol (15 mL) under a nitrogen atmosphere. The flask was cooled to 0 °C, sodium borohydride (0.03 g, 0.66 mmol) was carefully added as one solid and the reaction was allowed to warm to room temperature and stirred for 4 hours. The reaction was quenched with saturated NH_4Cl solution (20 mL) then the methanol was evaporated under reduced pressure. The remaining aqueous solution was extracted with chloroform (3 x 50 mL), organics combined, washed with brine (2 x 50 mL), dried ($MgSO_4$) and the solvent evaporated under reduced pressure to yield the product as a yellow solid (0.19 g, 91%).

^{1}H NMR spectrum (500 MHz, $CDCl_3$): δ 8.16 (1H, d, J = 8.5 Hz, H^4), 8.10 (1H, d, J = 9.2 Hz, H^8), 7.95 – 7.94 (2H, m, H^7, H^5), 7.65 (2H, d, J = 8.8 Hz, H^{11}), 7.30 (1H, d, J = 8.2 Hz, H^3), 7.02 (2H, d, J = 8.8 Hz, H^{12}), 4.93 (2H, s, H^9), 4.59 (2H, s, H^{14}), 1.51 (2H, s, H^{17}). ^{13}C NMR (126 MHz, $CDCl_3$): δ 168.0 (C^{15}), 158.9 (C^2), 157.9 (C^{13}), 145.9 ($C^{8'}$), 138.7 (C^6), 137.0 (C^4), 133.6 (C^{10}), 129.4 (C^7), 129.0 (C^8), 128.6 (C^{11}), 127.9 ($C^{4'}$), 124.8 (C^5), 118.8 (C^3), 115.2 (C^{12}), 82.6 (C^{16}), 65.8 (C^9), 64.3 (C^{14}), 28.1 (C^{17}). ESI-MS (*m/z)*: Found $[M + H]^+$ 366.1698, calc $[C_{22}H_{23}NO_4 + H]^+$ 366.1700.

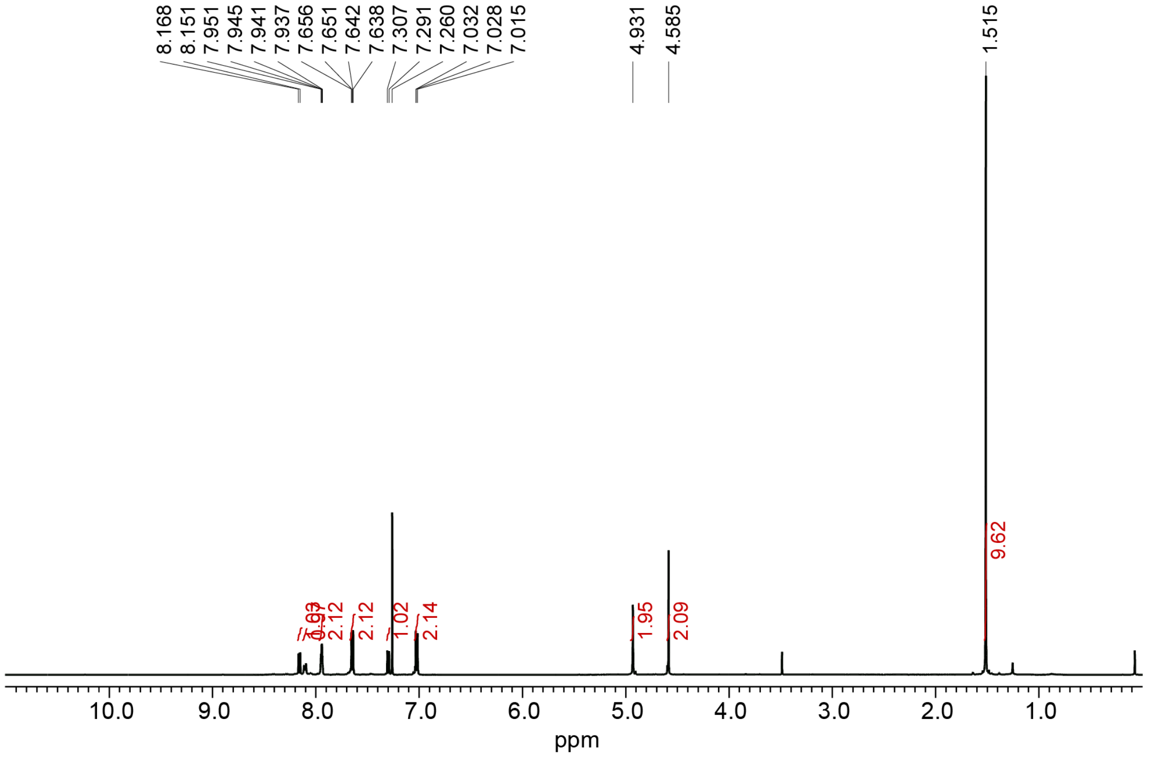

^{1}H NMR spectrum (500 MHz, $CDCl_3$, 298 K) of 6-(4-(1,1-dimethylethyl)-phenoxyacetate)-2-quinolinemethanol (**3c**).

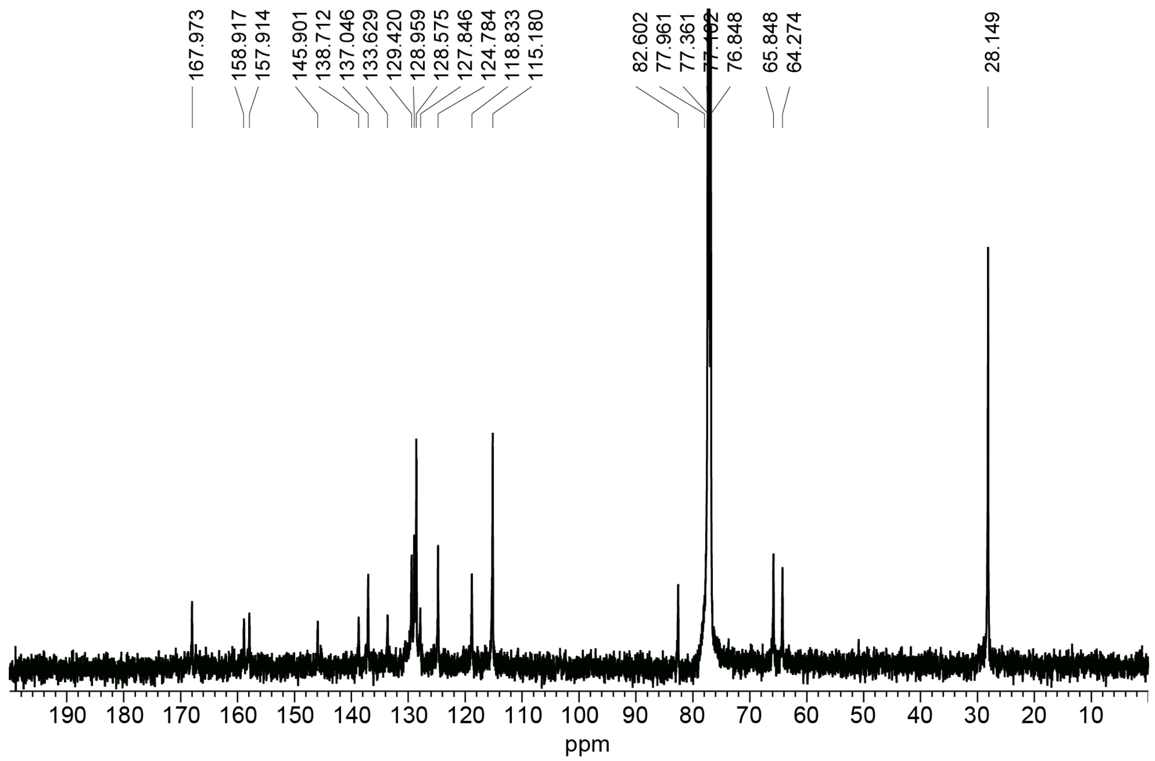

^{13}C NMR spectrum (126 MHz, $CDCl_3$, 298 K) of 6-(4-(1,1-dimethylethyl)-phenoxyacetate)-2-quinolinemethanol (**3c**).

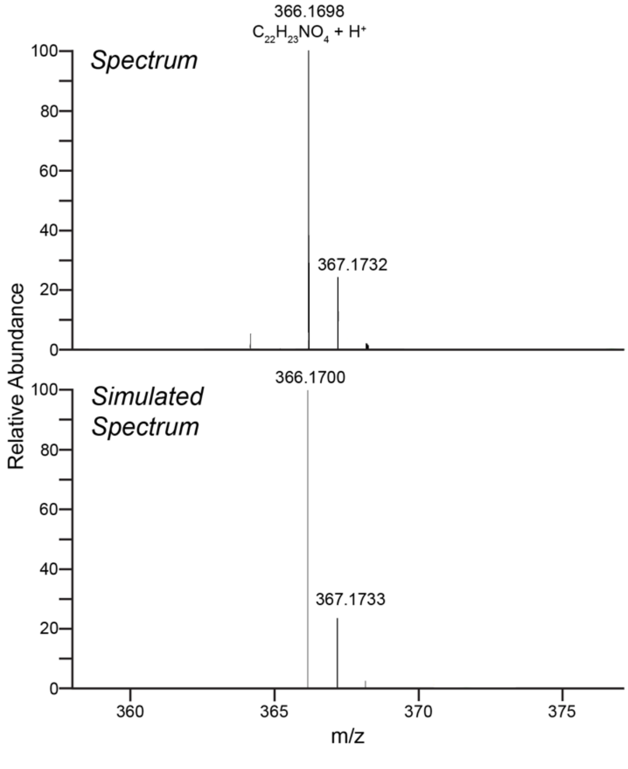

High-resolution mass spectra of 6-(4-(1,1-dimethylethyl)-phenoxyacetate)-2-quinolinemethanol (**3c**).

6-(4-(1,1-Dimethylethyl)-phenoxyacetate)-2-methanesulfonate-2-quinolinemethanol (**4c**)

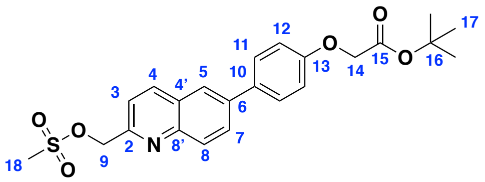

In an oven-dried Schlenk, 6-(4-(1,1-dimethylethyl)-phenoxyacetate)-2-quinolinemethanol (0.18 g, 0.48 mmol) was dissolved in anhydrous dichloromethane (5 mL) under a nitrogen atmosphere. Diisopropylethylamine (0.16 mL, 0.72 mmol) and methanesulfonyl chloride (0.04 mL, 0.47 mmol) were added and the mixture was stirred at room temperature for 4 hours. Water (20 mL) was added, and the organic layer separated. The aqueous layer was extracted with dichloromethane (2 x 15 mL) and the combined organic layers were washed with brine (50 mL), dried ($MgSO_4$) and concentrated under reduced pressure, to give the product as a dark orange oil (0.16, 99%).

^{1}H NMR spectrum (500 MHz, $CDCl_3$): δ 8.26 (1H, d, J = 8.5 Hz, H^4), 8.10 (1H, d, J = 8.5 Hz, H^8), 7.98 – 7.95 (2H, m, H^7, H^5), 7.65 (2H, d, J = 8.5 Hz, H^{11}), 7.60 (1H, d, J = 8.5 Hz, H^3), 7.03 (2H, d, J = 8.5 Hz, H^{12}), 5.51 (2H, s, H^9), 4.59 (2H, s, H^{14}), 3.12 (3H, s, H^{18}), 1.51 (9H, s, H^{17}). ^{13}C NMR (126 MHz, $CDCl_3$): δ 168.0 (C^{15}), 158.1 (C^{13}), 153.6 (C^2), 146.8 ($C^{8'}$), 139.6 (C^6), 137.5 (C^4), 133.4 (C^{10}), 129.8 (C^8), 129.7 (C^7), 128.6 (C^{11}), 128.1 ($C^{4'}$), 124.7 (C^5), 120.1 (C^3), 115.2 (C^{12}), 82.6 (C^{16}), 72.3 (C^9), 65.8 (C^{14}), 38.2 (C^{18}), 28.2 (C^{17}). LR-MS ESI (m/z): Found [M + H]$^+$ 444.1, calc [$C_{23}H_{25}NO_6S$ + H]$^+$ 444.1.

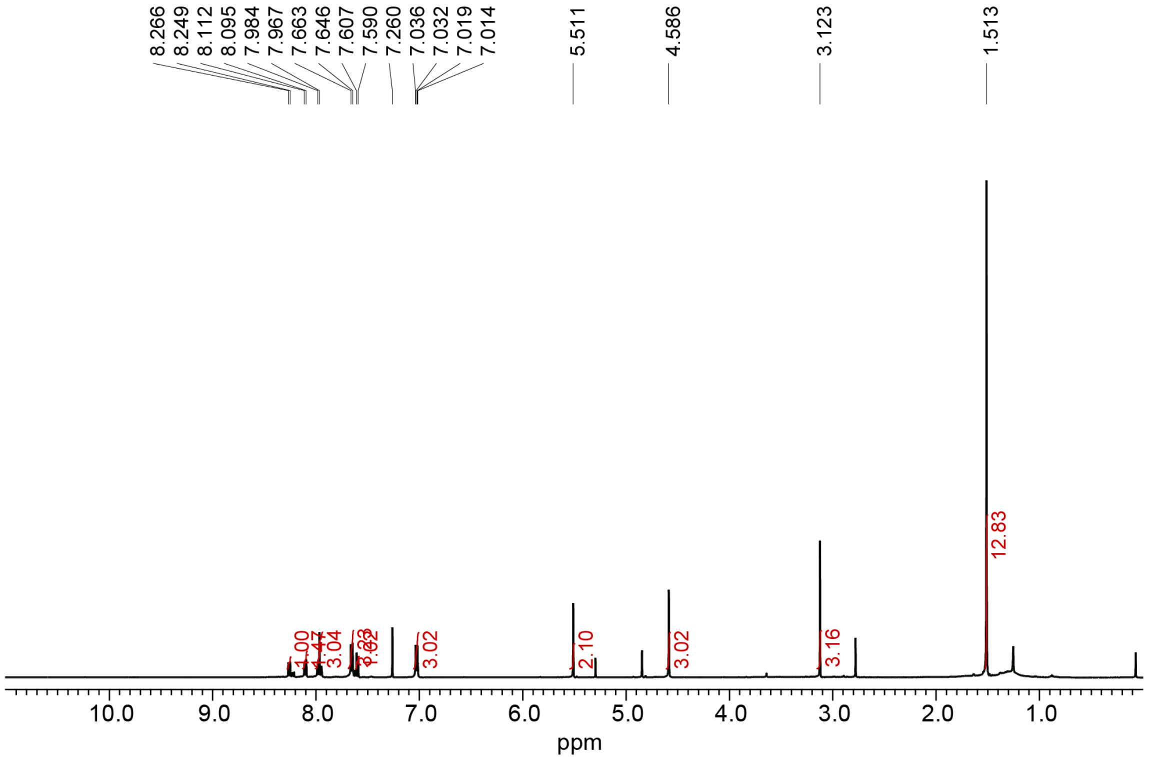

^{1}H NMR spectrum (500 MHz, $CDCl_3$, 298 K) of 6-(4-(1,1-dimethylethyl)-phenoxyacetate)-2-methanesulfonate-2-quinolinemethanol (**4c**).

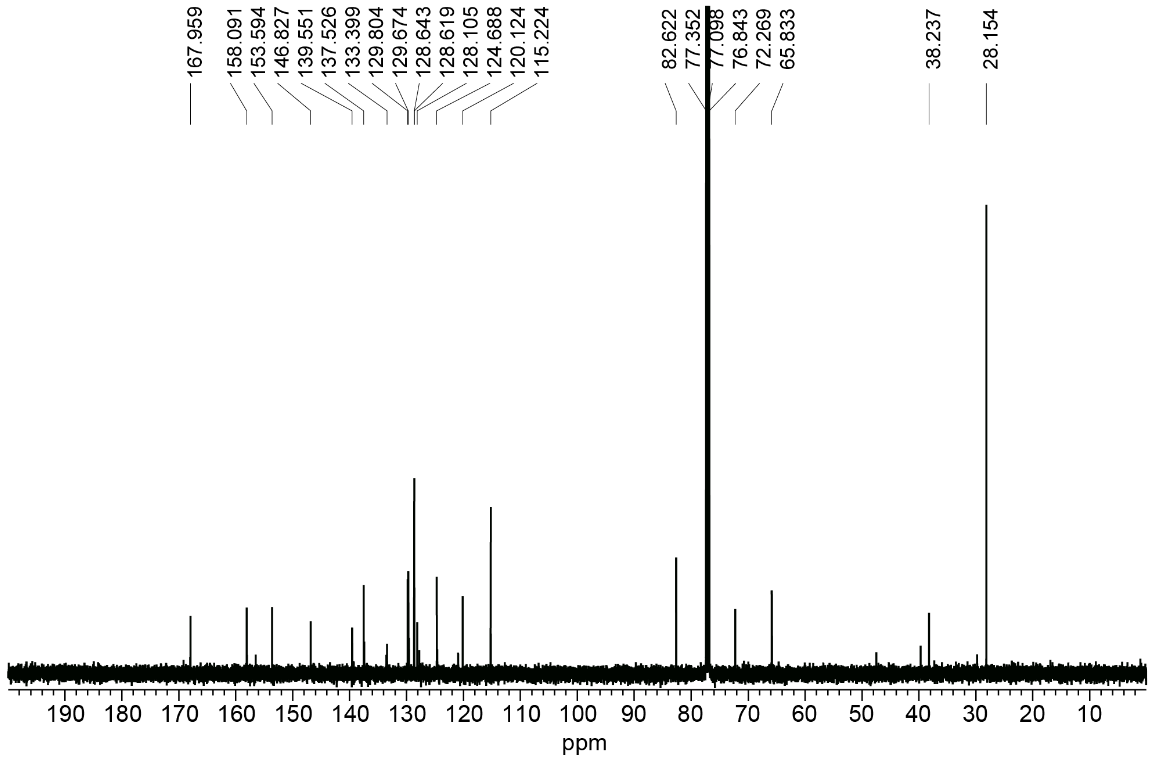

^{13}C NMR spectrum (126 MHz, $CDCl_3$, 298 K) of 6-(4-(1,1-dimethylethyl)-phenoxyacetate)-2-methanesulfonate-2-quinolinemethanol (**4c**).

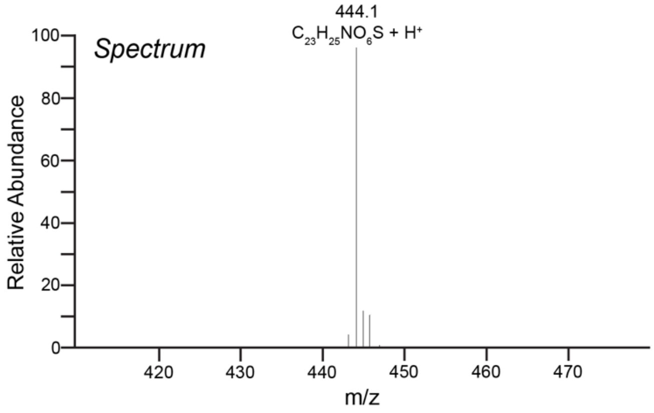

Low-resolution mass spectrum of 6-(4-(1,1-dimethylethyl)-phenoxyacetate)-2-methanesulfonate-2-quinolinemethanol (**4c**).

4,10-*Bis*((6-(4-(1,1-dimethylethyl)-phenoxyacetate)-quinolin-2-yl)-methyl)-1,4,7,10-tetraazacyclododecane-1,7-diyl)-diacetate (**5c**)

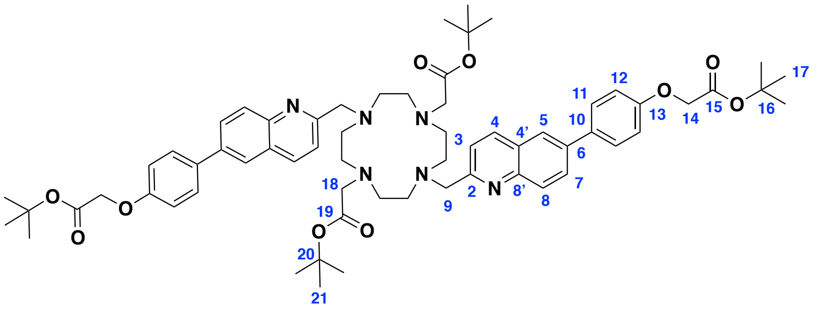

To a solution of DO2A-*tert*-butyl ester (60 mg, 0.15 mmol) and potassium carbonate (62 mg, 0.45 mmol) in anhydrous acetonitrile (8 mL), was added 6-(4-(1,1-dimethylethyl)-phenoxyacetate)-2-methanesulfonate-2-quinolinemethanol (160 mg, 0.36 mmol). The reaction mixture was stirred at 60 °C for 18 hours. The reaction was cooled to room temperature, salts removed through centrifugation (1500 rpm for 5 minutes). The organic layer was removed, and the salts washed with acetonitrile (2 x 10 mL). The organic layers combined, and the solvent removed under reduced pressure. The crude material was purified by column chromatography (silica gel; neat dichloromethane to 95:5 dichloromethane/methanol) to give the desired protected product, as a yellow solid (0.11 g, 69%).

^{1}H NMR spectrum (500 MHz, CD_3OD): δ 8.37 (2H, d, *J* = 8.5 Hz, H^4), 8.09 (2H, d, *J* = 1.9 Hz, H^5), 8.01 (2H, d, *J* = 8.8 Hz, H^8), 7.57 – 7.53 (6H, m, H^{11}, H^7), 7.50 (2H, d, *J* = 8.5 Hz, H^3), 6.97 (4H, d, *J* = 8.8 Hz, H^{12}), 4.62 (4H, s, H^{14}), 3.98 (4H, br s, H^9), 3.01 – 2.55 (20H, m, H^{18}, H^{cyclen}), 2.78 (18H, s, H^{17}), 1.49 (18H, s, H^{21}). ^{13}C NMR (126 MHz, CD_3OD): δ 172.3 (C^{19}), 168.7 (C^{15}), 159.4 (C^{13}), 158.1 (C^2), 146.8 ($C^{8'}$), 138.3 (C^6), 137.2 (C^4), 133.0 (C^{10}), 129.9 (C^8), 128.6 (C^7), 128.0 ($C^{4'}$), 127.9 (C^{11}), 124.3 (C^5), 122.0 (C^3), 115.0 (C^{12}), 82.1 (C^{16}), 81.8 (C^{20}), 65.3 (C^{14}), 59.8 (C^9), 57.4 (C^{18}), 50.8 (C^{cyclen}), 27.1 (C^{21}), 27.0 (C^{17}). ESI-MS (*m/z)*: Found $[M + H]^+$ 1095.6167, calc $[C_{64}H_{82}N_6O_{10} + H]^+$ 1095.6165; Found $[M + Na]^+$ 1117.5987, calc $[C_{64}H_{82}N_6O_{10} + Na]^+$ 1117.5985; Found $[M + 2H]^{2+}$ 548.3119, calc $[C_{64}H_{82}N_6O_{10} + 2H]^{2+}$ 548.3119; Found $[M + H + Na]^{2+}$ 559.3027, calc $[C_{64}H_{82}N_6O_{10} + H + Na]^{2+}$ 559.3029.

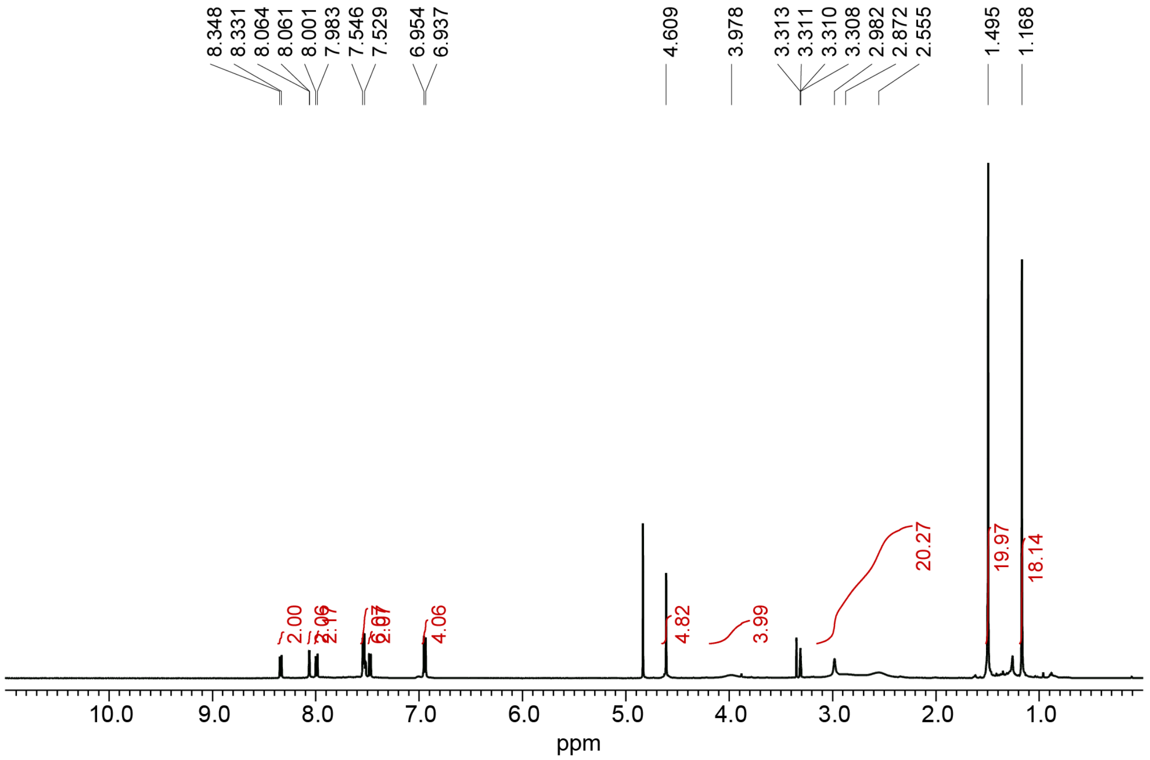

^{1}H NMR spectrum (500 MHz, CD_3OD, 298 K) of 4,10-*bis*((6-(4-(1,1-dimethylethyl)-phenoxyacetate)-quinolin-2-yl)-methyl)-1,4,7,10-tetraazacyclododecane-1,7-diyl)-diacetate (**5c**).

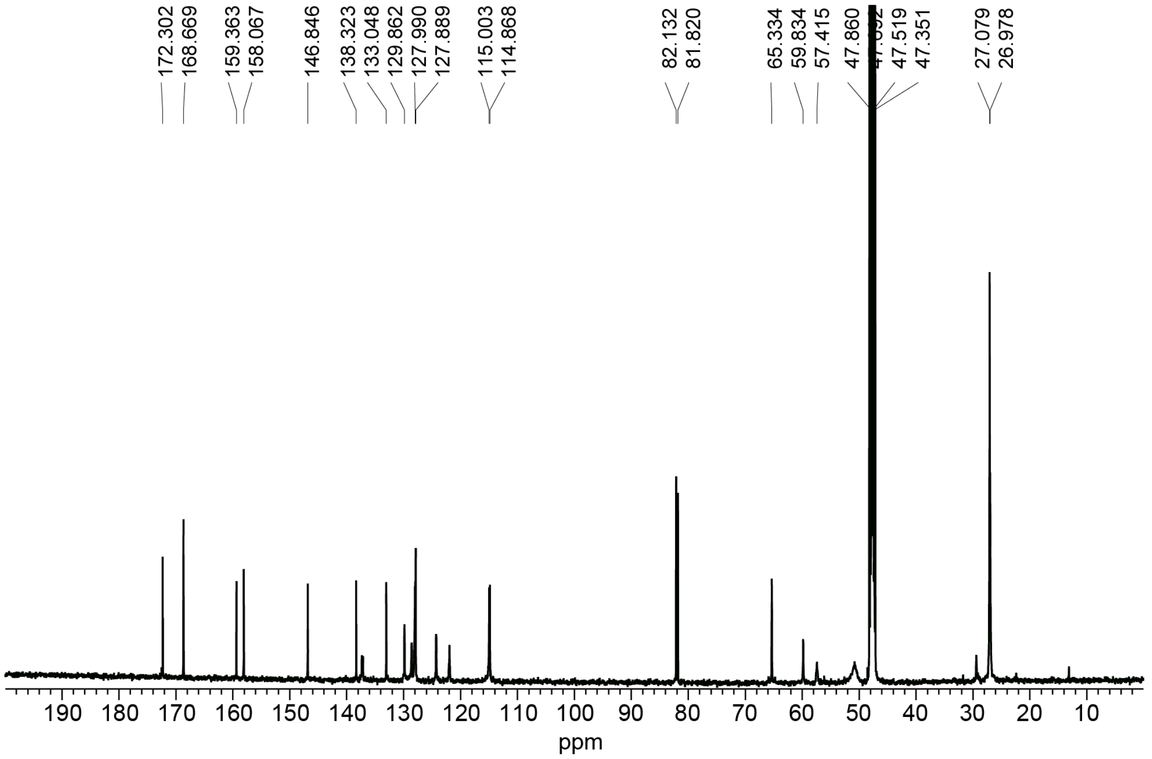

^{13}C NMR spectrum (126 MHz, CD_3OD, 298 K) of 4,10-*bis*((6-(4-(1,1-dimethylethyl)-phenoxyacetate)-quinolin-2-yl)-methyl)-1,4,7,10-tetraazacyclododecane-1,7-diyl)-diacetate (**5c**).

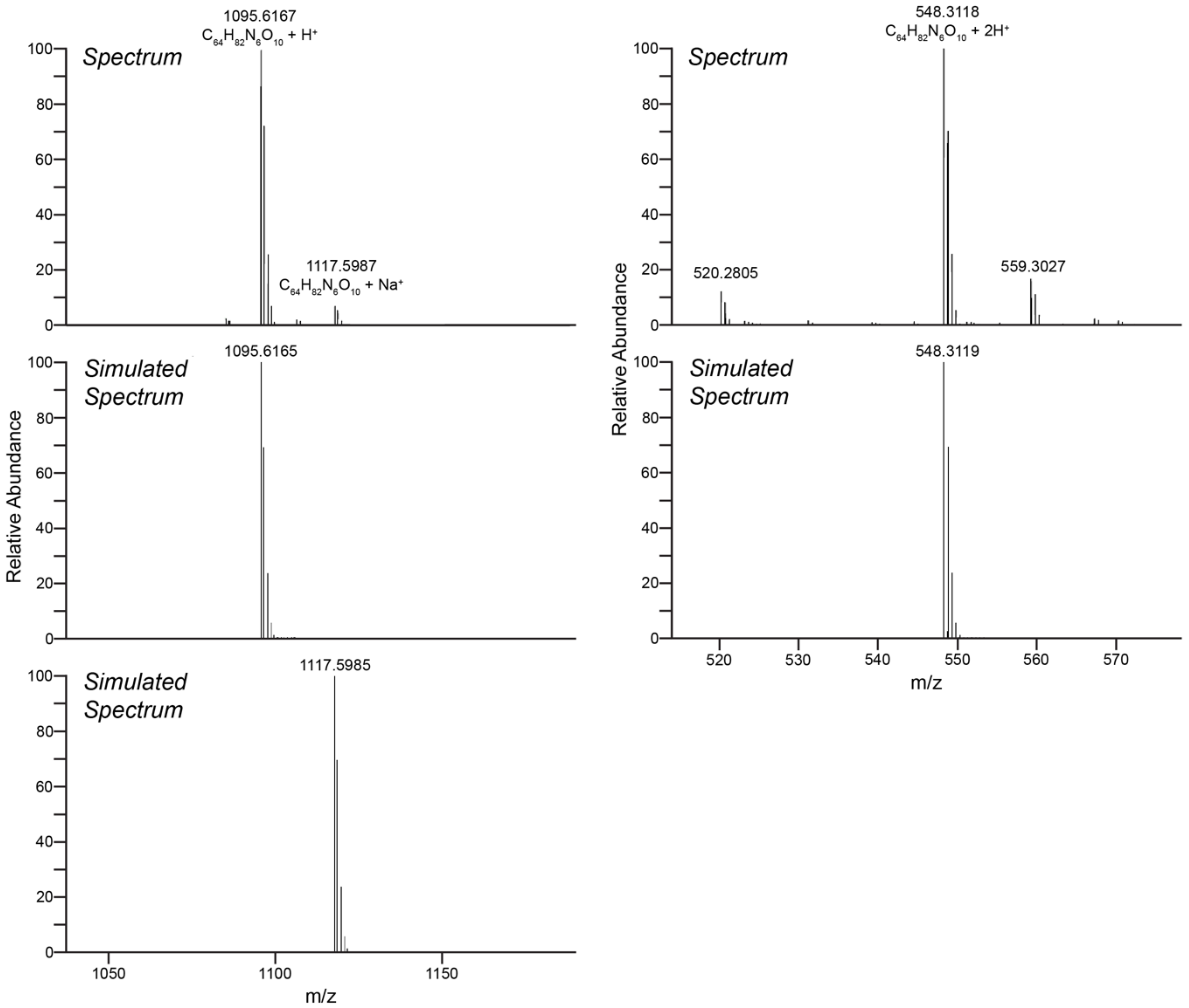

High-resolution mass spectra of 4,10-*bis*((6-(4-(1,1-dimethylethyl)-phenoxyacetate)-quinolin-2-yl)-methyl)-1,4,7,10-tetraazacyclododecane-1,7-diyl)-diacetate (**5c**).

4,10-*Bis*((6-(4-(1,1-dimethylethyl)-phenoxyacetate)-quinolin-2-yl)-methyl)-1,4,7,10-tetraazacyclododecane-1,7-diyl)-diacetic acid (**6c**)

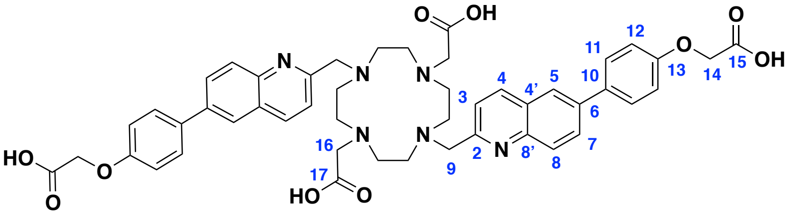

To a solution of 4,10-*bis*((6-(4-(1,1-dimethylethyl)-phenoxyacetate)-quinolin-2-yl)-methyl)-1,4,7,10-tetraazacyclododecane-1,7-diyl)-diacetate (61 mg, 56 μmol) in dichloromethane (2 mL) was added trifluoroacetic acid (2 mL). The reaction mixture was stirred at room temperature for 8 hours and the trifluoroacetic acid was co-evaporated with dichloromethane (5 x 20 mL) to give the deprotected molecule as an off-white solid (60 mg, 98%).

^{1}H NMR spectrum (400 MHz, CD_3OD): δ 8.34 (2H, d, *J* = 8.2 Hz, H^4), 8.20 (2H, d, *J* = 8.9 Hz, H^8), 8.04 (2H, d, *J* = 1.6 Hz, H^5), 7.97 (2H, d, *J* = 8.9 Hz, H^7), 7.75 (2H, d, *J* = 8.2 Hz, H^3), 7.67 (4H, d, *J* = 8.9 Hz, H^{11}), 7.05 (4H, d, *J* = 8.9 Hz, H^{12}), 4.42 (4H, s, H^{14}), 3.97 (4H, br s, H^9), 2.90 – 2.20 (20H, m, H^{cyclen}, H^{16}). ^{13}C NMR (101 MHz, CD_3OD): δ 177.8 (C^{17}), 175.1 (C^{15}), 160.1 (C^2), 158.8 (C^{13}), 146.6 ($C^{8'}$), 140.0 (C^6), 137.5 (C^4), 132.6 (C^{10}), 128.9 (C^7), 128.8 (C^8), 128.0 ($C^{4'}$), 127.9 (C^{11}), 124.1 (C^5), 122.2 (C^3), 115.0 (C^{12}), 67.2 (C^{14}), 61.0 (C^{16}), 60.9 (C^9), 51.2 (C^{cyclen}), 50.9 (C^{cyclen}), 50.4 (C^{cyclen}), 48.5 (C^{cyclen}). ESI-MS (*m/z)*: Found $[M + H]^+$ 871.3655, calc $[C_{48}H_{50}N_6O_{10} + H]^+$ 871.3661; Found $[M + 2H]^{2+}$ 436.1865, calc $[C_{48}H_{50}N_6O_{10} + 2H]^{2+}$ 436.1867.

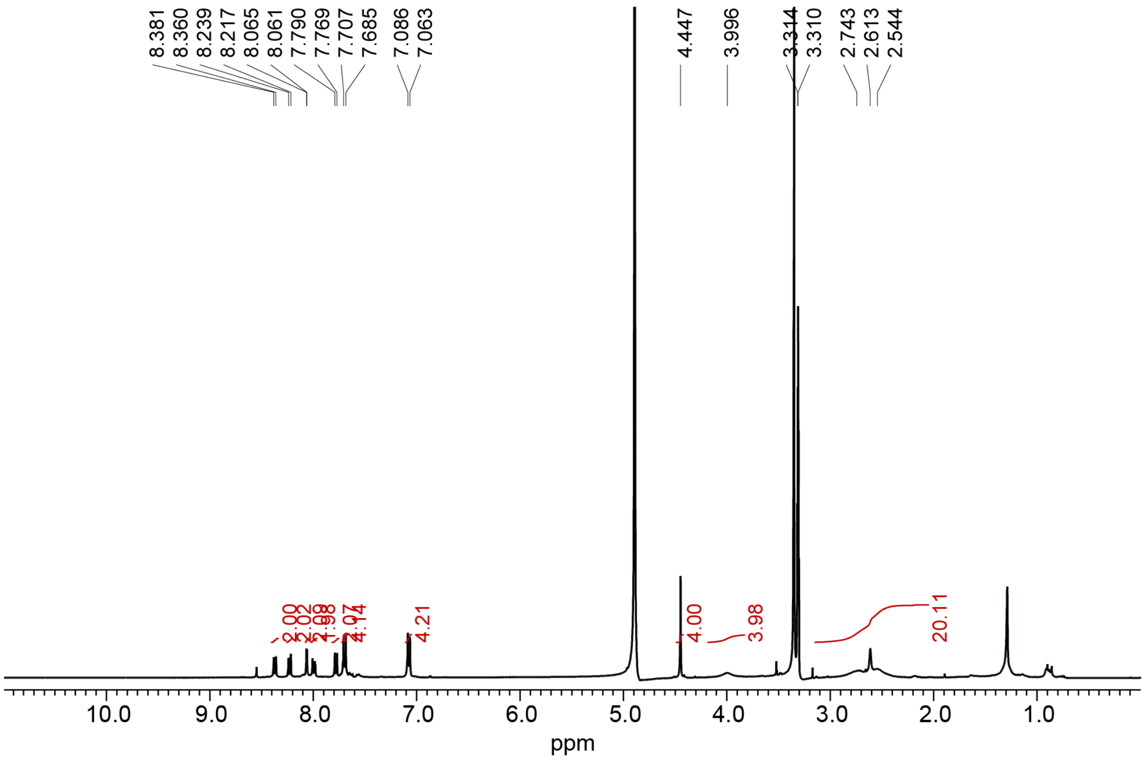

^{1}H NMR spectrum (400 MHz, CD_3OD, 298 K) 4,10-*bis*((6-(4-(1,1-dimethylethyl)-phenoxyacetate)-quinolin-2-yl)-methyl)-1,4,7,10-tetraazacyclododecane-1,7-diyl)-diacetic acid (**6c**).

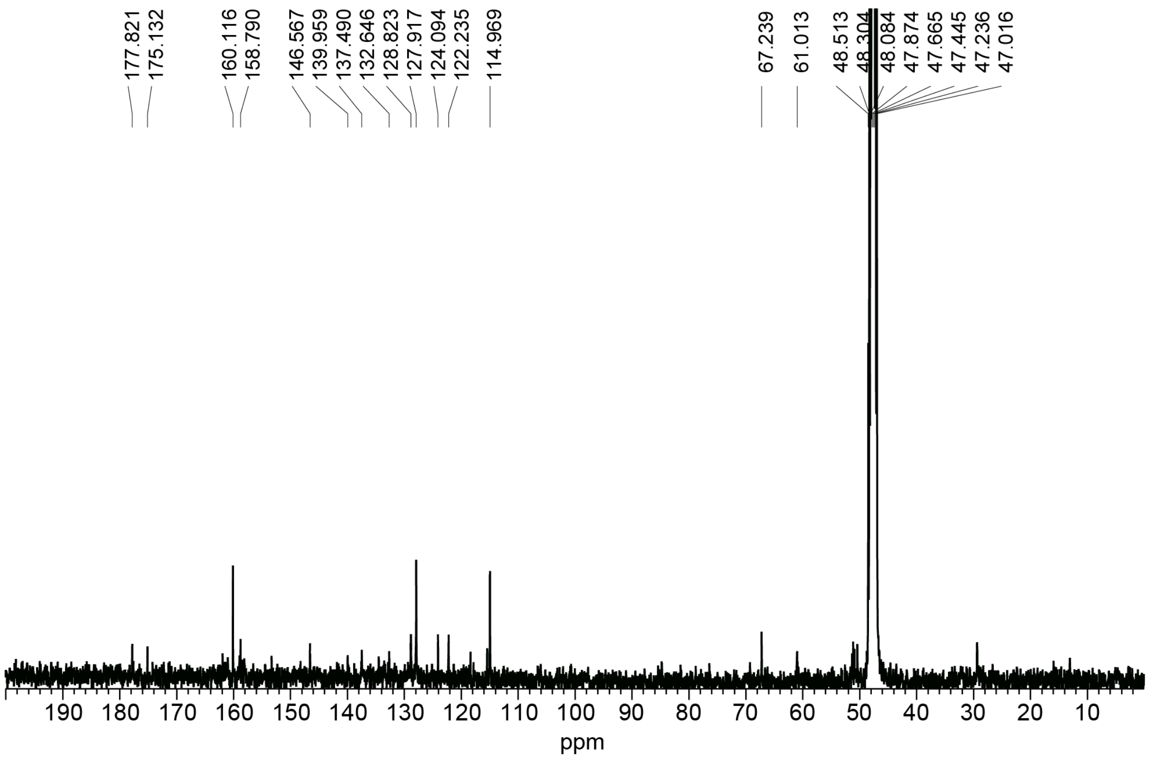

^{13}C NMR spectrum (101 MHz, CD_3OD, 298 K) of 4,10-*bis*((6-(4-(1,1-dimethylethyl)-phenoxyacetate)-quinolin-2-yl)-methyl)-1,4,7,10-tetraazacyclododecane-1,7-diyl)-diacetic acid (**6c**).

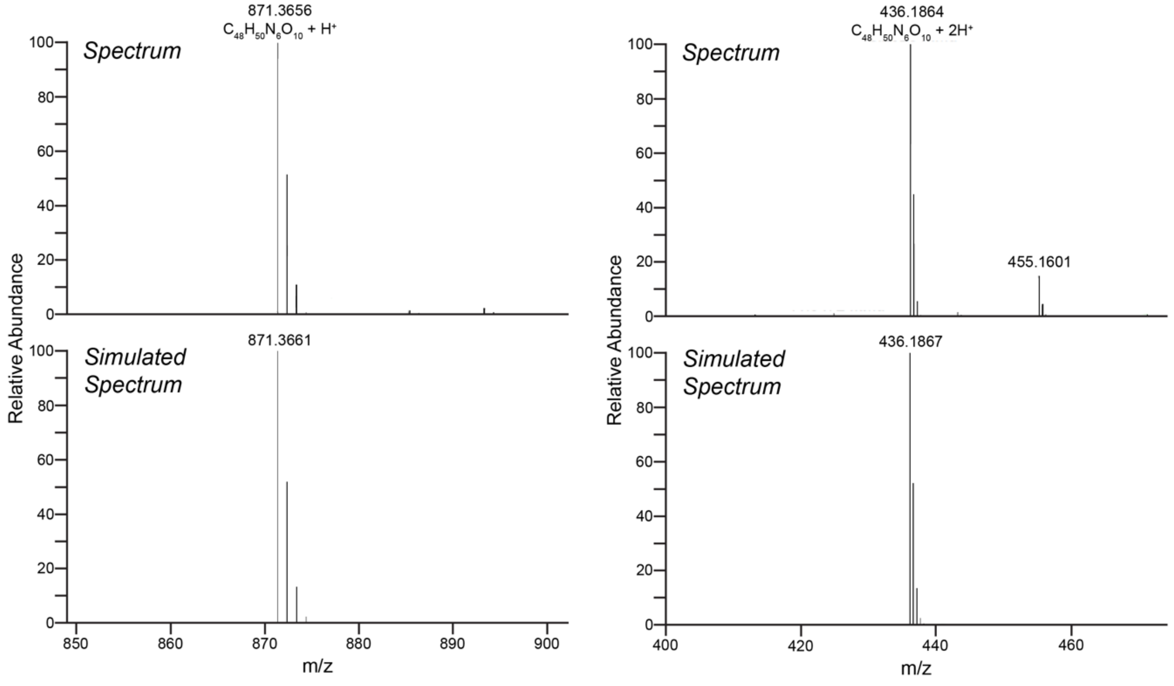

High-resolution mass spectra of 10-*bis*((6-(4-(1,1-dimethylethyl)-phenoxyacetate)-quinolin-2-yl)-methyl)-1,4,7,10-tetraazacyclododecane-1,7-diyl)-diacetic acid (**6c**).

[Eu.ADPGlow]⁻

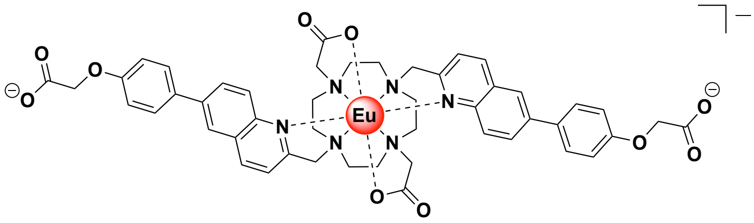

The deprotected ligand (56 mg, 0.06 mmol) was dissolved in methanol (2 mL) then potassium carbonate (2 mg, 0.02 mmol) and europium(III) trifluoromethanesulfonate (50 mg, 0.08 mmol) were added and the reaction heated to 50 °C for 48 hours. The reaction was cooled to room temperature, filtered and the solvent evaporated under reduced pressure. The crude material was purified by preparative RP-HPLC [gradient: 0 − 100% acetonitrile in 100 mM NH_4HCO_3 over 25 minutes, at 17 mL per minute] to give the complex as a pale-yellow solid (12 mg, 29%).

^{1}H NMR spectrum (500 MHz, CD_3OD): δ 47.4, 46.5, 45.3, 38.3, 37.3, 36.5, 35.8, 35.1, 31.7, 30.0, 24.2, 23.8, 23.2, 19.2, 18.2, 17.1, 16.9, 16.3, 15.5, 15.0, 14.2, 13.8, 13.6, 13.3, 13.1, 12.7, 12.5, 12.0, 11.8, 11.2, 11.0, 10.6, 10.1, 9.7, 9.4, 9.2, 8.5, 8.1, 7.9, 7.7, 7.2, 7.1, 6.8, 6.6, 6.0, 4.9, 4.8, 4.5, 3.3, 2.7, 2.2, 2.0, 1.3, 0.3, -0.1, -1.1, -2.0, -2.3, -3.0, -3.7, -4.5, -5.0, -6.7, -7.2, -7.8, -8.7, -9.8, -10.5, -11.8, -12.0, -13.2, -13.9, -14.3, -14.4, -14.7, -15.0, -15.3, -16.1, -16.3, -17.0, -17.6, -18.9, -19.3, -19.6, -19.8, -20.0, -20.4, -20.8, -22.7, -23.2, -23.6, -25.3, -25.8, -26.1, -28.1, -28.4. ESI-MS (*m/z*): Found $[M]^+$ 1021.2643, calc $[C_{48}H_{48}EuN_6O_{10}]^+$ 1021.2639. Photophysical data measured in 10 mM HEPES: λ_{max} = 337 nm, ε = 9200 M^{-1} cm^{-1}, Φ_{em} = 0.3%.

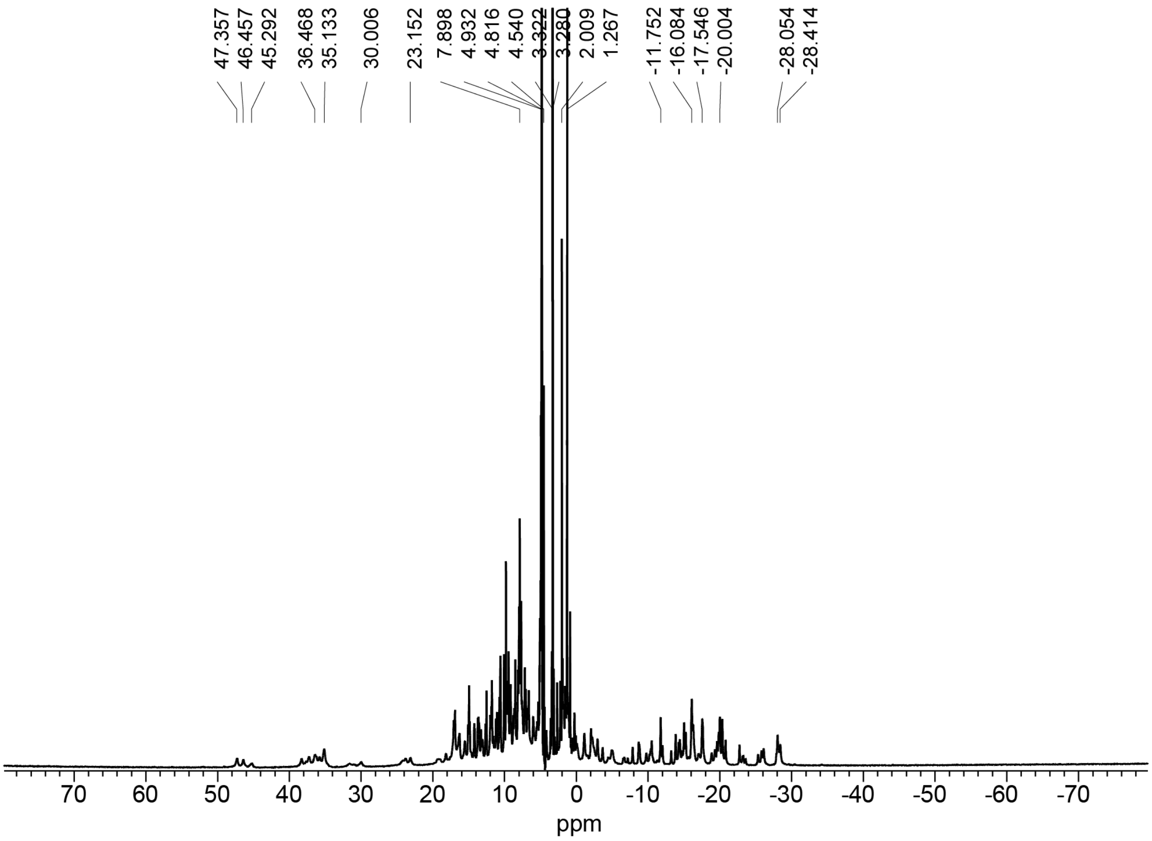

^{1}H NMR spectrum (500 MHz, CD_3OD, 298 K) of **[Eu.ADPGlow]⁻**.

[Gd.ADPGlow]⁻

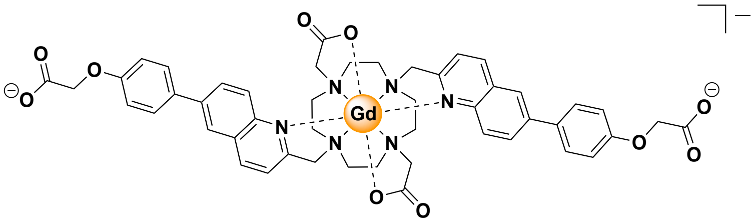

The deprotected ligand (20 mg, 0.02 mmol) was dissolved in methanol (2 mL). Potassium carbonate (12 mg, 0.09 mmol) and europium(III) trifluoromethanesulfonate (47 mg, 0.08 mmol) were added and the reaction heated to 50 °C for 6 days. The reaction was cooled to room temperature, filtered and the solvent evaporated under reduced pressure. The crude material was purified by semi-preparative RP-HPLC [gradient: 0 − 100% acetonitrile in 100 mM NH_4HCO_3 over 25 minutes, at 4 mL per minute] to give the complex as a pale-yellow solid (2.8 mg, 10%).

ESI-MS (*m/z)*: Found $[M]^+$ 1026.2672, calc $[C_{48}H_{48}GdN_6O_{10}]^+$ 1026.2668. Photophysical data measured in methanol: λ_{max} = 337 nm.

4. Photophysical measurements of Eu(III) complexes

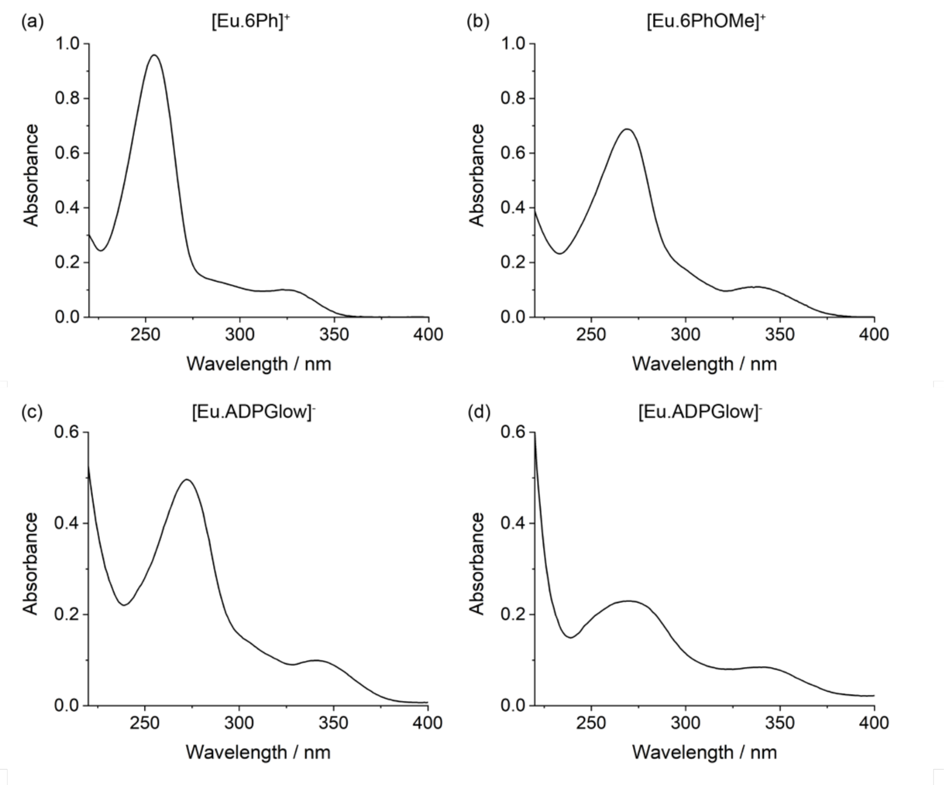

Figure S5. Absorption spectra of (a) [**Eu.6Ph**]$^+$, (b) [**Eu.6PhOMe**]$^+$, (c) [**Eu.ADPGlow**]$^-$ measured in methanol and (d) [**Eu.ADPGlow**]$^-$ measured in 10 mM HEPES at pH 7.0.

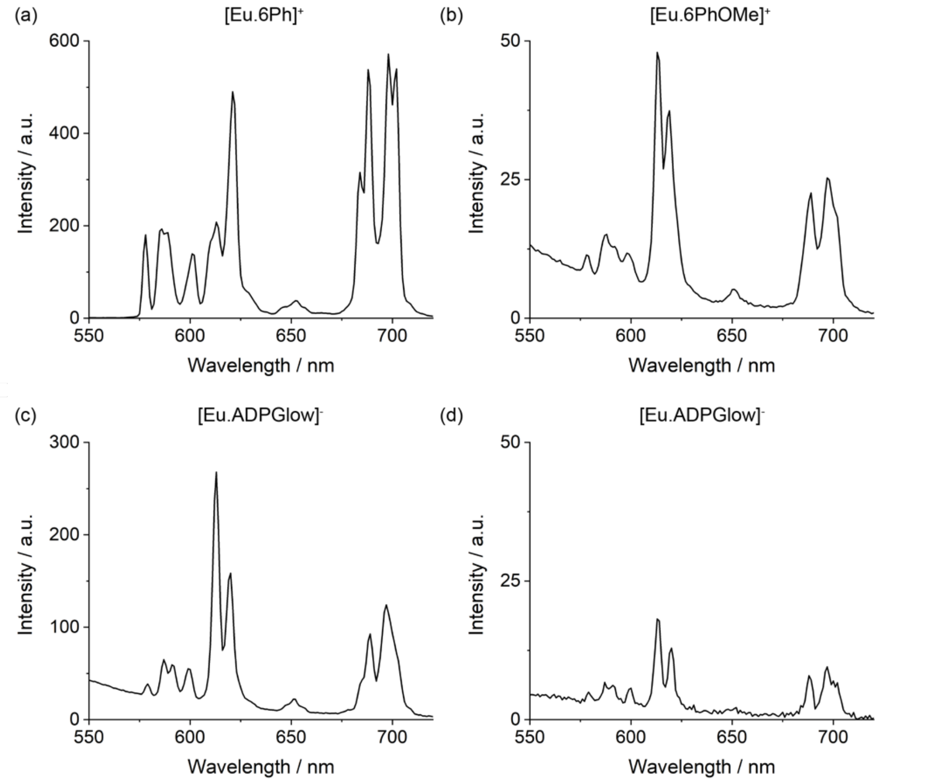

Figure S6. Emission spectra of (a) [**Eu.6Ph**]$^+$ (λ_{ex} = 328 nm), (b) [**Eu.6PhOMe**]$^+$ (λ_{ex} = 340 nm), (c) [**Eu.ADPGlow**]$^-$ (λ_{ex} = 337 nm) measured in methanol and (d) time-resolved emission (60–400 μs) of [**Eu.ADPGlow**]$^-$ (λ_{ex} = 337 nm) measured in 10 mM HEPES at pH 7.0:methanol (5%).

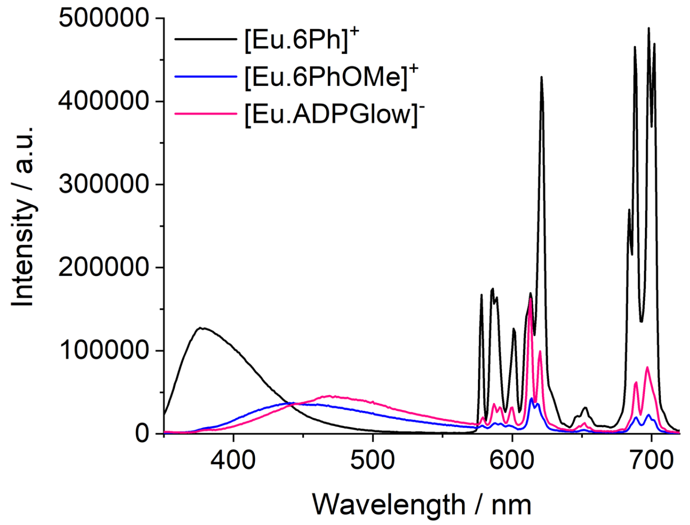

Figure S7. Emission spectra of **[Eu.6Ph]**$^+$ (λ_{ex} = 328 nm), (b) **[Eu.6PhOMe]**$^+$ (λ_{ex} = 340 nm), (c) **[Eu.ADPGlow]**$^-$ (λ_{ex} = 337 nm) displaying the ligand fluorescence and spectra used to calculate the quantum yields of each complex in methanol (NB: quantum yields were measured using quinine sulfate in 0.05 M H_2SO_4 as standard, Φ_{em} = 59%).

Table S1. Photophysical data for Gd(III) complexes measured in diethyl ether/isopentane/ethanol (*v/v* 5:5:2) at 293 K for absorbance and 77 K for emission.

		Adiabatic emission		Vertical emission	
	λ_{ex} / nm	λ_{em} / nm	E_T / cm^{-1}	λ_{em} / nm	E_T / cm^{-1}
**[Gd.6Ph]**$^+$	328	497	20121	533	18762
**[Gd.6PhOMe]**$^+$	342	511	19569	551	18149
**[Gd.ADPGlow]**$^-$	337	474	21097	508	19685

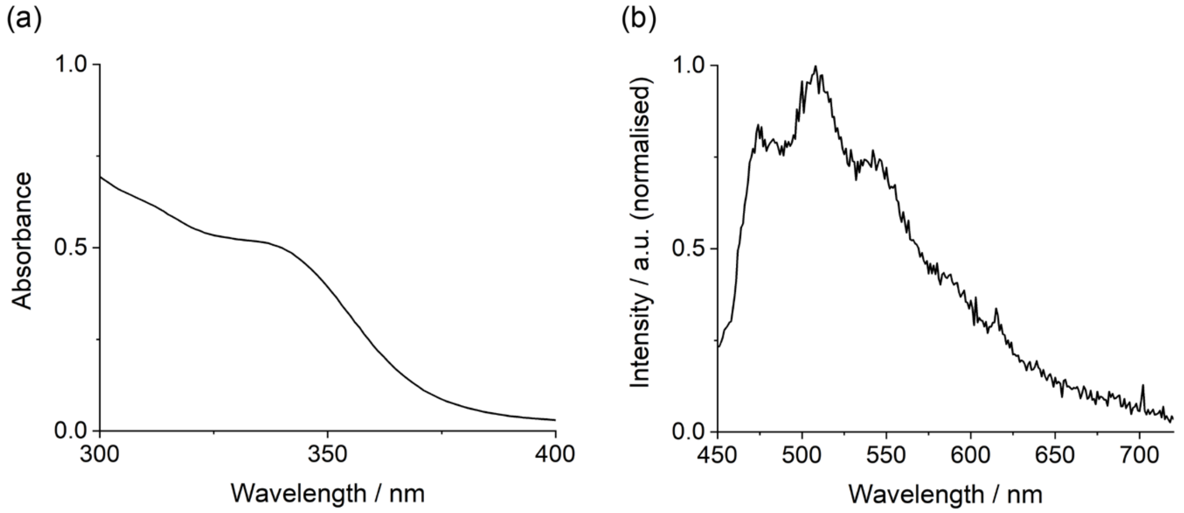

Figure S8. (a) Absorption and (b) emission spectra of **[Gd.ADPGlow]**$^-$. Measured in diethyl ether/isopentane/ethanol (*v/v* 5:5:2) at (a) 293 K; (b) 77 K, λ_{ex} = 337 nm.

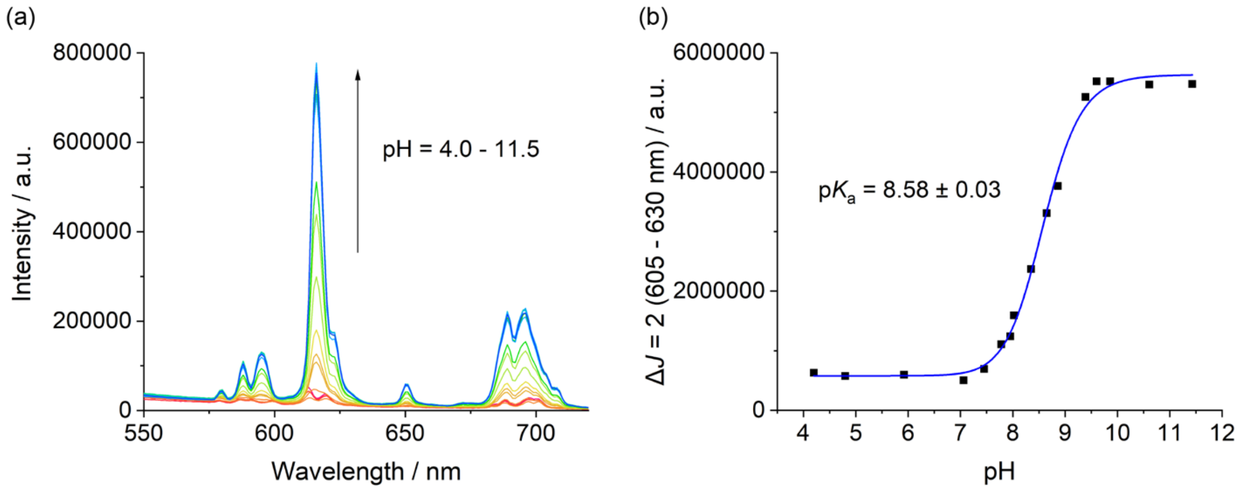

Figure S9. pH Titration of [**Eu.ADPGlow**]$^-$. (a) Increase in emission intensity upon addition of NaOH solution, where the pH was adjusted ~0.5 pH unit, (b) plot of emission intensity ΔJ = 2 (605 – 630 nm) as a function of pH, showing the fit to the observed data. Measured in water, 295 K, 0.1 Abs, λ_{ex} = 337 nm.

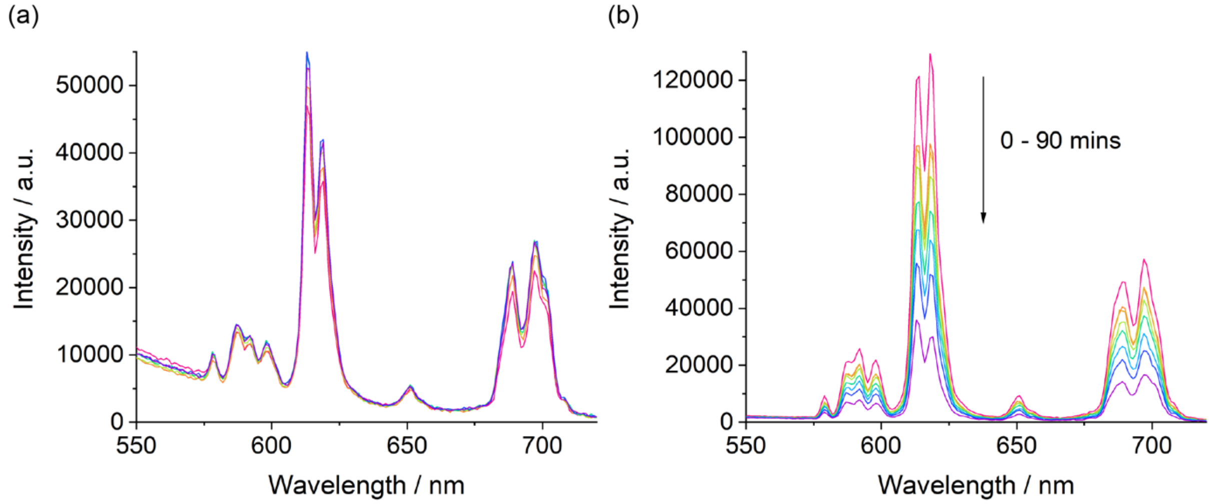

Figure S10. Stability of the emission response of [**Eu.6PhOMe**]$^+$ (0.1 Abs) over 1.5 hour incubation period measured in (a) methanol, (b) methanol:10 mM HEPES buffer at pH 7.0 (1:9). λ_{ex} = 340 nm, 295 K.

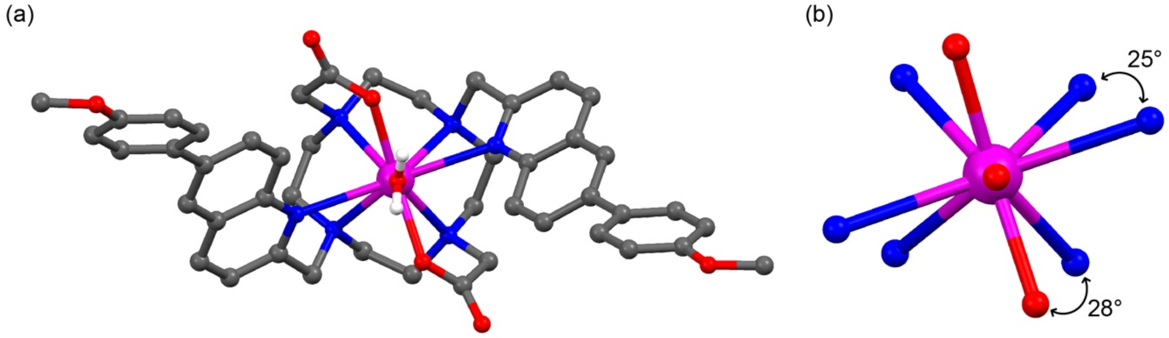

Figure S11. (a) Single crystal X-ray structure of [**Gd.6PhOMe**]$^+$ viewed along the main pseudo-C_2 axis. The hydrogen atoms of the coordinating water molecule are shown, all other hydrogen atoms, lowest occupancy macrocycle disorder, non-coordinating water molecules and the triflate counter ion have been omitted for clarity. (b) The coordination of the Gd(**III**) metal ion, displaying the twisted square antiprismatic geometry along the Gd-OH_2 axis. The hydrogen atoms have been omitted for clarity. Atom colours: Gd pink, C grey, N blue, O red, H white.

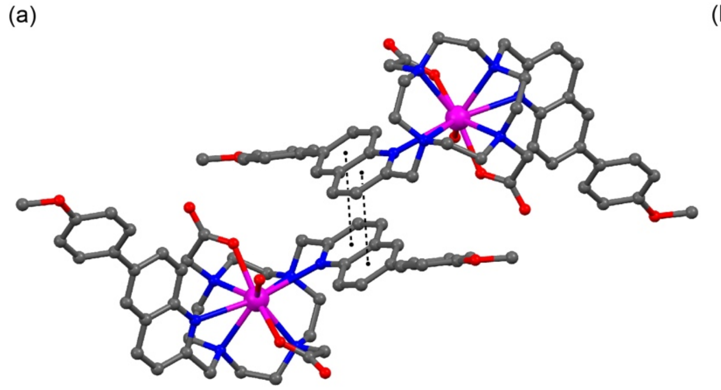

Figure S12. Perspective view of the X-ray crystal structure of [**Gd.6PhOMe**]$^{+}$ displaying the intermolecular π-π stacking between (a) quinoline rings and (b) the phenyl ring and quinoline ring within the unit cell. The hydrogen atoms, lowest occupancy macrocycle disorder, non-coordinating water molecules and counter ions have been omitted for clarity. Atom colours: Gd pink, C grey, N blue, O red.

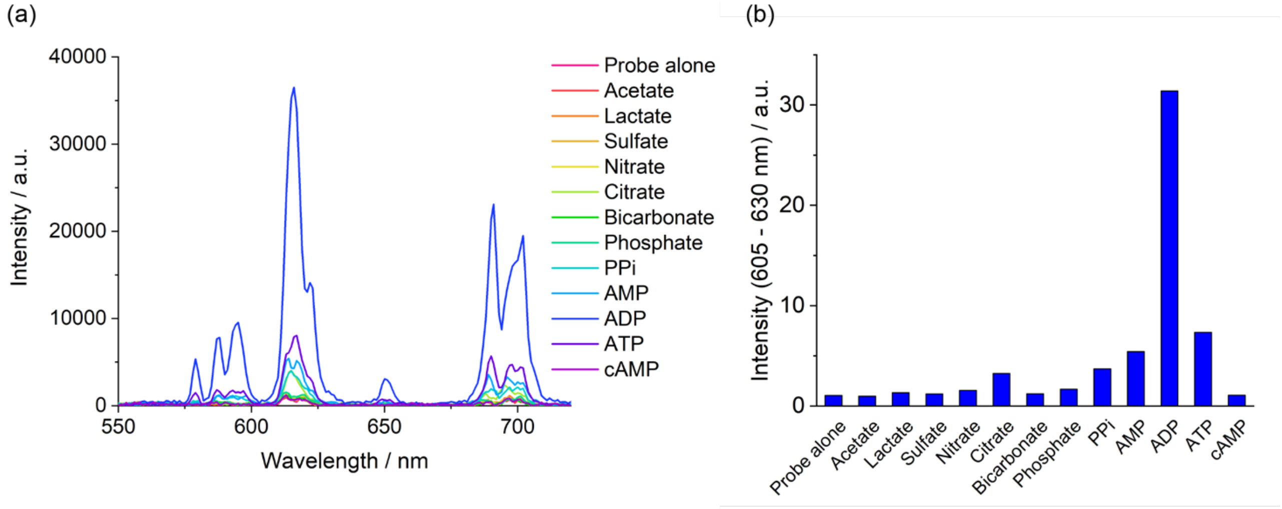

Figure S13. Selective emission enhancement of (a) [**Eu.ADPGlow**]$^{-}$ (0.1 Abs, λ_{ex} = 337 nm), with acetate, lactate, sulfate, nitrate, citrate, bicarbonate, phosphate, pyrophosphate (PPi), adenosine monophosphate (AMP), adenosine diphosphate (ADP), adenosine triphosphate (ATP), cyclic adenosine monophosphate (cAMP) (1 mM each). (b) Bar graph displaying emission enhancement of the ΔJ = 2 (605 – 630 nm) of [**Eu.ADPGlow**]$^{-}$ with selected anions (1 mM each), reproduced from Figure 4 of main manuscript.

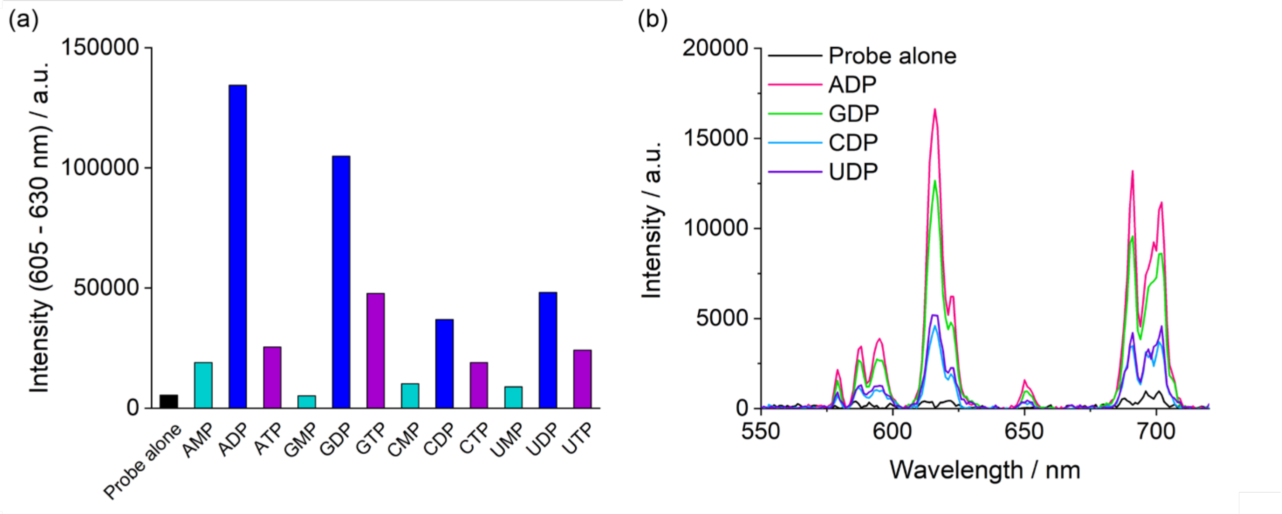

Figure S14. (a) Bar graph displaying the emission enhancement of the ΔJ = 2 (605 – 630 nm) of [**Eu.ADPGlow**]$^-$ (0.1 Abs, λ_{ex} = 337 nm), with mono-, di- and tri- nucleoside polyphosphate anions of adenine, guanine, cytosine and uracil (1 mM) each. (b) Emission spectra enhancement of [**Eu.ADPGlow**]$^-$ (0.1 Abs, λ_{ex} = 337 nm), with nucleoside diphosphate anions.

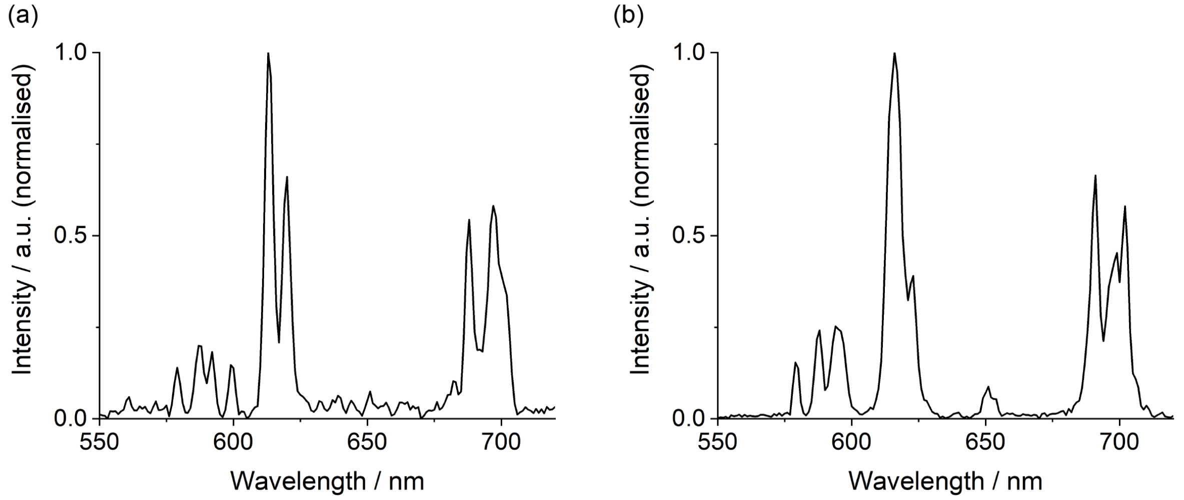

Figure S15. Normalised time-resolved emission spectra of (a) [**Eu.ADPGlow**]$^-$ (1.0 Abs) and (b) [**Eu.ADPGlow**]$^-$ (0.1 Abs) + 1 mM ADP showing the spectral form change. Measured in 10 mM HEPES at pH 7.0 at 295 K, λ_{ex} = 337 nm.

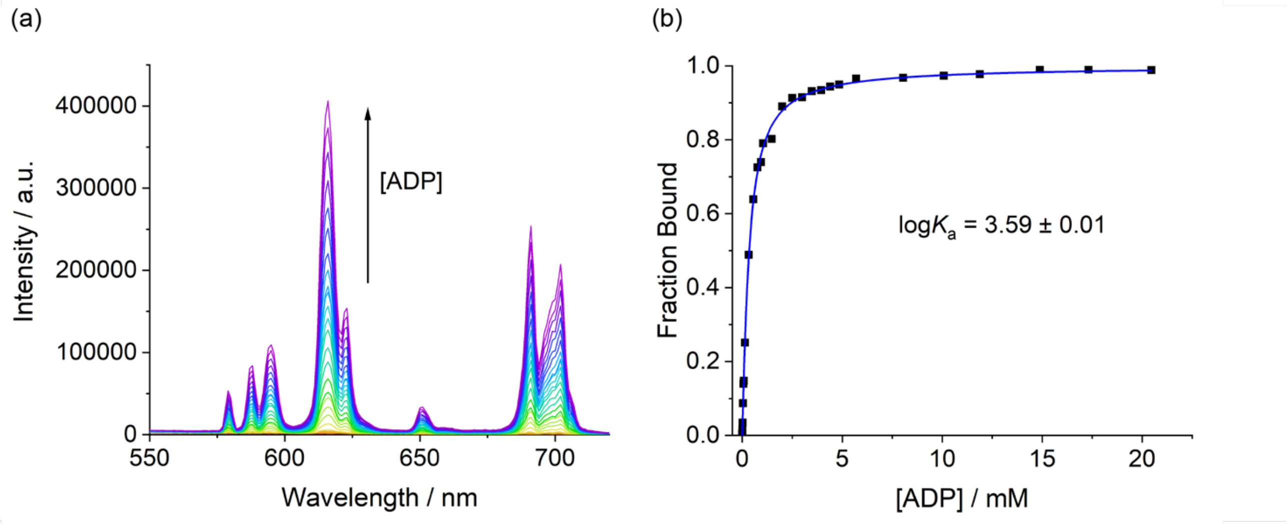

Figure S16. (a) Variation in emission spectra of [**Eu.ADPGlow**]$^-$ upon incremental addition of ADP. (b) Plot of fraction bound (determined from $\Delta J = 2$ / $\Delta J = 1$ intensity ratio) versus ADP concentration, showing the fit to a 1:1 binding isotherm. Measured in 10 mM HEPES at pH 7.0 at 295 K, λ_{ex} = 337 nm.

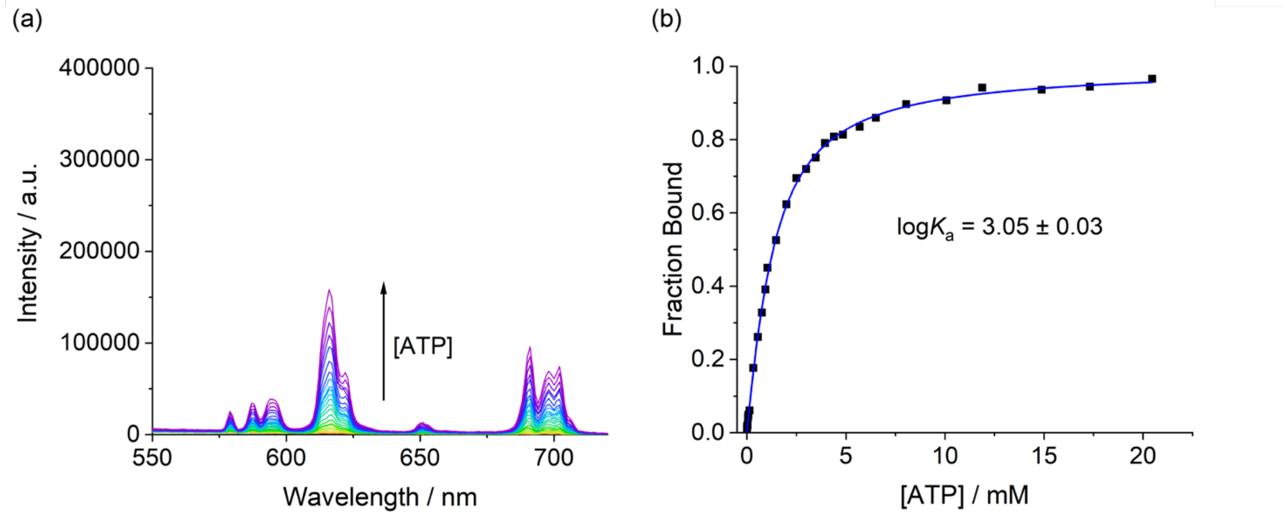

Figure S17. (a) Variation in emission spectra of [**Eu.ADPGlow**]$^-$ upon incremental addition of ATP. (b) Plot of fraction bound (determined from $\Delta J = 2$ / $\Delta J = 1$ intensity ratio) versus ATP concentration, showing the fit to a 1:1 binding isotherm. Measured in 10 mM HEPES at pH 7.0 at 295 K, λ_{ex} = 337 nm.

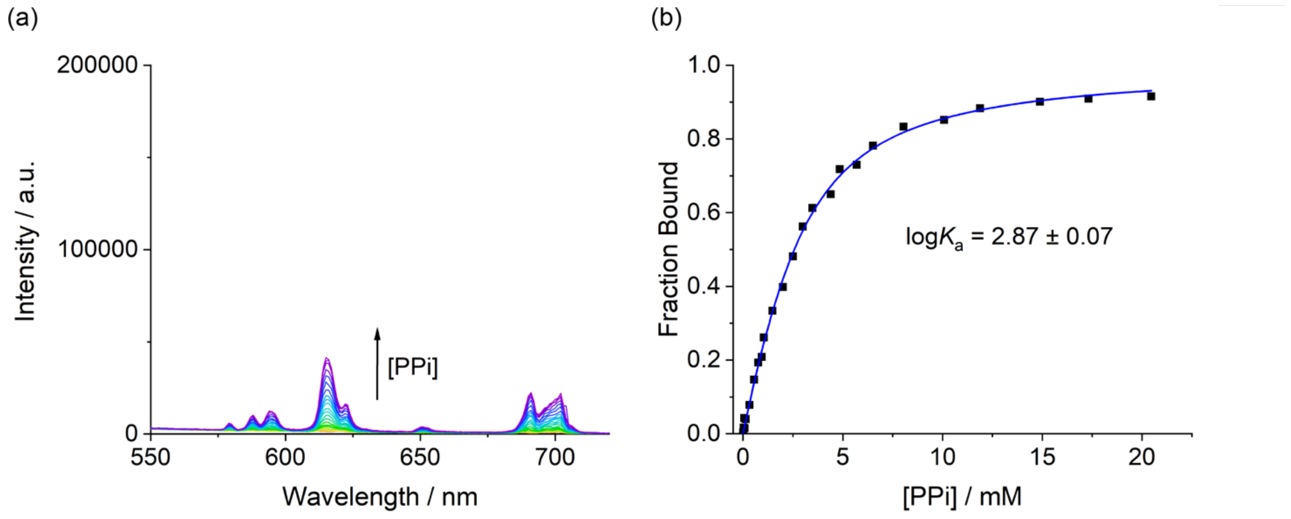

Figure S18. (a) Variation in emission spectra of [**Eu.ADPGlow**]$^-$ upon incremental addition of pyrophosphate. (b) Plot of fraction bound (determined from $\Delta J = 2$ / $\Delta J = 1$ intensity ratio) versus pyrophosphate concentration, showing the fit to a 1:1 binding isotherm. Measured in 10 mM HEPES at pH 7.0 at 295 K, λ_{ex} = 337 nm.

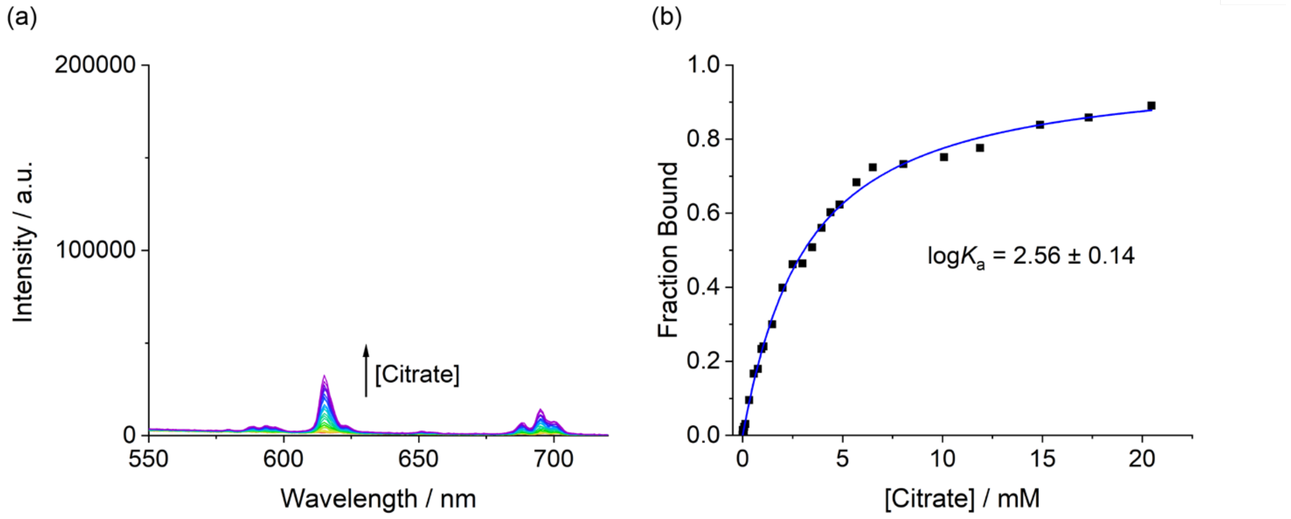

Figure S19. (a) Variation in emission spectra of [**Eu.ADPGlow**]$^-$ upon incremental addition of citrate. (b) Plot of fraction bound (determined from $\Delta J = 2$ / $\Delta J = 1$ intensity ratio) versus citrate concentration, showing the fit to a 1:1 binding isotherm. Measured in 10 mM HEPES at pH 7.0 at 295 K, λ_{ex} = 337 nm.

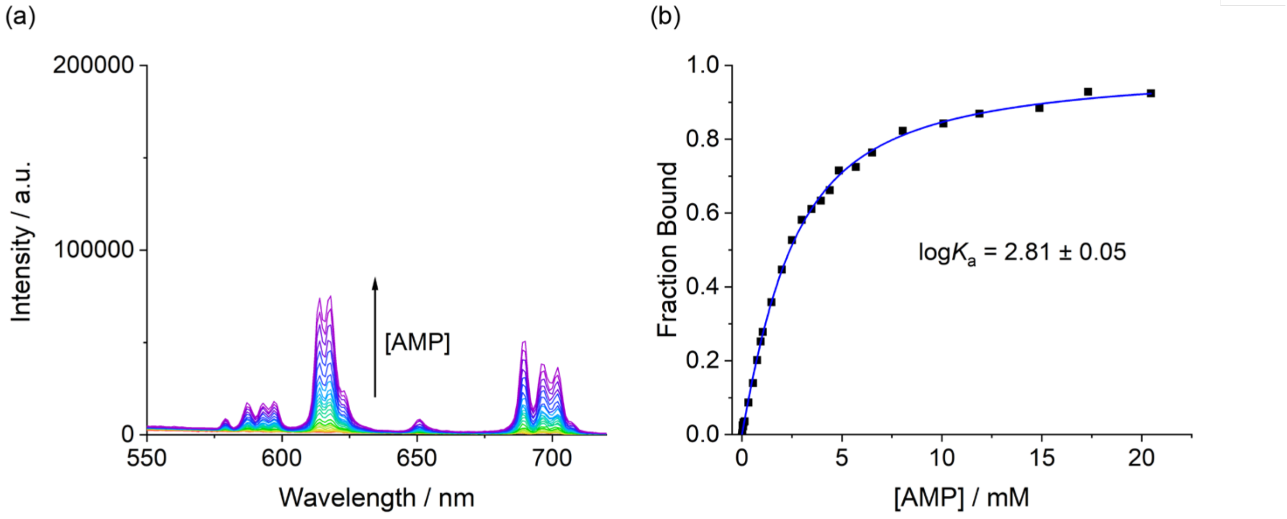

Figure S20. (a) Variation in emission spectra of [**Eu.ADPGlow**]$^-$ upon incremental addition of AMP. (d) Plot of fraction bound (determined from $\Delta J = 2$ / $\Delta J = 1$ intensity ratio) versus AMP concentration, showing the fit to a 1:1 binding isotherm. Measured in 10 mM HEPES at pH 7.0 at 295 K, λ_{ex} = 337 nm.

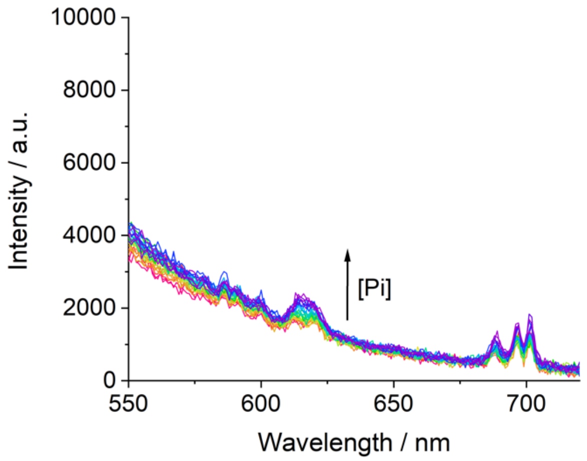

Figure S21. Variation in emission spectra of [**Eu.ADPGlow**]$^-$ upon incremental addition of inorganic phosphate (P_i). Measured in 10 mM HEPES at pH 7.0 at 295 K, λ_{ex} = 337 nm.

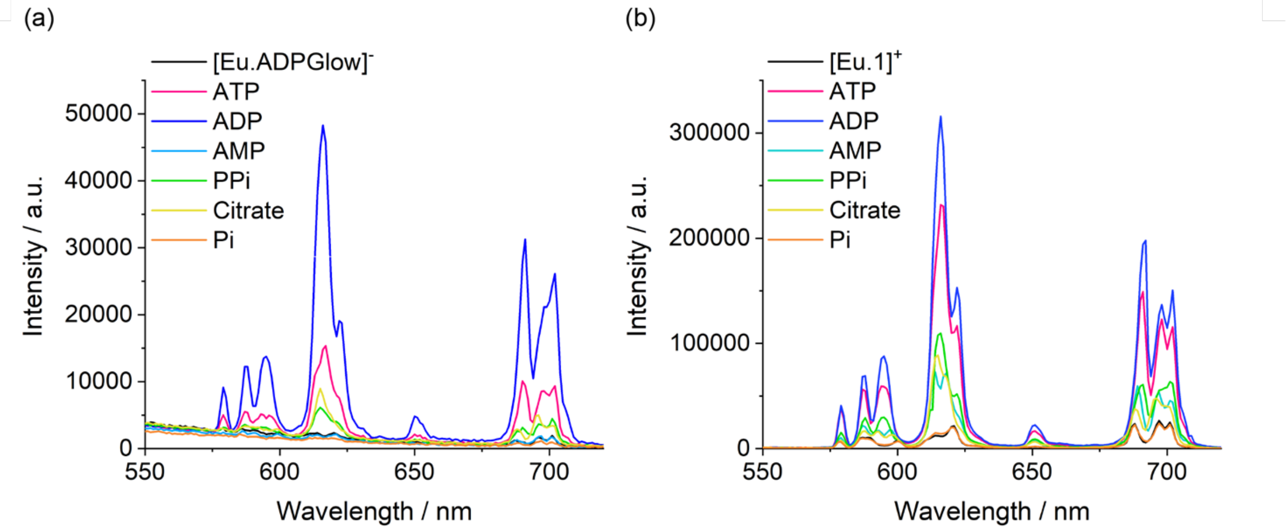

Figure S22. Emission enhancement of (a) [**Eu.ADPGlow**]$^-$ (0.1 Abs, λ_{ex} = 337 nm) and (b) [**Eu.1**]$^+$ (0.1 Abs, λ_{ex} 330 nm) with selected anions adenosine triphosphate (ATP), adenosine diphosphate (ADP), adenosine monophosphate (AMP), pyrophosphate (PPi), citrate and phosphate (Pi) (1 mM each). Measured in 10 mM HEPES at pH 7.0 at 295 K.

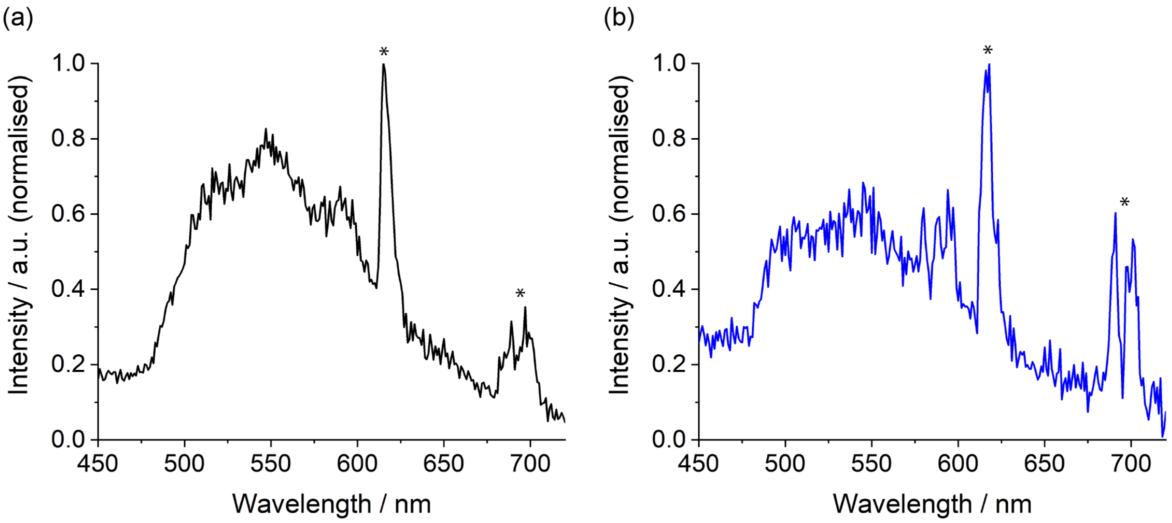

Figure S23. Phosphorescence spectra of (a) [**Gd.ADPGlow**]$^-$ alone; (b) [**Gd.ADPGlow**]$^-$ in the presence of 1 mM ADP. Measured in diethyl ether/isopentane/ethanol/water (*v/v* 5:5:3:1) at 77 K, λ_{ex} = 337 nm. *Eu(III) complex impurity within the sample.

The phosphorescence spectra of the Gd(III) complex [**Gd.ADPGlow**]$^-$ shown in Figure S22 was recorded at 77 K in the absence and presence of 1 mM ADP. To address the lack of solubility of ADP in EPA solvent, water was added, resulting in a final solvent mixture of diethyl ether/isopentane/ethanol/water (v/v 5:5:3:1). However, this solvent composition produced a less transparent glass upon cooling to 77 K, compromising the S/N and overall quality of the phosphorescence spectra. Despite this, the spectra show that the position of the highest energy band remains essentially unchanged in the presence of ADP, at 508 nm (19,685 cm^{-1}), which is appropriately positioned for efficient sensitization of the europium(III) $^5D_0$ excited states at 17,300 cm^{-1}.

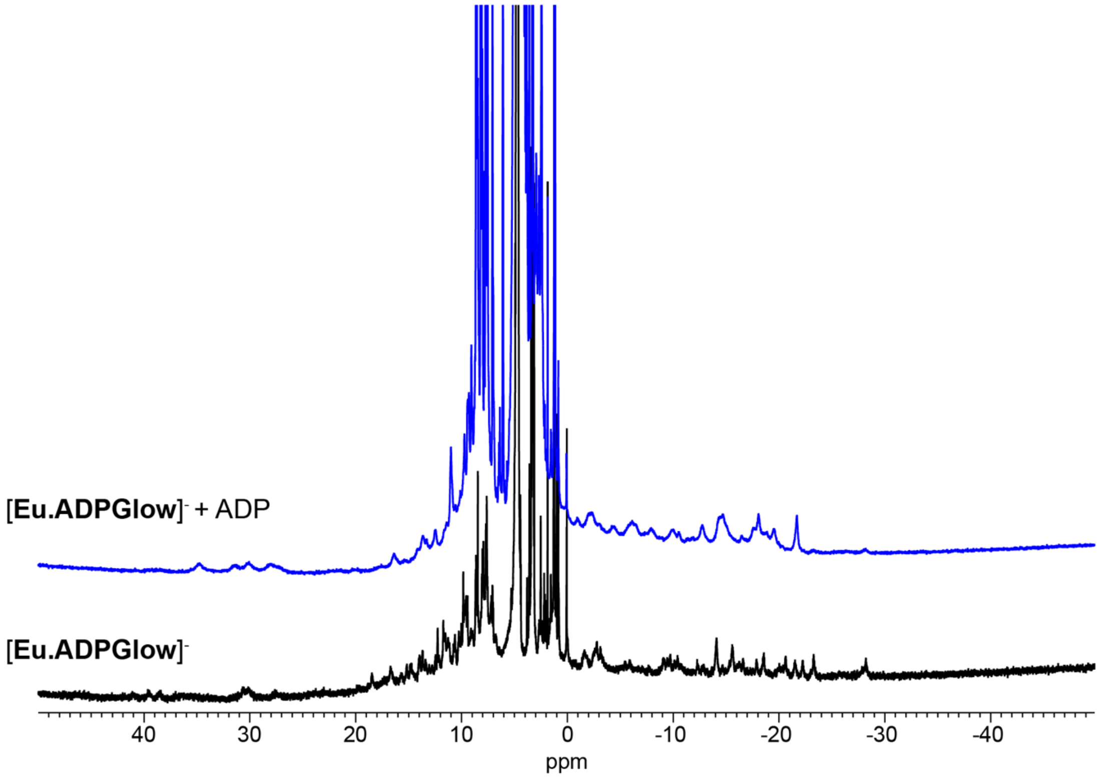

Figure S24. ^{1}H NMR spectra (500 MHz, 1:1 CD_3OD:D_2O) of [**Eu.ADPGlow**]$^-$ in the presence of ADP (2 equiv.) (blue) and [**Eu.ADPGlow**]$^-$ (2.2 mM) (black), recorded at 298 K at pD 7.0.

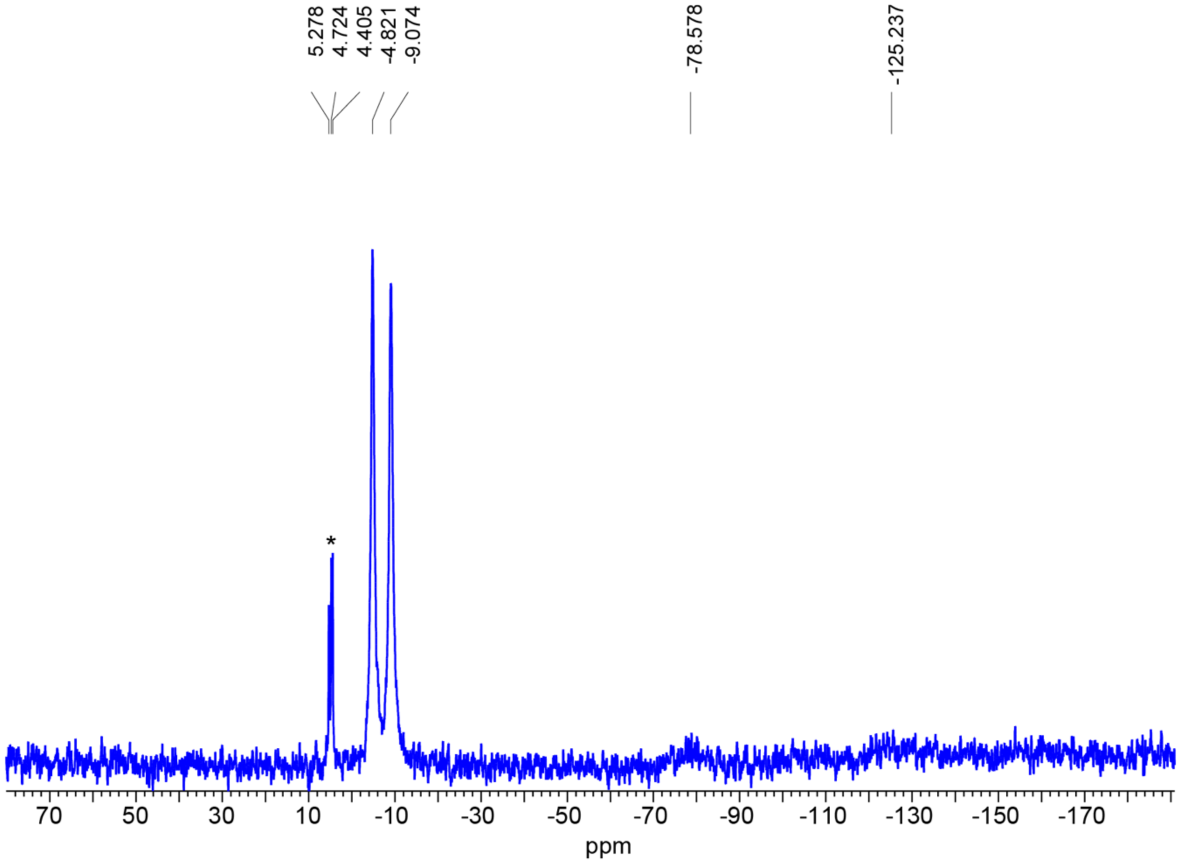

Figure S25. ^{31}P NMR spectra (202 MHz, 1:1 $CD_3OD:D_2O$) of [**Eu.ADPGlow**]$^-$ (2.2 mM) in the presence of ADP (2 equiv.) recorded at 298 K at pD 7.0. *Hydrolysed ADP into inorganic phosphate and AMP.

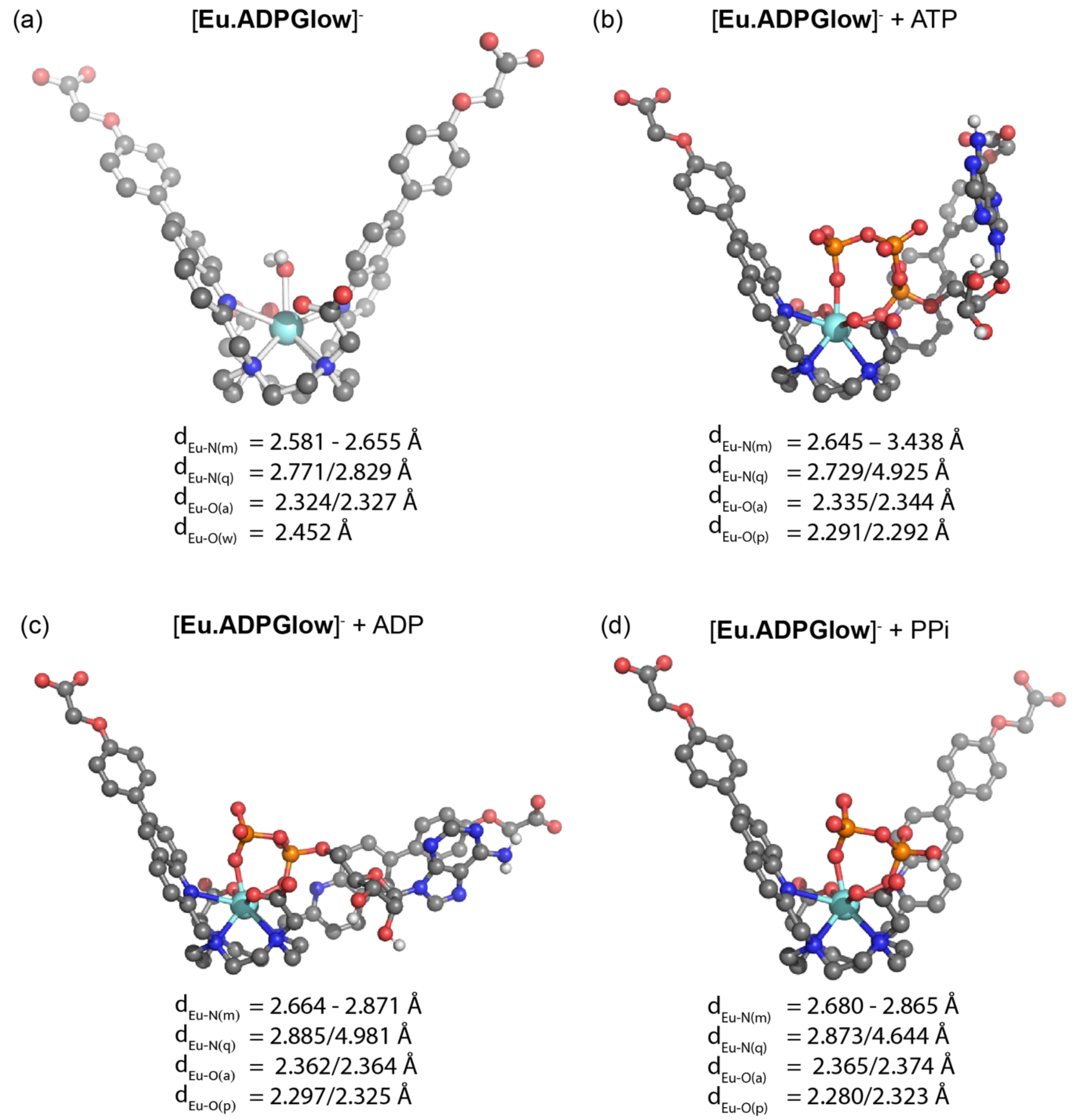

Figure S26. DFT-optimised molecular structure of [**Eu.ADPGlow**]$^-$ using the yttrium(**III**) ion, bound to (a) a water molecule, (b) ATP, (c) ADP, and (d) pyrophosphate. Bond distances of the central metal to ligand atoms are represented as: N(m) – macrocycle, N(q) – quinoline, O(a) – acetate, O(w) – water, O(p) – phosphate.

Table S2. Computed binding energies (kJ/mol) between [**Eu.ADPGlow**]$^-$ and ADP, ATP and pyrophosphate.

Anion	Δ°G / kJ/mol (Exp)	Δ°G / kJ/mol (Theo)
ADP	-30.4	-41.2
ATP	-27.4	-31.6
PPi	-26.6	-28.9

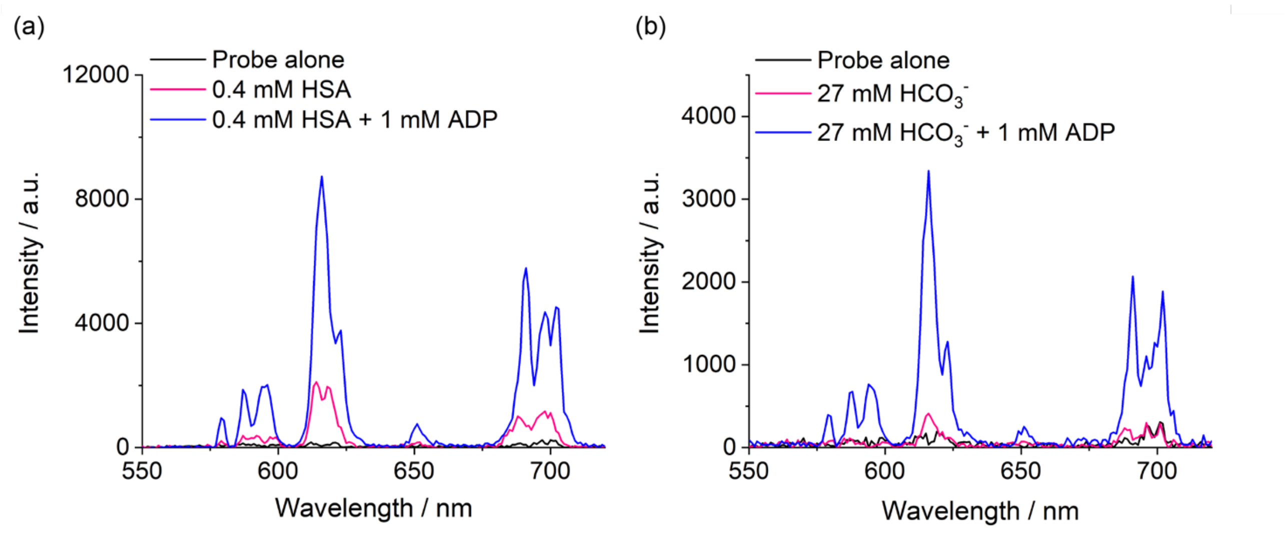

Figure S27. Time-resolved emission spectra of [**Eu.ADPGlow**]$^-$ (0.1 Abs, λ_{ex} = 337 nm) showing small increases in emission with biologically relevant concentrations of (a) human serum albumin (HSA, 0.4 mM) and (b) bicarbonate (27 mM), compared with the much larger emission enhancement upon subsequent addition of ADP (1 mM). Measured in 10 mM HEPES at pH 7 at 295 K.

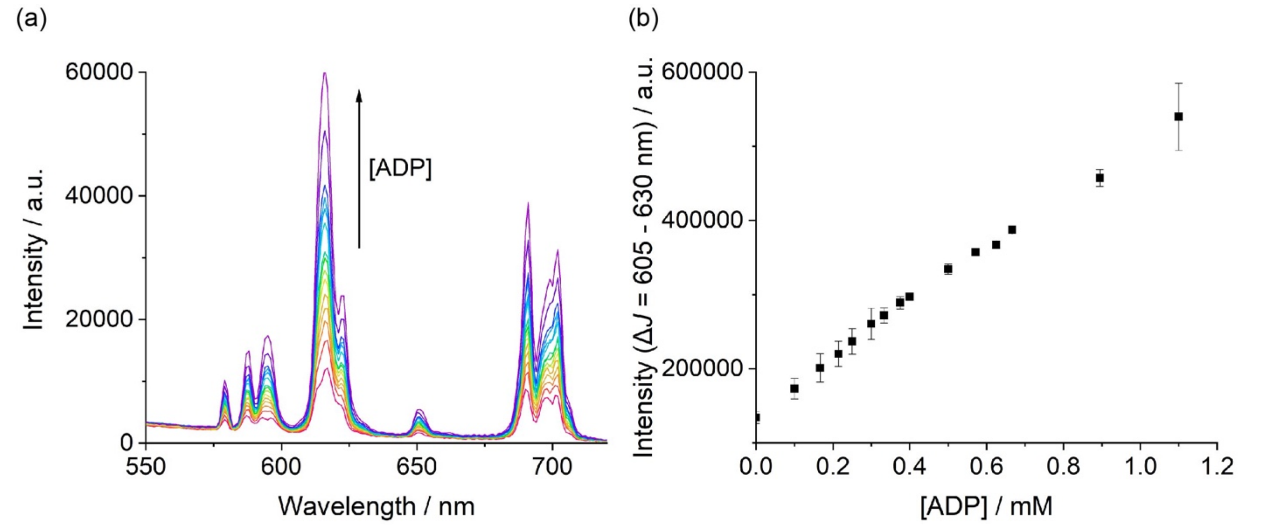

Figure S28. (a) Competition experiment showing the increase in emission spectra of [**Eu.ADPGlow**]$^-$ (10 μM) upon addition of ADP (0 – 1.2 mM) in background of ATP (1 mM). (b) Plot of the emission intensity of the Δ*J* = 2 region (605 – 630 nm) showing the 5-fold increase in emission upon addition of ADP. Measured in 10 mM HEPES at pH 7.0, containing 1 mM ATP, at 295 K, λ_{ex} = 337 nm.

5. X-ray Crystallography of [Gd.6PhOMe]$^+$

Colourless plate-like crystals of [**Gd.6PhOMe**]$^+$, suitable for single crystal X-ray diffraction were grown by slow evaporation of the complex dissolved in methanol/water (1:1).

Single crystal X-ray diffraction experiments were performed by the UK National Crystallography Service on a Rigaku FRE+ diffractometer with HF Varimax confocal mirrors, an UG2 goniometer and HyPix 6000HE detector. The crystals were collected at 100(2) K. The structure was solved by direct methods using ShelXT[3] and refined with ShelXL[4] using a least squares method. Olex2 software[5] was used as the solution, refinement and analysis program.

All non-hydrogen atoms were refined anisotropically and all hydrogen atoms were geometrically placed and refined using a riding model.

Disorder and Refinement Special Details
The macrocycle has been modelled over two sites. The parts have been fixed with occupancies of 0.7 and 0.3 for parts 1 and 2, respectively. To aid refinement of the model the anisotropic displacement parameters of carbon atoms (C39A, C40A, C41A, C42A, C43A, C44A, C45A, C46A) which form the low occupancy part 2 of the cyclen ring were constrained to be identical (EADP), due to their low occupancy, overlapping with the other atoms in close proximity to the Gd atom. The triflate anion has been modelled over two sites, with the largest occupancy as 0.61. The triflate anion has been added as a fragment and refined with restraining the Uij components due to the disorder. The non-coordinating water molecules have been modelled; however, a solvent mask was used to confirm the presence of 10 H_2O, (volume of 1376 cubic angstroms in 1 void per unit cell, which is consistent with the presence of 10 water molecules per asymmetric unit which accounts for 400 electrons per unit cell). Nine of these water solvates are disordered over two sites. Due to the poor resolution/data quality of these water molecules, the water hydrogen atoms were not observed in the electron density map and not included in the model. The anisotropic displacement parameter of the water residues is restrained to have more isotropic character (ISOR). Large residual electron density peaks (3.07 and 3.57 e A^{-3}) are located on opposite sides of the gadolinium atom. The size of these has been reduced by truncating the data used in the refinement to a resolution of 0.84 Å (SHEL 999 0.84). They are likely to be the result of deficiencies in the absorption correction, with the large atomic mass of Gd.

Crystal Packing
The methoxy-phenyl rings display twists co-planar to the quinoline rings (33.3(2)° and 33.6(2)°); with one ring involved in weak intermolecular π-π stacking interactions with the quinoline ring of a neighbouring complex. Additional intermolecular π-π interactions are observed between the quinoline rings, with the complexes stacking head-to-tail, with water molecules situated around the structures in the crystal packing. Indeed, there are several water molecules within the binding cavity which we expect to be involved in a hydrogen bonding network. However, the hydrogen atoms were not observed in the electron density map and to aid in the refinement these have been omitted in the model.

Deposited CIF number: 2381436

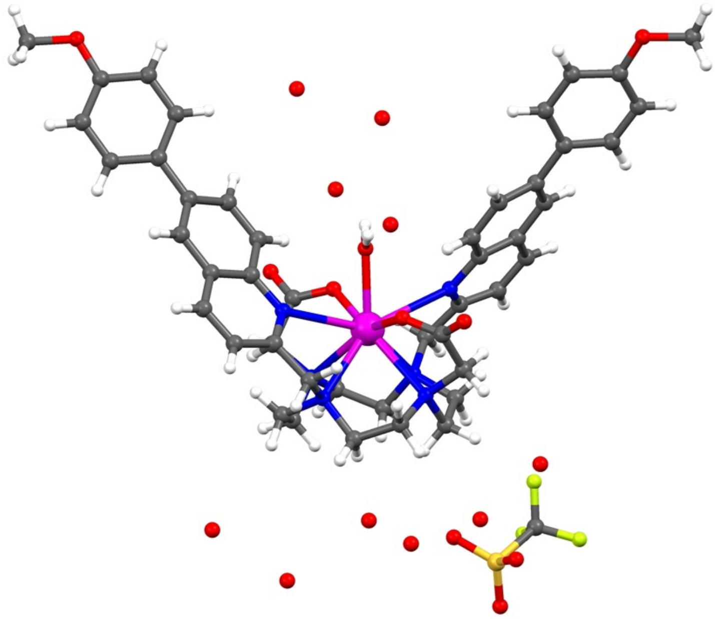

Figure S29. Single crystal X-ray structure of [**Gd.6PhOMe**]$^+$ displaying the asymmetric unit. The lowest occupancy disorder has been omitted for clarity. Atom colours: Gd pink, C grey, N blue, O red, H white, S orange, F lime green.

Table S3. Crystal data and structure refinement details.

**Compound**	**[Gd.6PhOMe]$^+$**
Empirical formula	$C_{47}H_{50}F_3GdN_6O_{20}S$
Formula weight	1265.24
Temperature / K	100(2)
Crystal system	Monoclinic
Space group	$P2_1/c$
Unit cell dimensions: *a* / Å	16.8352(3)
b / Å	17.2648(2)
c / Å	18.6772(3)
α / °	90
β / °	94.300(2)
γ / °	90
Volume / Å^3	5413.37(14)
Z	4
Density (calc.) / cm^3	1.552
Absorption coeff. / mm^{-1}	1.354
F(000)	2564.0
Crystal size / mm^3	0.153 × 0.091 × 0.017
Radiation	Mo K*α* (λ = 0.71075)
Theta range for data / °	3.93 to 50.056
Index ranges	-20 ≤ h ≤ 20, -20 ≤ k ≤ 20, -22 ≤ l ≤ 22
Reflections collected	83075
Independent reflections	9558 (R_{int} = 0.0639, R_{sigma} = 0.0321)
Data/restraints/parameters	9558/400/834
Goodness-of-fit on F^2	1.058
Final R indexes ($I > 2\sigma(I)$)	R_1 = 0.0642, wR_2 = 0.1602
Final R indexes (all data)	R_1 = 0.0929, wR_2 = 0.1864
Largest diff. peak/hole / e Å^{-3}	3.55/-1.10

Table S4. Bond lengths for [**Gd.6PhOMe**]$^+$.

Atom	Atom	Length / Å	Atom	Atom	Length / Å	Atom	Atom	Length / Å
Gd1	O1	2.338(5)	C28	C33	1.389(12)	C27	N5A	1.497(19)
Gd1	O7	2.369(6)	C28	C23	1.481(11)	C38	C37	1.517(14)
Gd1	O3	2.351(6)	C28	C29	1.411(11)	C37	N4A	1.48(2)
Gd1	N1	2.853(6)	C21	C22	1.410(11)	C14	C13	1.403(13)
Gd1	N2	2.889(6)	C21	C20	1.406(11)	C10	N3A	1.509(19)
Gd1	N3	2.679(16)	C16	C15	1.373(13)	C43	C44	1.489(18)
Gd1	N4	2.686(16)	C9	C8	1.418(11)	C45	C46	1.490(18)
Gd1	N5	2.661(16)	C33	C32	1.380(13)	C40	C39	1.517(18)
Gd1	N6	2.699(15)	N3	C10	1.513(12)	C41	C42	1.49(2)
Gd1	N3A	2.66(5)	N3	C46	1.493(19)	C39A	C40A	1.52(4)
Gd1	N6A	2.62(4)	N3	C39	1.491(18)	C39A	N3A	1.47(4)
Gd1	N5A	2.60(5)	C24	C23	1.416(10)	C43A	C44A	1.52(4)
Gd1	N4A	2.65(5)	C6	C5	1.380(11)	C43A	N5A	1.46(5)
O1	C36	1.297(9)	O6	C31	1.356(11)	C41A	C42A	1.52(3)
O3	C38	1.285(10)	O6	C34	1.431(15)	C41A	N4A	1.50(4)
O2	C36	1.219(10)	C22	C23	1.371(11)	C45A	C46A	1.50(4)
N2	C26	1.382(10)	C3	C2	1.342(12)	C45A	N6A	1.47(4)
N2	C18	1.334(10)	C18	C27	1.485(12)	C44A	N6A	1.51(5)
N1	C9	1.383(11)	C1	C2	1.392(11)	C40A	N4A	1.47(5)
N1	C1	1.335(10)	C1	C10	1.491(12)	C46A	N3A	1.45(5)
O4	C38	1.237(11)	N4	C37	1.475(13)	C42A	N5A	1.54(5)
C4	C9	1.410(11)	N4	C40	1.47(2)	C47	F1	1.331(12)
C4	C3	1.418(11)	N4	C41	1.499(17)	C47	F3	1.324(12)
C4	C5	1.393(12)	C36	C35	1.498(12)	C47	F2	1.319(12)
O5	C14	1.374(12)	C12	C13	1.389(13)	C47	S1	1.82(2)
O5	C17	1.447(13)	C15	C14	1.355(14)	S1	O9	1.457(11)
C25	C26	1.406(11)	C35	N6	1.500(12)	S1	O10	1.432(11)
C25	C24	1.372(11)	C35	N6A	1.490(18)	S1	O8A	1.433(12)
C26	C21	1.425(10)	C32	C31	1.389(13)	C47A	F1A	1.330(13)
C19	C18	1.418(10)	N5	C27	1.507(12)	C47A	F3A	1.345(14)
C19	C20	1.351(12)	N5	C43	1.507(18)	C47A	F2A	1.319(13)
C7	C8	1.359(12)	N5	C42	1.496(19)	C47A	S1A	1.759(17)
C7	C6	1.425(10)	N6	C45	1.495(16)	S1A	O8	1.455(13)
C11	C16	1.402(11)	N6	C44	1.469(19)	S1A	O9A	1.445(13)
C11	C6	1.469(12)	C30	C29	1.389(12)	S1A	O10A	1.412(13)
C11	C12	1.392(12)	C30	C31	1.383(14)			

Table S5. Bond angles for [**Gd.6PhOMe**]$^+$.

Atom	Atom	Atom	Angle/˚	Atom	Atom	Atom	Angle/˚
O1	Gd1	O7	70.76(19)	C40	N4	Gd1	116.0(8)
O1	Gd1	O3	142.9(2)	C40	N4	C37	108.5(11)
O1	Gd1	N1	82.00(18)	C40	N4	C41	109.8(13)
O1	Gd1	N3	83.8(2)	C41	N4	Gd1	108.1(9)

O1	Gd1	N4	148.9(3)	O1	C36	C35	115.8(7)
O1	Gd1	N5	120.2(3)	O2	C36	O1	124.6(8)
O1	Gd1	N6	62.5(3)	O2	C36	C35	119.4(7)
O1	Gd1	N3A	73.8(7)	C3	C2	C1	120.3(8)
O1	Gd1	N6A	68.2(5)	C13	C12	C11	121.7(8)
O1	Gd1	N5A	132.5(7)	C14	C15	C16	120.3(8)
O1	Gd1	N4A	138.5(6)	C36	C35	N6	108.4(8)
O7	Gd1	N1	73.1(2)	N6A	C35	C36	118.7(18)
O7	Gd1	N3	129.7(3)	C6	C5	C4	121.7(7)
O7	Gd1	N4	130.3(3)	C33	C32	C31	120.3(9)
O7	Gd1	N5	129.0(3)	C27	N5	Gd1	106.1(9)
O7	Gd1	N6	128.4(2)	C27	N5	C43	112.1(9)
O7	Gd1	N3A	129.3(5)	C43	N5	Gd1	112.9(9)
O7	Gd1	N6A	126.5(5)	C42	N5	Gd1	111.2(8)
O7	Gd1	N5A	128.8(6)	C42	N5	C27	107.7(10)
O7	Gd1	N4A	128.8(6)	C42	N5	C43	106.9(13)
O3	Gd1	O7	72.1(2)	C35	N6	Gd1	102.6(8)
O3	Gd1	N1	86.15(19)	C45	N6	Gd1	110.2(9)
O3	Gd1	N3	120.1(3)	C45	N6	C35	110.9(9)
O3	Gd1	N4	62.7(3)	C44	N6	Gd1	115.0(8)
O3	Gd1	N5	84.8(3)	C44	N6	C35	108.8(10)
O3	Gd1	N6	150.3(3)	C44	N6	C45	109.2(12)
O3	Gd1	N3A	132.3(7)	C31	C30	C29	120.6(8)
O3	Gd1	N6A	140.0(6)	C30	C29	C28	120.9(8)
O3	Gd1	N5A	74.5(7)	C18	C27	N5	107.8(8)
O3	Gd1	N4A	68.5(5)	C18	C27	N5A	123.9(18)
N3	Gd1	N1	60.6(3)	O3	C38	C37	115.9(8)
N3	Gd1	N4	65.2(3)	O4	C38	O3	124.8(10)
N3	Gd1	N6	66.6(3)	O4	C38	C37	119.2(8)
N4	Gd1	N1	83.7(3)	N4	C37	C38	108.1(9)
N4	Gd1	N6	101.4(3)	N4A	C37	C38	119.9(19)
N5	Gd1	N1	151.3(3)	O6	C31	C32	115.8(9)
N5	Gd1	N3	101.3(3)	O6	C31	C30	125.1(9)
N5	Gd1	N4	67.9(3)	C30	C31	C32	119.1(9)
N5	Gd1	N6	65.6(3)	O5	C14	C13	114.2(10)
N6	Gd1	N1	118.4(3)	C15	C14	O5	125.6(9)
N3A	Gd1	N1	67.1(5)	C15	C14	C13	120.2(9)
N6A	Gd1	N1	131.0(7)	C1	C10	N3	108.6(8)
N6A	Gd1	N3A	67.7(8)	C1	C10	N3A	125.4(17)
N6A	Gd1	N4A	104.7(7)	C12	C13	C14	119.0(9)
N5A	Gd1	N1	141.1(6)	C44	C43	N5	112.6(10)
N5A	Gd1	N3A	101.9(8)	C46	C45	N6	112.0(11)
N5A	Gd1	N6A	66.8(9)	C45	C46	N3	111.2(11)
N5A	Gd1	N4A	68.8(9)	N4	C40	C39	111.8(12)
N4A	Gd1	N1	72.8(7)	N3	C39	C40	111.4(11)
N4A	Gd1	N3A	66.2(8)	C42	C41	N4	112.4(11)

C36	O1	Gd1	124.9(5)	N6	C44	C43	112.2(11)
C38	O3	Gd1	125.3(6)	C41	C42	N5	110.9(13)
C26	N2	Gd1	131.2(5)	N3A	C39A	C40A	112(3)
C18	N2	Gd1	112.0(5)	N5A	C43A	C44A	111(3)
C18	N2	C26	116.8(6)	N4A	C41A	C42A	115(3)
C9	N1	Gd1	130.8(5)	N6A	C45A	C46A	113(3)
C1	N1	Gd1	113.2(5)	N6A	C44A	C43A	110(2)
C1	N1	C9	116.0(7)	N4A	C40A	C39A	112(2)
C9	C4	C3	117.6(8)	N3A	C46A	C45A	109(2)
C5	C4	C9	121.1(7)	C41A	C42A	N5A	106(2)
C5	C4	C3	121.4(7)	C10	N3A	Gd1	106(2)
C14	O5	C17	115.0(9)	C39A	N3A	Gd1	114(2)
C24	C25	C26	121.4(7)	C39A	N3A	C10	103(2)
N2	C26	C25	120.4(6)	C46A	N3A	Gd1	110(2)
N2	C26	C21	122.1(7)	C46A	N3A	C10	111(2)
C25	C26	C21	117.5(7)	C46A	N3A	C39A	111(3)
C20	C19	C18	119.7(7)	C35	N6A	Gd1	107(2)
C8	C7	C6	121.5(7)	C35	N6A	C44A	114(2)
C16	C11	C6	121.3(8)	C45A	N6A	Gd1	110(2)
C12	C11	C16	116.7(8)	C45A	N6A	C35	104(2)
C12	C11	C6	122.0(7)	C45A	N6A	C44A	109(3)
C33	C28	C23	121.5(7)	C44A	N6A	Gd1	112(2)
C33	C28	C29	117.2(8)	C27	N5A	Gd1	109(2)
C29	C28	C23	121.3(8)	C27	N5A	C42A	109(2)
C22	C21	C26	119.6(7)	C43A	N5A	Gd1	116(2)
C20	C21	C26	118.4(7)	C43A	N5A	C27	99(2)
C20	C21	C22	121.9(7)	C43A	N5A	C42A	110(3)
C15	C16	C11	122.1(9)	C42A	N5A	Gd1	113(2)
N1	C9	C4	122.8(7)	C37	N4A	Gd1	105(2)
N1	C9	C8	120.4(7)	C37	N4A	C41A	103(2)
C4	C9	C8	116.8(7)	C41A	N4A	Gd1	109(2)
C32	C33	C28	121.9(8)	C40A	N4A	Gd1	113(2)
C10	N3	Gd1	105.2(8)	C40A	N4A	C37	117(3)
C46	N3	Gd1	112.6(7)	C40A	N4A	C41A	108(3)
C46	N3	C10	106.8(10)	F1	C47	S1	106.4(11)
C39	N3	Gd1	113.3(9)	F3	C47	F1	106.0(14)
C39	N3	C10	110.7(9)	F3	C47	S1	116.4(13)
C39	N3	C46	107.9(13)	F2	C47	F1	106.8(14)
C25	C24	C23	121.5(7)	F2	C47	F3	106.7(15)
C7	C8	C9	121.6(7)	F2	C47	S1	113.9(12)
C7	C6	C11	120.7(7)	O9	S1	C47	100.2(8)
C5	C6	C7	117.3(8)	O10	S1	C47	101.2(8)
C5	C6	C11	122.0(7)	O10	S1	O9	116.3(11)
C31	O6	C34	117.5(9)	O10	S1	O8A	115.1(10)
C23	C22	C21	122.1(7)	O8A	S1	C47	104.1(9)
C2	C3	C4	119.0(7)	O8A	S1	O9	116.4(10)

N2	C18	C19	123.8(7)	F1A	C47A	F3A	110.9(18)
N2	C18	C27	117.9(7)	F1A	C47A	S1A	110.3(14)
C19	C18	C27	118.3(7)	F3A	C47A	S1A	99.4(14)
C24	C23	C28	120.2(7)	F2A	C47A	F1A	112.5(16)
C22	C23	C28	122.0(7)	F2A	C47A	F3A	111.4(17)
C22	C23	C24	117.8(7)	F2A	C47A	S1A	111.6(15)
C19	C20	C21	119.2(7)	O8	S1A	C47A	105.6(11)
N1	C1	C2	124.1(8)	O9A	S1A	C47A	114.4(12)
N1	C1	C10	117.5(7)	O9A	S1A	O8	98.9(14)
C2	C1	C10	118.4(7)	O10A	S1A	C47A	109.8(12)
C37	N4	Gd1	104.1(9)	O10A	S1A	O8	114.3(15)
C37	N4	C41	110.1(10)	O10A	S1A	O9A	113.3(15)

Table S6. Fractional Atomic Coordinates and Equivalent Isotropic Displacement Parameters (Å2) for [**Gd.6PhOMe**]$^{+}$.

Atom	x	y	z	U(eq)
Gd1	3104.1(2)	4987.8(2)	2355.9(2)	34.46(14)
O1	3576(3)	3747(3)	2654(3)	42.0(13)
O7	4515(4)	5006(3)	2469(3)	50.2(14)
O3	3520(4)	6264(3)	2153(3)	51.0(15)
O2	3766(4)	2520(3)	2338(3)	54.8(16)
N2	3650(4)	4706(4)	949(3)	36.8(14)
N1	3573(4)	5268(4)	3833(3)	41.5(16)
O4	3579(5)	7516(3)	2454(3)	71(2)
C4	4352(5)	5757(4)	4900(4)	41.7(19)
O5	8108(5)	8340(4)	5931(4)	78(2)
C25	4743(5)	3822(4)	1237(4)	38.5(18)
C26	4263(5)	4251(4)	732(4)	37.5(17)
C19	3357(5)	5054(4)	-302(4)	38.8(17)
C7	5274(5)	6608(4)	4019(4)	40.0(18)
C11	6156(5)	7063(4)	5083(4)	41.7(19)
C28	6279(5)	2918(4)	120(4)	40.9(18)
C21	4434(5)	4194(4)	-2(4)	36.5(17)
C16	6143(6)	7419(4)	5757(4)	44(2)
C9	4169(5)	5731(4)	4151(4)	40.2(18)
C33	6965(6)	2880(5)	580(4)	49(2)
N3	2072(9)	4700(6)	3348(6)	45(3)
C24	5378(5)	3395(4)	1038(4)	37.6(17)
C8	4650(5)	6188(4)	3723(4)	44(2)
C6	5472(5)	6614(4)	4774(4)	39.7(18)
O6	8321(5)	1732(5)	-381(4)	81(2)
C22	5090(5)	3749(4)	-189(4)	38.6(18)
C3	3858(6)	5329(5)	5338(4)	47(2)
C18	3223(5)	5095(4)	438(4)	39.0(17)
C23	5569(5)	3355(4)	314(4)	37.9(18)
C20	3956(5)	4610(4)	-517(4)	43(2)
C1	3156(5)	4856(4)	4280(4)	41.0(18)
N4	2051(9)	6127(7)	2538(6)	51(3)

C36	3388(5)	3123(5)	2293(4)	43.2(19)
C2	3269(5)	4895(4)	5025(4)	43.4(19)
C12	6853(6)	7139(5)	4733(5)	49(2)
C15	6777(6)	7841(5)	6053(4)	52(2)
C35	2684(5)	3187(4)	1756(5)	46(2)
C5	4992(5)	6191(4)	5197(4)	45(2)
C32	7634(6)	2486(5)	399(5)	55(2)
N5	2121(9)	5206(6)	1190(6)	46(3)
N6	2126(8)	3782(7)	2019(7)	45(3)
C30	6962(6)	2134(5)	-726(5)	52(2)
C29	6291(5)	2535(5)	-546(4)	45.0(19)
C27	2597(6)	5630(5)	661(4)	53(2)
C38	3261(6)	6871(5)	2458(4)	54(2)
C37	2535(7)	6755(5)	2881(5)	61(3)
C31	7637(6)	2106(6)	-257(5)	60(3)
C14	7445(7)	7921(5)	5697(5)	61(3)
C10	2556(5)	4299(5)	3955(4)	50(2)
C13	7500(6)	7559(5)	5030(5)	55(2)
C17	8043(8)	8756(7)	6597(6)	92(4)
C34	8334(9)	1301(9)	-1035(7)	106(5)
C43	1774(9)	4461(8)	884(7)	45(3)
C45	1753(9)	3502(8)	2673(7)	45(3)
C46	1429(8)	4151(8)	3088(6)	48(3)
C40	1406(9)	5941(7)	2995(8)	56(3)
C39	1687(10)	5412(10)	3611(8)	57(4)
C41	1708(10)	6378(8)	1811(8)	57(4)
C44	1498(8)	3937(7)	1448(7)	50(3)
C42	1435(8)	5712(8)	1350(7)	55(3)
C39A	1441(18)	4914(15)	3394(13)	38(2)
C43A	1541(18)	4966(16)	1009(13)	38(2)
C41A	1452(19)	6174(15)	2242(14)	38(2)
C45A	1536(18)	3730(15)	2181(13)	38(2)
C44A	1870(20)	4143(18)	995(17)	38(2)
C40A	1710(20)	5752(19)	3464(18)	38(2)
C46A	1790(20)	3650(20)	2965(16)	38(2)
C42A	1740(20)	6280(20)	1497(16)	38(2)
N3A	2100(30)	4395(18)	3236(13)	45(3)
N6A	2200(20)	3909(16)	1739(18)	45(3)
N5A	2140(30)	5508(19)	1310(16)	46(3)
N4A	2090(30)	6014(16)	2826(18)	51(3)
C47	-696(9)	5990(8)	4491(10)	102(3)
F1	-962(9)	5312(8)	4714(7)	109(2)
F3	91(9)	5954(10)	4581(9)	109(2)
F2	-924(8)	6520(8)	4941(6)	109(2)
S1	-1122(6)	6116(5)	3571(5)	116(2)
O9	-1956(8)	5952(9)	3666(8)	116(2)
O10	-698(10)	5537(9)	3204(7)	116(2)
O8A	-931(10)	6900(7)	3400(8)	116(2)
C47A	-489(12)	5781(10)	4315(9)	102(3)
F1A	-344(13)	5079(10)	4062(11)	109(2)
F3A	195(13)	6162(15)	4494(13)	109(2)

F2A	-952(13)	5762(14)	4854(10)	109(2)
S1A	-884(9)	6384(8)	3617(8)	116(2)
O8	-849(15)	7166(10)	3908(11)	116(2)
O9A	-1736(11)	6320(15)	3467(12)	116(2)
O10A	-454(14)	6282(14)	3003(9)	116(2)
O12	5254(5)	3566(5)	3031(4)	66(3)
O14	7253(16)	3935(8)	2228(6)	94(7)
O11	5230(11)	6404(11)	2017(7)	75(7)
O11A	5710(12)	5988(10)	2228(9)	51(7)
O13	6798(11)	5260(10)	2885(10)	136(8)
O13A	6940(20)	5989(18)	3114(17)	101(15)
O12A	5190(30)	2670(30)	2960(20)	66(17)
O17	-269(8)	4899(8)	2043(9)	167(6)
O19	105(12)	7630(13)	2275(11)	149(8)
O16	-1220(40)	4170(30)	850(30)	170(30)
O20	1080(20)	8470(20)	2960(20)	229(18)
O15	-375(13)	3037(16)	303(15)	207(11)
O20A	-1148(19)	4260(20)	2269(18)	136(17)
O18A	-367(13)	6600(20)	825(16)	127(14)
O18	-120(20)	7430(20)	1023(19)	239(18)
O19A	-570(40)	3300(40)	2610(30)	180(20)
O14A	2250(70)	8790(40)	2880(40)	110(30)
O15A	340(18)	7530(19)	3553(18)	113(13)
O16A	-451(16)	4705(19)	639(16)	246(16)

Table S7. Hydrogen Bonds for [**Gd.6PhOMe**]$^+$.

D	H	A	d(D-H) / Å	d(H-A) / Å	d(D-A) / Å	(D-H…A) / °
O7	H7A	O11	0.87	2.027(15)	2.938(10)	151.6(4)
O7	H7A	O11A	0.87	1.862(15)	2.694(16)	159.5(8)
O7	H7B	O12	0.87	2.143(8)	2.854(15)	158.4(7)
C29	H29	O12A	0.95	2.38(5)	3.25(5)	151.3(13)
C34	H34C	F1A	0.98	2.421(19)	3.26(2)	142.9(10)

Table S8. Atomic Occupancy for [**Gd.6PhOMe**]$^+$.

Atom	Occupancy	Atom	Occupancy	Atom	Occupancy
N3	0.7	N4	0.7	H35A	0.7
H35B	0.7	H35C	0.3	H35D	0.3
N5	0.7	N6	0.7	H27A	0.7
H27B	0.7	H27C	0.3	H27D	0.3
H37A	0.7	H37B	0.7	H37C	0.3
H37D	0.3	H10A	0.7	H10B	0.7
H10C	0.3	H10D	0.3	C43	0.7
H43A	0.7	H43B	0.7	C45	0.7
H45A	0.7	H45B	0.7	C46	0.7
H46A	0.7	H46B	0.7	C40	0.7
H40A	0.7	H40B	0.7	C39	0.7
H39A	0.7	H39B	0.7	C41	0.7
H41A	0.7	H41B	0.7	C44	0.7
H44A	0.7	H44B	0.7	C42	0.7

H42A	0.7	H42B	0.7	C39A	0.3
H39C	0.3	H39D	0.3	C43A	0.3
H43C	0.3	H43D	0.3	C41A	0.3
H41C	0.3	H41D	0.3	C45A	0.3
H45C	0.3	H45D	0.3	C44A	0.3
H44C	0.3	H44D	0.3	C40A	0.3
H40C	0.3	H40D	0.3	C46A	0.3
H46C	0.3	H46D	0.3	C42A	0.3
H42C	0.3	H42D	0.3	N3A	0.3
N6A	0.3	N5A	0.3	N4A	0.3
C47	0.612(6)	F1	0.612(6)	F3	0.612(6)
F2	0.612(6)	S1	0.612(6)	O9	0.612(6)
O10	0.612(6)	O8A	0.612(6)	C47A	0.388(6)
F1A	0.388(6)	F3A	0.388(6)	F2A	0.388(6)
S1A	0.388(6)	O8	0.388(6)	O9A	0.388(6)
O10A	0.388(6)	O12	0.866(16)	O14	0.82(6)
O11	0.59(3)	O11A	0.41(3)	O13	0.72(3)
O13A	0.28(3)	O12A	0.134(16)	O19	0.683(19)
O16	0.26(3)	O20	0.61(4)	O15	0.683(19)
O20A	0.39(4)	O18A	0.40(3)	O18	0.60(3)
O19A	0.317(19)	O14A	0.18(6)	O15A	0.317(19)
O16A	0.74(3)				

Table S9. Face-to-face π-π interactions observed in the packing of [**Gd.6PhOMe**]$^{+}$.

Interaction	centroid-to-centroid / Å	plane-to-plane shift / Å	plane-to-centroid / Å
quinoline ··· quinoline	3.883(5)	2.058(11)	3.293(8)
quinoline ··· quinoline	3.760(5)	1.839(11)	3.280(7)
quinoline ··· phenyl	3.958(5)	1.519(12)	3.568(7)

6. References

1 K. Suzuki, A. Kobayashi, S. Kaneko, K. Takehira, T. Yoshihara, H. Ishida, Y. Shiina, S. Oishi and S. Tobita, *Phys. Chem. Chem. Phys.*, 2009, **11**, 9850–9860.

2 A. Beeby, I. M. Clarkson, R. S. Dickins, S. Faulkner, D. Parker, L. Royle, A. S. De Sousa, J. A. G. Williams and M. Woods, *J. Chem. Soc., Perkin Trans. 2*, 1999, **2**, 493–503.

3 G. M. Sheldrick, *Acta Crystallogr. A*, 2015, **71**, 3–8.

4 G. M. Sheldrick, *Acta Crystallogr. C*, 2015, **71**, 3–8.

5 O. V. Dolomanov, L. J. Bourhis, R. J. Gildea, J. A. K. Howard and H. Puschmann, *J. Appl. Crystallogr.*, 2009, **42**, 339–341.

6 S. Grimme, A. Hansen, S. Ehlert and J. M. Mewes, *J. Chem. Phys.*, 2021, **154**, 064103.

7 F. Neese, *Wiley Interdiscip. Rev. Comput. Mol. Sci.*, 2022, **12**, e1606.

8 A. V. Marenich, C. J. Cramer and D. G. Truhlar, *J. Phys. Chem. B*, 2009, **113**, 6378–6396.

9 K. Sénéchal-David, A. Hemeryck, N. Tancrez, L. Toupet, J. A. G. Williams, I. Ledoux, J. Zyss, A. Boucekkine, J. P. Guéan, H. Le Bozec and O. Maury, *J. Am. Chem. Soc.*, 2006, **128**, 12243–12255.

10 E. R. Neil, M. A. Fox, R. Pal and D. Parker, *Dalton Trans.*, 2016, **45**, 8355–8366.

11 R. Pal, *Faraday Discuss.*, 2015, **177**, 507–515.
